# LGR5-positive fibroblasts orchestrate skin mechanoadaptation through JAK1 modulation

**DOI:** 10.1038/s41467-026-77113-y

**Published:** 2026-09-11

**Authors:** Qiaoyu Fu, Xi Cheng, Nuo Chen, Yidan Sun, Luwen Xu, Yu Cheng, Chen Wang, Yin Li, Tingting Yu, Yuao Yan, Wenwan Zhang, Yu Bu, Lifang Lei, Yining Chen, Zhen Li, Peiying Zhu, Caibing Wang, Liang Zhang, Caiyue Liu, Qingfeng Li

**Affiliations:** 1 Department of Plastic and Reconstructive Surgery, Shanghai Ninth People’s Hospital, Shanghai Jiao Tong University School of Medicine, Shanghai, China; 2 Shanghai Institute of Nutrition and Health, University of Chinese Academy of Sciences, Chinese Academy of Sciences, Shanghai, China; 3 Shanghai Key Laboratory of Reproductive Medicine, Shanghai, China

**Keywords:** Cell signalling, Molecular medicine, Molecular biology, Physiology, Target identification

## Abstract

Mechanical adaptation is a fundamental yet poorly understood process in stromal tissue biology. Here, we identify a population of LGR5^+^ mechanically induced regulatory fibroblasts (LGR5^+^ MIRFs) in mouse and human dermis, whose induction requires a YAP/AP-1-dependent transcriptional program. LGR5^+^ MIRFs channel mechanical input toward adaptive tissue remodeling by coordinating extracellular matrix reorganization while restraining inflammation. Their ablation or fibroblast-specific deletion of *Lgr5* converts adaptive stretching into pathological fibro-inflammation, while dermal *Lgr5* overexpression enhances tissue extensibility. Mechanistically, we uncover a non-canonical LGR5-JAK1 interaction that suppresses JAK-STAT signaling, directly coupling mechanosensing to kinase pathway activity. Topical JAK1 inhibition recapitulates the pro-remodeling effects of LGR5^*+*^ MIRFs, suggesting a translatable therapeutic strategy for optimizing skin expansion, treating striae, and mitigating fibrotic disorders. Our work redefines LGR5 as a conserved stromal mechanoresponder and provides a new conceptual framework for understanding adaptive tissue plasticity under mechanical stress.

## Abstract (web summary)

Mechanical stress induces LGR5+ fibroblasts that promote adaptive tissue remodeling by limiting fibrosis through JAK1 modulation. The study reveals a conserved mechanism of adaptation to mechanical stress and suggests a strategy for regenerative therapy.

## Introduction

The ability of stromal tissues to adapt to diverse mechanical challenges is essential for maintaining structural integrity and function across organs. The skin exemplifies this stromal mechanoplasticity, dynamically responding to forces ranging from gradual expansion to acute injury while preserving its barrier function. This adaptive duality supports physiological processes such as rapid growth, pregnancy, and weight gain, where controlled expansion occurs, and in chronically stressed regions like plantar skin, where structural remodeling ensures durability^1–3^. Mechanical forces also critically guide wound healing by modulating fibroblast activity and tissue remodeling^4^. Clinically, this plasticity underpins skin expansion techniques, where controlled stretch generates new skin for reconstructive surgery^5^. However, when adaptation fails, the same mechanical cues can drive pathological outcomes including striae, pressure ulcers, and impaired wound healing, as well as local complications in tissue expansion^6^. This paradox underscores a fundamental gap in understanding how stromal mechanobiology navigates adaptive remodeling versus maladaptive fibro-inflammatory outcomes^7^.

Central to this paradox lies the unresolved molecular logic by which dermal fibroblasts decode mechanical cues into divergent remodeling fates. While epidermal stem cells undergo proliferative expansion via lineage-biased renewal^8–12^, the dermis remodels primarily through fibroblast functional adaptation and extracellular matrix (ECM) reorganization. Unlike the intrinsically pro-regenerative fibroblasts of prenatal skin or reindeer velvet^13,14^, postnatal dermal fibroblasts—notably *Engrailed-1*^*+*^ lineages—activate a YAP/TAZ-centered mechanotransduction cascade that favors scarring over regeneration^15–17^. This drives a profibrotic program (e.g., α-SMA, collagens, and matricellular proteins) that promotes ECM deposition and stiffening, thereby amplifying YAP/TAZ signaling in a self-reinforcing loop^18–23^. This circuit is also integrated with soluble pathways such as the TGF-β–Wnt–Ang II cycle^24^. Beyond this cell‑autonomous loop, mechanical stimulation triggers a robust fibro-inflammatory circuit wherein fibroblasts recruit and polarize macrophages via both paracrine mediators and physical cues, and macrophages reciprocally enhance fibrogenesis^24–30^. This bidirectional crosstalk consolidates a pathologic feedforward circuit that drives excessive collagen deposition, scarring, and reduced compliance. Yet this established framework centered on amplifying fibro-inflammatory feedforward circuits conflicts with the dermis’s ability (e.g., physiological expansion) to undergo adaptive remodeling without progressing to widespread fibrosis. This implies the existence of a mechanically induced regulatory program that constrains the fibro-inflammatory loop. The identity of this braking system and its integration into the existing mechanosignaling network remain undefined, representing a key gap in linking mechano-induced heterogeneity to controlled outcomes.

Here, we identify LGR5, best known as an epithelial stem cell marker^31–33^, as the defining feature of a conserved mechanically induced state of dermal fibroblasts that function as precisely such a brake. We term these cells LGR5^*+*^ mechanically induced regulatory fibroblasts (LGR5^+^ MIRFs). We demonstrate that mechanical stress induces LGR5 expression in fibroblasts through a YAP/AP-1–dependent enhancer mechanism, and that LGR5 in turn cell-autonomously suppresses JAK-STAT signaling to fine-tune tissue remodeling. Our work establishes LGR5^*+*^ MIRFs as a core mechanistic link between physical cues and regenerative outcomes and unveils a targetable pathway to promote mechanical adaptation while constraining fibro-inflammatory responses.

## Results

### LGR5^*+*^ MIRFs emerge during skin mechanoadaptation

**Fig. 1:**
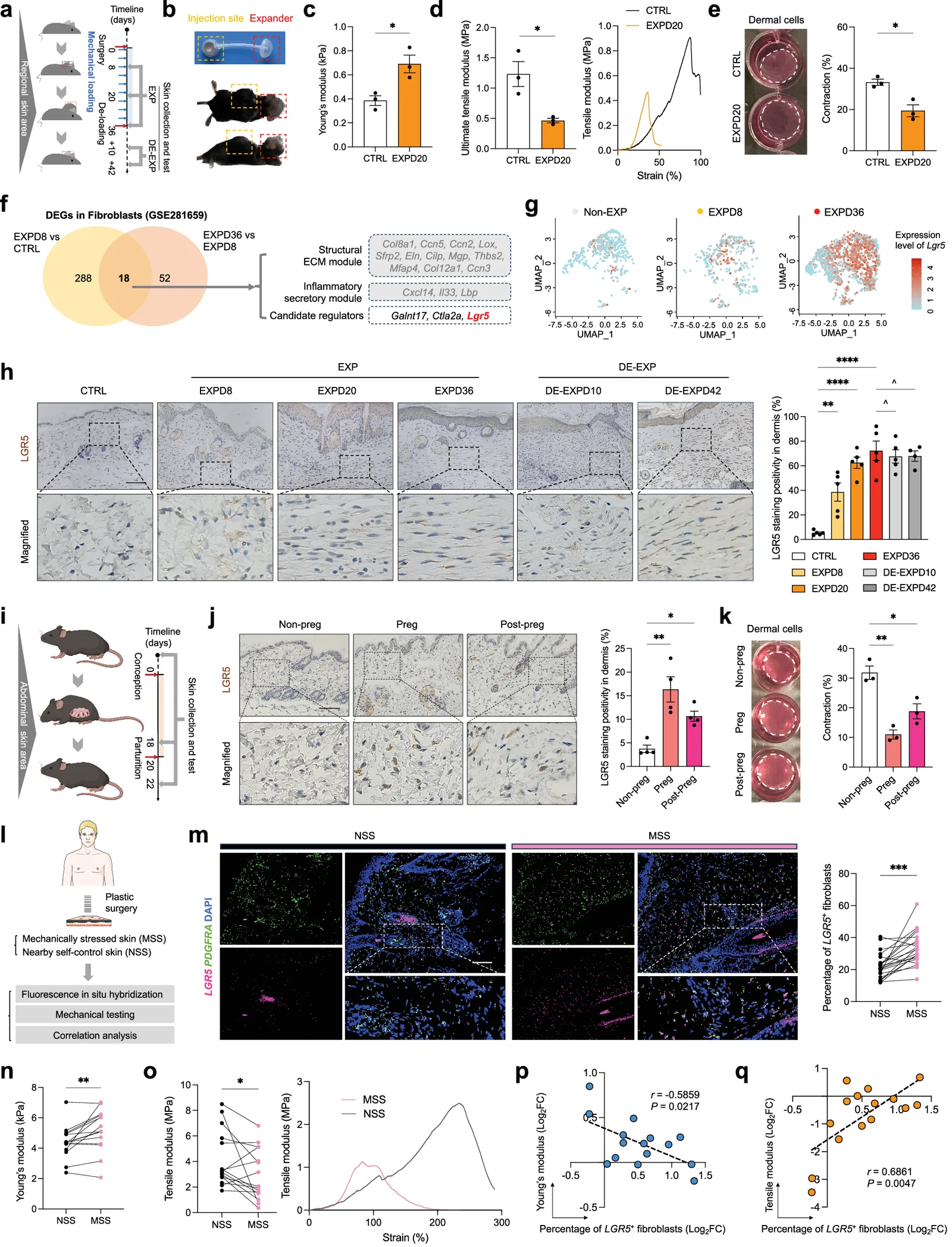
Mechanical stretch induces LGR5^*+*^ fibroblasts across murine and human skin. **a** Schematic timeline for the expansion model. EXP, expanded skin. DE-EXP, de-loaded expanded skin. **b** Diagram of the expansion model. **c** Quantification of tissue stiffness (*n* = 3 mice) (*P = 0.0219). CTRL, non-expanded skin. EXPD, days post skin expansion. **d** Biomechanical assessment of tensile strength (*n* = 3 mice). Left, quantification (*P = 0.0216); right, representative curves. **e** Matrix contraction assay (*n* = 3 mice). Left, representative images; right, quantification of contraction percentage (*P = 0.0129). **f** Workflow for identifying candidate regulators. DEGs, differentially expressed genes. **g** UMAP of *Lgr5* expression in fibroblasts. **h** IHC staining for LGR5 (*n* = 4–5 mice). Left, representative images (scale bar, 100 μm); right, quantification of LGR5^*+*^ dermal cells (^*P* = 0.9951 / 0.9907, **P = 0.0040, ****P < 0.0001). DE-EXPD, days post de-loading. **i** Schematic timeline of abdominal skin collection. Created in BioRender. Cheng, X. (2026) https://BioRender.com/dtz7ir2. **j** IHC staining for LGR5 in skin of non-pregnant (Non-preg), pregnant (Preg) and post-pregnant mice (Post-preg) (*n* = 4 mice). Left, representative images (scale bar, 100 μm); right, quantification of LGR5^*+*^ dermal cells (*P = 0.0478, **P = 0.0016). **k** Matrix contraction assay (*n* = 3 mice). Left, representative images; right, quantification of contraction percentage (*P = 0.0123, **P = 0.0012). **l** Schematic timeline for human sample collection and analysis. MSS, mechanically stressed skin; NSS, nearby self-control skin. Images provided by Servier Medical Art (https://smart.servier.com), licensed under CC BY 4.0 (https://creativecommons.org/licenses/by/4.0/). **m** RNA in situ hybridization for *LGR5* and *PDGFRA* (*n* = 21 pairs). Left, representative images (scale bar, 100 μm); right, quantification of *LGR5*^*+*^ fibroblasts (***P = 0.0002). **n** Quantification of tissue stiffness (*n* = 15 pairs) (**P = 0.0054). **o** Biomechanical assessment of tensile strength (*n* = 15 pairs). Left, quantification (*P = 0.0486); right, representative curves. **p** Correlation between log_2_ fold change (MSS/NSS) in tissue stiffness and the percentage of *LGR5*^*+*^ fibroblasts (*n* = 15 pairs). **q** Correlation between log_2_ fold change (MSS/NSS) in tensile strength and the percentage of *LGR5*^+^ fibroblasts (*n* = 15 pairs). Data are presented as mean ± SEM. Statistical significance was determined as follows: **c–e** two-tailed unpaired Student’s *t*-test; (**h, j, k**) one-way ANOVA with Tukey’s post hoc test; **m–o** two-tailed paired Student’s *t*-test; **p, q** two-tailed Pearson correlation analysis. Source data are provided as a Source Data file.

To investigate skin mechanoadaptation, we employed a mouse expansion model in which skin was subjected to progressive mechanical stretch^34^ (Fig. 1a, b). This system recapitulates key features of skin mechanobiology: the epidermis responds through marked proliferation of basal cells and tissue thickening, whereas the load-bearing dermis undergoes extensive, persistent remodeling with less pronounced changes in fibroblast proliferation, indicating a primary shift in cellular function (Supplementary Fig. 1a–g). At the tissue level, expanded skin exhibited dermal thinning, fibrosis, and inflammation relative to non-expanded skin (Supplementary Fig. 1d–h). Notably, these histological hallmarks persisted after load removal, reflecting a state of long-term tissue remodeling (Supplementary Fig. 1a–e). Concomitantly, expanded skin exhibited profound biomechanical alterations, characterized by increased tissue stiffness (Fig. 1c), reduced ultimate tensile strength (Fig. 1d), and reduced matrix contractility of primary dermal cells ex vivo (Fig. 1e). This established model thus provides a controlled system for dissecting cellular and tissue-level responses to mechanical loading.

We next sought to identify key regulators driving these adaptive changes. Fibroblasts being the principal matrix-producing and remodeling cells in the dermis, we re-analyzed our published single-cell RNA-sequencing (scRNA-seq) dataset from expanded mouse skin^34^, focusing on the fibroblast compartment. Among genes sequentially upregulated with increasing mechanical load, only three—*Galnt17*, *Ctla2a*, and *Lgr5—*encoded neither structural ECM components nor secreted inflammatory factors, nominating them as candidate core regulators (Fig. 1f). We focused on *Lgr5*, a leucine-rich repeat-containing G-protein-coupled receptor that typically marks stem cells in rapidly renewing epithelia^31–33^, due to its unexpected induction in fibroblasts under mechanical loading (Fig. 1g). Immunostaining confirmed the progressive, load-dependent accumulation of elongated LGR5^*+*^ fibroblasts, a population that persisted after cessation of mechanical loading, implicating them in sustained tissue remodeling (Fig. 1h). This mechanoresponsive induction was independently validated by spatial transcriptomics in another mouse skin expansion model with distinct loading parameters^12^ (Supplementary Fig. 2a).

To determine whether LGR5^+^ fibroblasts represent a general response to physiological stretch, we analyzed abdominal skin from perinatal pregnancy, a model closely mirroring our experimental stretch-and-release paradigm (Fig. 1i). As expected, LGR5^+^ cells were detected in the dermis during late gestation and remained present two days postpartum (Fig. 1j). Concomitantly, dermal cells in these samples exhibited reduced matrix contractility (Fig. 1k). Similarly, models of rapid weight gain and early postnatal skin development both upregulated stromal LGR5 expression and triggered reduced matrix contractility (Supplementary Fig. 2b–h). scRNA-seq analysis of neonatal mouse skin further identified a specific fibroblast population, active during early development, that highly co-expresses LGR5 along with stress-responsive, inflammatory, and fibrotic genes (Supplementary Fig. 2i, j). This population was functionally enriched for pathways involved in mechanical stimulus responses and matrix remodeling (Supplementary Fig. 2k).

We next asked whether this mechanoresponsive population is conserved in humans. We analyzed human mechanically stressed skin (MSS; from tissue expansion or striae) alongside unstressed nearby self-control skin (NSS) (Fig. 1l). Two-color RNA in situ hybridization demonstrated a significant enrichment of *PDGFRA*^+^ *LGR5*^*+*^ fibroblasts in human MSS relative to NSS (Fig. 1m). Mirroring our murine findings, human MSS exhibited higher tissue stiffness and lower tensile strength (Fig. 1n, o). Strikingly, the relative abundance of LGR5^*+*^ fibroblasts correlated negatively with tissue stiffness and positively with ultimate tensile strength (Fig. 1p, q), suggesting a protective role for this subset in counteracting detrimental mechanical alterations.

Thus, LGR5 consistently marks a mechanically induced state of fibroblasts in mouse and human skin. Given its specific induction by mechanical stress and critical role in coordinating tissue adaptation as demonstrated below, we refer to this population as LGR5^*+*^ mechanically induced regulatory fibroblasts (LGR5^*+*^ MIRFs).

### Mechanical force induces functional *Lgr5*^+^ MIRFs

**Fig. 2:**
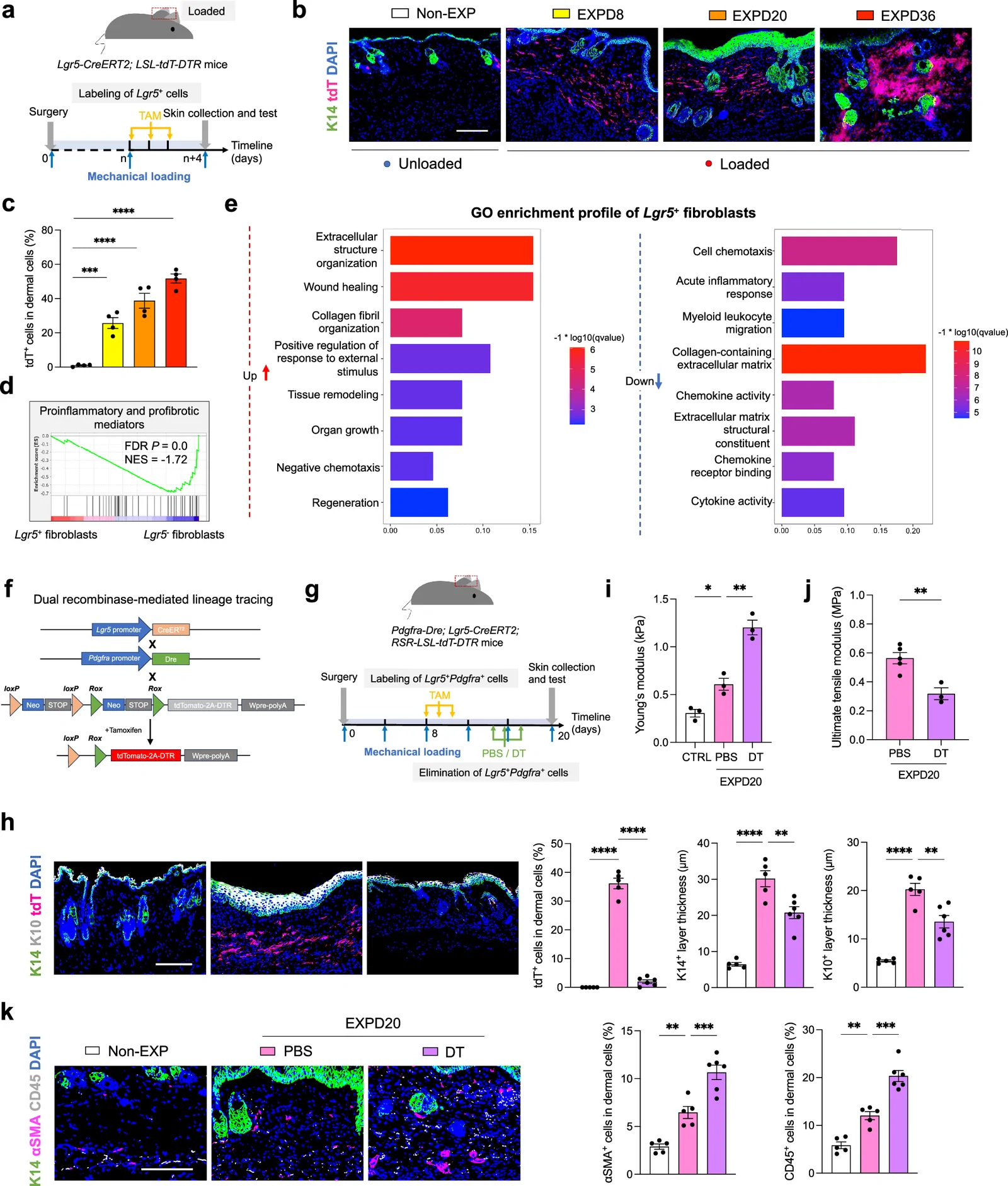
*Lgr5*^*+*^ MIRFs emerge as functional regulators of skin mechanoadaptation. **a** Schematic timeline for labeling *Lgr5*^*+*^ cells in *Lgr5*-tracing mice following skin expansion. **b** Immunofluorescence staining for K14 and tdTomato (tdT) in skin sections from indicated mice (scale bar, 100 μm). **c** Quantification of tdT^+^ cells (*n* = 4 mice) (***P = 0.0004, ****P < 0.0001). **d** GSEA plot showing the significantly downregulated gene set in *Lgr5*^*+*^ fibroblasts. **e** Bar plots showing the significantly upregulated and downregulated GO terms in *Lgr5*^*+*^ fibroblasts based on scRNA-seq data. **f** Schematic of the dual recombinase strategy in *Lgr5*-DTR mice. **g** Schematic timeline for labeling and eliminating *Lgr5*^*+*^ MIRFs in *Lgr5*-DTR mice following skin expansion. **h** Immunofluorescence staining for K14, K10 and tdT (*n* = 5–6 mice). Left, representative images (scale bar, 100 μm); right, quantification of tdT^+^ cells in dermal cells (****P < 0.0001), K14^+^ layer thickness (**P = 0.0032, ****P < 0.0001) and K10^+^ layer thickness (**P = 0.0021, ****P < 0.0001). **i** Quantification of tissue stiffness (*n* = 3 mice) (*P = 0.0311, **P = 0.0012). **j** Biomechanical assessment of tensile strength (*n* = 3–5 mice) (**P = 0.0061). **k** Immunofluorescence staining for K14, αSMA and CD45 (*n* = 5–6 mice). Left, representative images (scale bar, 100 μm); right, quantification of αSMA^+^ cells (**P = 0.0044, ***P = 0.0009) and CD45^+^ cells (**P = 0.0021, ***P = 0.0001). Data are presented as mean ± SEM. Statistical significance was determined by one-way ANOVA with Tukey’s post hoc test or two-tailed unpaired Student’s *t*-test. Source data are provided as a Source Data file.

We next determined the origin and function of *Lgr5*^*+*^ MIRFs using in vivo genetic approaches. Lineage tracing using *Lgr5-CreERT2; Rosa26*-*LSL-tdTomato-DTR* mice (hereafter *Lgr5*-tracing mice) demonstrated robust induction and force-dependent enrichment of *Lgr5*^*+*^ stromal cells in response to sequential mechanical loading (Fig. 2a–c and Supplementary Fig. 3a). These tdTomato^+^ cells co-localized with reticular fibroblast markers and were distinct from other dermal stromal lineages (Supplementary Fig. 3b, c). Critically, tamoxifen induction prior to mechanical loading yielded no labeled stromal cells, confirming their de novo induction from *Lgr5*^-^ precursors by mechanical force (Supplementary Fig. 3d, e).

We next asked whether *Lgr5* induction was a direct mechanoresponse or secondary to other processes associated with tissue mechanoadaptation (e.g., inflammation, fibrosis, aging, or cellular senescence)^12,34,35^. Across a panel of control models encompassing inflammation, fibrosis, natural aging, and cellular senescence, none upregulated fibroblastic *Lgr5* expression (Supplementary Fig. 3f–m), ruling out these factors as primary inducers.

To preliminarily define the functional necessity of *Lgr5*^+^ fibroblasts, we analyzed our scRNA-seq data. Comparative transcriptomic analysis revealed that *Lgr5*^+^ fibroblasts under stretch were enriched for tissue remodeling signatures while suppressing pro-fibrotic and inflammatory pathways compared to *Lgr5*^-^ counterparts (Fig. 2d, e). Furthermore, to definitively target the fibroblast pool, we employed a dual recombinase-mediated genetic lineage tracing and functional ablation, a technique with superior precision^36^. Specifically, by generating *Pdgfra-Dre; Lgr5-CreERT2*; *Rosa26*-*RSR-LSL-tdTomato-DTR* mouse lines (hereafter *Lgr5*-DTR mice) (Fig. 2f), we specifically labeled and confirmed the induction of *Lgr5*^*+*^ MIRFs under stretch (Fig. 2g, h). Diphtheria toxin (DT)-mediated ablation of *Lgr5*^*+*^ MIRFs severely compromised skin mechanical properties, leading to increased tissue stiffness and reduced ultimate tensile strength (Fig. 2i, j). Histologically, ablation triggered pronounced dermal fibrosis and inflammation, alongside impaired epidermal regeneration (Fig. 2h, k). Collectively, these approaches establish *Lgr5*^*+*^ MIRFs as de novo mechano-induced effectors essential for skin mechanoadaptation.

### *Lgr5*^*+*^ MIRFs regulate diverse mechanoadaptive contexts

**Fig. 3:**
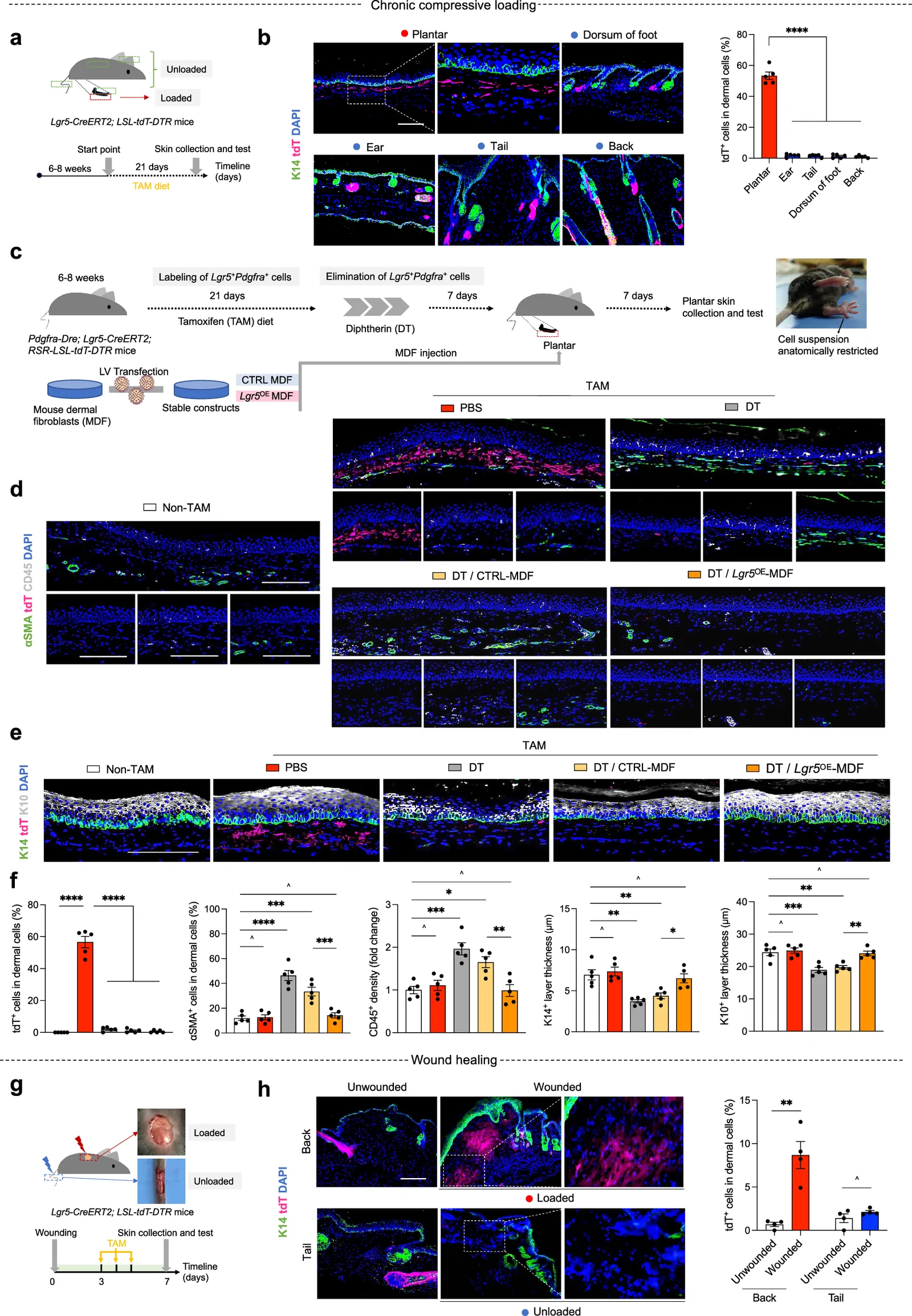
Conservation of *Lgr5*^*+*^ MIRFs in mechanically loaded niches. **a** Schematic timeline for tracing *Lgr5*^*+*^ cells in different anatomical regions of *Lgr5*-tracing mice under homeostasis. **b** Immunofluorescence staining for K14 and tdT in skin sections from indicated regions of *Lgr5*-tracing mice (*n* = 5 mice). Left, representative images (scale bar, 100 μm); right, quantification of tdT^+^ cells (****P < 0.0001). **c** Schematic timeline for labeling and eliminating *Lgr5*^*+*^ MIRFs in *Lgr5*-DTR mice, followed by rescue with either control (CTRL) or *Lgr5*-overexpressing (*Lgr5*^OE^) mouse dermal fibroblasts (MDFs). Images provided by Servier Medical Art (https://smart.servier.com), licensed under CC BY 4.0 (https://creativecommons.org/licenses/by/4.0/). **d** Immunofluorescence staining for tdT, αSMA and CD45 in skin sections from indicated mice (scale bar, 100 μm). **e** Immunofluorescence staining for tdT, K14 and K10 in skin sections from indicated mice (scale bar, 100 μm). **f** Quantification of tdT^+^ cells (****P < 0.0001), αSMA^+^ cells (^*P* = 0.9804 / 0.9998, ***P = 0.0003 / 0.0010, ****P < 0.0001), CD45^+^ cells (^*P* = 0.9999 / 0.9705, *P = 0.0108, **P = 0.0093, ***P = 0.0002), K14^+^ layer thickness (^*P* = 0.9699 / 0.9750, *P = 0.0357, **P = 0.0093 / 0.0010) and K10^+^ layer thickness (^*P* = 0.9984 / 0.9869, **P = 0.0040 / 0.0076, ***P = 0.0008) (*n* = 5 mice). **g** Schematic timeline for tracing *Lgr5*^*+*^ cells in *Lgr5*-tracing mice during wound healing. **h** Immunofluorescence staining for K14 and tdT in skin sections from indicated mice (*n* = 4 mice). Left, representative images (scale bar, 100 μm); right, quantification of tdT^+^ cells (^*P* = 0.2284, **P = 0.0023). Data are presented as mean ± SEM. Statistical significance was determined by one-way ANOVA with Tukey’s post hoc test or two-tailed unpaired Student’s *t*-test. Source data are provided as a Source Data file.

To determine if *Lgr5*^*+*^ MIRFs, previously linked to progressive stretch, function as core regulatory nodes of mechanoadaptation across systems, we developed a multimodal framework to assess their response to diverse mechanical stressors. This approach incorporated two additional force modalities. First, in the mechanically stressed plantar skin, both *Lgr5*-tracing and *Lgr5*-DTR mice revealed a high density of *Lgr5*^*+*^ MIRFs (Fig. 3a–f), contrasting with their absence in unloaded regions (dorsal foot, back, ear, tail), as shown in *Lgr5*-tracing mice (Fig. 3a, b). Notably, DT-mediated ablation of plantar *Lgr5*^*+*^ cells induced fibrosis, inflammation, and disrupted epidermal architecture (Fig. 3c–f). Critically, reconstitution with *Lgr5*-overexpressing mouse dermal fibroblasts (MDFs) restored near-normal microstructure and reduced fibrotic inflammation, whereas control MDFs failed (Fig. 3c–f). We also focused on the process of wound healing, which captures acute, tension-dependent stress. In *Lgr5-*tracing mice, comparative analysis of wound models uncovered tension-dependent induction of *Lgr5*^*+*^ MIRFs: tension-bearing dorsal wounds exhibited *Lgr5*^*+*^ MIRF niches, whereas tension-neutral tail wounds remained devoid of this population, highlighting their mechanoresponsive behavior (Fig. 3g, h). Collectively, this tripartite mechanobiological framework delineates the niche specialization of *Lgr5*^*+*^ MIRFs across diverse mechanical contexts.

### YAP/AP-1 drives mechanoresponsive *LGR5* expression

**Fig. 4:**
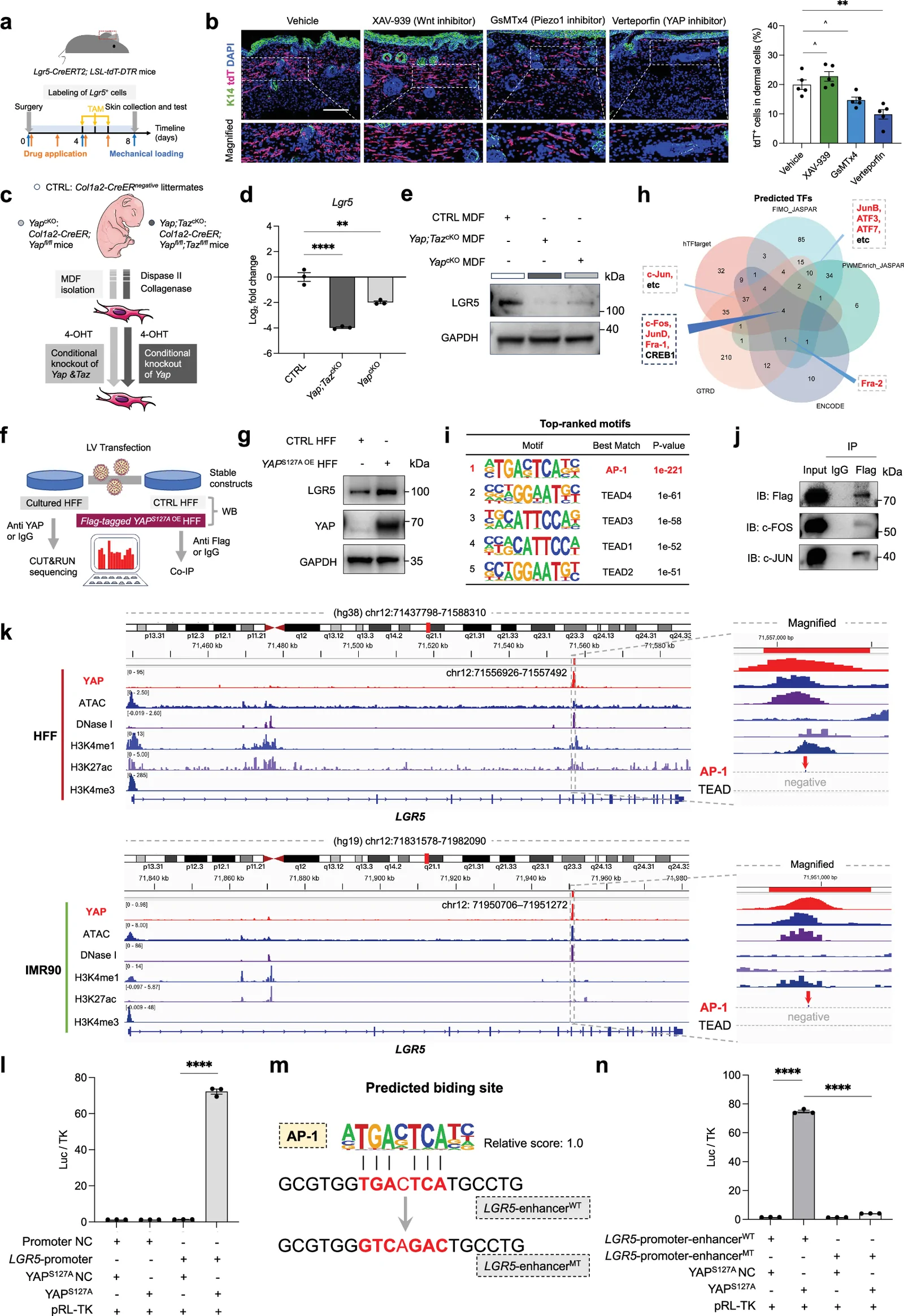
YAP/AP-1-dependent enhancer activation mediates mechanoresponsive *LGR5* expression. **a** Experimental timeline for local drug administration in *Lgr5*-tracing mice following skin expansion. **b** Immunofluorescence staining for K14 and tdT in expanded skin (*n* = 5 mice). Left, representative images (scale bar, 100 μm); right, quantification of tdT^+^ cells in dermal cells (^*P* = 0.5568 / 0.1076, **P = 0.0012). **c** Schematic workflow for the isolation and induction of *Yap*^cKO^ MDFs and *Yap;Taz*^cKO^ MDFs. Images provided by Servier Medical Art (https://smart.servier.com), licensed under CC BY 4.0 (https://creativecommons.org/licenses/by/4.0/). **d** qPCR analysis of *Lgr5* mRNA in control, *Yap;Taz*^cKO^ and *Yap*^cKO^ MDFs (*n* = 3 biological replicates) (**P = 0.0013, ****P < 0.0001). **e** Western blot of LGR5 in control, *Yap;Taz*^cKO^ and *Yap*^cKO^ MDFs. **f** Schematic workflow for CUT&RUN sequencing, generation of CTRL and *YAP*^S127A OE^ HFFs and Co-IP/WB analyses. Images provided by Servier Medical Art (https://smart.servier.com), licensed under CC BY 4.0 (https://creativecommons.org/licenses/by/4.0/). **g** Western blot of LGR5 in CTRL and *YAP*^S127A OE^ HFFs. **h** Venn diagram showing overlap of predicted transcription factors from different databases. **i** Motif enrichment analysis showing the top-ranked motifs identified from CUT&RUN sequencing with a YAP antibody in HFFs. **j** Co-IP analysis of Flag-tagged YAP^S127*A*^ with c-FOS and c-JUN. Lysates from *YAP*^S127A OE^ HFFs were subjected to IP using an anti-Flag antibody, followed by IB with antibodies against c-FOS, c-JUN and Flag. **k** Genome‑browser tracks displaying sequencing signals in HFFs (upper) and IMR‑90 cells (lower). Right panels show magnified views of the putative enhancer region with motif matches for AP‑1 and TEAD. **l** Dual-luciferase reporter assay in HEK293T measuring transcription activity of the *LGR5* promoter upon co‑transfection with *YAP*^*S127A*^ or negative controls (*n* = 3 technical replicates, representative of two independent experiments) (****P < 0.0001). **m** Schematic of the wild‑type (WT) and mutant (MT) constructs of the putative *LGR5* enhancer, with mutations introduced at the predicted AP‑1 binding site. **n** Dual-luciferase reporter assay in HEK293T measuring transcription activity of the *LGR5* promoter linked to either the WT or MT enhancer, in the presence or absence of *YAP*^*S127A*^ co‑transfection (*n* = 3 technical replicates, representative of two independent experiments) (****P < 0.0001). Data are presented as mean ± SEM. Statistical significance was determined by one-way ANOVA with Tukey’s post hoc test. Source data are provided as a Source Data file.

Next, we probed the pathway by which mechanical force induces *LGR5* expression. We first screened three major pathways implicated in mechanotransduction and LGR5 biology, including YAP, Piezo1, and Wnt signaling^37^. Pharmacological inhibition in *Lgr5*-tracing mice pointed to YAP activity, but not Piezo1 or canonical Wnt signaling, as a primary mediator of *Lgr5*^*+*^ MIRF induction (Fig. 4a, b). To genetically validate this link, we generated mice with fibroblast-specific knockout of both *Yap* and its homolog *Taz* (*Yap*^*fl/fl*^*;Taz*^*fl/fl*^*; Col1a2-CreER* mice, hereafter *Yap;Taz*^cKO^), as well as *Yap* single knockout (*Yap*^*fl/fl*^*; Col1a2-CreER* mice, hereafter *Yap*^cKO^) (Supplementary Fig. 4a). Primary MDFs from these mice exhibited a marked reduction of *Lgr5* expression upon induction, establishing *Yap*/*Taz* as critical upstream regulators (Fig. 4c–e and Supplementary Fig. 4b). The incomplete compensation by *Taz* in *Yap*^cKO^ cells indicated a dominant role for *Yap*. Functionally, loss of *Yap*/*Taz* or *Yap* impaired fibroblast-mediated fibrogenesis and diminished matrix contractility (Supplementary Fig. 4c, d). Consistent with in vitro findings, mechanical stretch failed to induce dermal *Lgr5* upregulation in *Yap*^cKO^ mice in vivo (Supplementary Fig. 4e–g). Moreover, *Yap;Taz*^cKO^ animals exhibited profound impairment in overall skin adaptation to stretch, underscoring the necessity of *Yap*/*Taz* activation for successful mechanoresponsiveness (Supplementary Fig. 4h–j). In primary human foreskin fibroblasts (HFFs), forced expression of constitutively active *YAP*^S127A^ also upregulated LGR5 expression (Fig. 4f, g).

As YAP requires DNA-binding partners to exert transcriptional control^38^, we sought its collaborator in regulating *LGR5* expression. Integrated analysis of public databases nominated components of the Activator Protein-1 (AP-1) complex, including FOS and JUN proteins, as top candidate regulators of human LGR5 (Fig. 4h). To pinpoint the genomic regulatory elements, we performed CUT&RUN (cleavage under targets and release using nuclease) sequencing in HFFs (Fig. 4f and Supplementary Fig. 4k–m). Motif analysis of YAP-bound regions ranked AP-1 as the most enriched (Fig. 4i). This was further confirmed by our co-immunoprecipitation showing robust YAP/c-FOS and YAP/c-JUN interactions in HFFs (Fig. 4j and Supplementary Fig. 5a, b). Examination of the *LGR5* locus pinpointed a distal enhancer as the primary YAP recruitment site (Fig. 4k), consistent with a recent study describing mechanoenhancers as mechanoresponsive cis-regulatory elements^39^. Notably, this enhancer contained a canonical AP-1 motif but lacked a motif for TEAD (Fig. 4k), YAP’s primary DNA-binding partner^40^. This distinctive configuration was conserved in public YAP ChIP-seq data from IMR90 (a cell line of human embryonic lung fibroblast), as well as two other YAP-hyperactive cell lines (NCI-H2052 and SF268) (Fig. 4k and Supplementary Fig. 4m). Dual-luciferase reporter assays demonstrated that transfection of the YAP^S127A^ plasmid in HEK293T cells potently activated the *LGR5* promoter (Fig. 4l). This activation was dependent on LGR5 enhancer and was significantly attenuated upon mutation of the AP-1 binding site (Fig. 4m, n). We specifically validated the role of c-FOS, a core AP-1 component supported by literature evidence as a critical YAP partner^41,42^. *FOS* overexpression in HFFs was sufficient to upregulate *LGR5* and enhance matrix deposition (Supplementary Fig. 5c, d). Conversely, genetic ablation of *cFos* (*cFos*^fl/fl^; *Col1a2-CreER* mice, hereafter *cFos*^cKO^) in MDFs reduced *Lgr5* expression in MDFs (Supplementary Fig. 5e–h) and recapitulated the *Yap*;*Taz*^cKO^ phenotype, attenuating pro-fibrotic matrix deposition and contraction (Supplementary Fig. 5h, i).

In summary, these data delineate a transcriptional cascade where mechanical force activates YAP, which then partners with AP-1 at a specific distal enhancer to drive *LGR5* expression, defining the molecular core of the mechanoresponsive fibroblast program.

### *Lgr5* loss compromises mechanoadaptive tissue remodeling

**Fig. 5:**
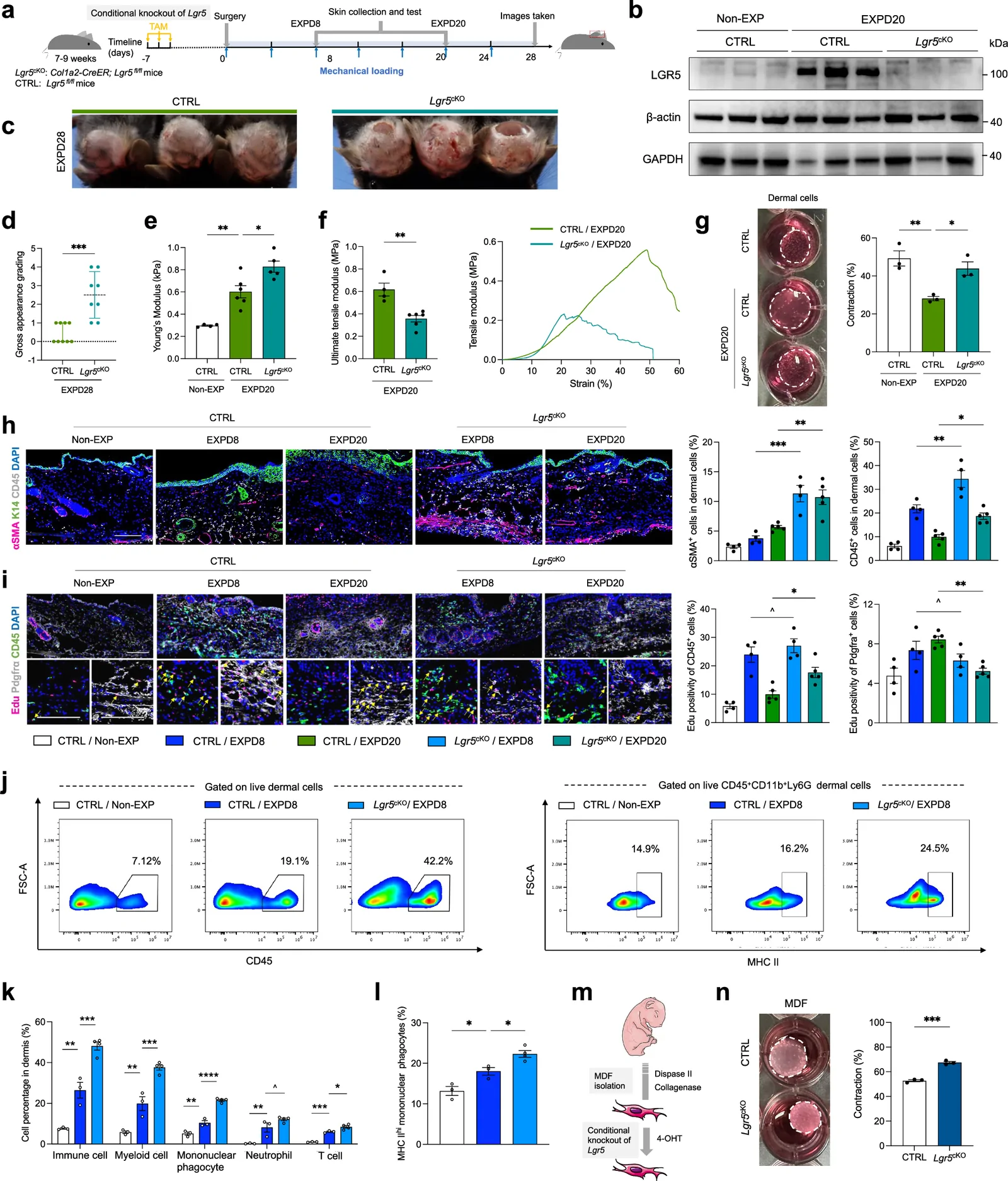
Conditional *Lgr5* knockout disrupts skin mechanoadaptation and promotes fibro-inflammatory remodeling. **a** Schematic timeline for assessing mechanoadaptation in *Lgr5*^cKO^ mice and their littermate controls (CTRL) following skin expansion. **b** Western blot of LGR5 in non-expanded skin and expanded skin of CTRL versus *Lgr5*^*cK*O^ mice (*n* = 3 mice). **c** Representative images of the expanded region. **d** Quantification of appearance scoring (*n* = 8–9 mice) (***P = 0.0006). **e** Quantification of tissue stiffness (*n* = 4–6 mice) (*P = 0.0126, **P = 0.0025). **f** Biomechanical assessment of tensile strength (*n* = 4–6 mice). Left, quantification (**P = 0.0022); right, representative curves. **g** Matrix contraction assay (*n* = 3 mice). Top, representative images; bottom, quantification of contraction percentage (**P = 0.0077, *P = 0.0284). **h** Immunofluorescence staining for K14, αSMA and CD45 (*n* = 4–5 mice). Left, representative images (scale bar, 100 μm); right, quantification of αSMA^+^ cells (**P = 0.0039, ***P = 0.0002) and CD45^+^ cells (*P = 0.0169, **P = 0.0020). **i** Immunofluorescence staining for EdU, CD45 and Pdgfra (*n* = 4–5 mice). Left, representative images (scale bar, 100 μm); right, quantification of EdU positivity of CD45^+^ cells (^*P* = 0.7993, *P = 0.0464) and Pdgfra^+^ cells (^*P* = 0.7675, **P = 0.0071). **j** Gating strategies and representative plots from comprehensive immune profiling. **k** Quantification of distinct cell populations (*n* = 3–4 mice) (^*P* = 0.1243, *P = 0.0220, **P = 0.0032 / 0.0054 / 0.0061 / 0.0093, ***P = 0.0009 / 0.0009 / 0.0006, ****P < 0.0001). **l** Quantification of MHC II^hi^ cells among mononuclear phagocytes (*n* = 3–4 mice) (*P = 0.0258 / 0.0335). **m** Schematic workflow for the isolation and induction of *Lgr5*^cKO^ MDFs. Images provided by Servier Medical Art (https://smart.servier.com), licensed under CC BY 4.0 (https://creativecommons.org/licenses/by/4.0/). **n** Matrix contraction assay (*n* = 3 biological replicates). Left, representative images; right, quantification of contraction percentage (***P = 0.0003). Data are presented as mean ± SEM for all panels except (**d**). For dataset (**d**), data are shown as scatter plots with median and interquartile range, and statistical significance was determined by the two-tailed Mann–Whitney test. Otherwise, statistical significance was determined by one-way ANOVA with Tukey’s post hoc test or two-tailed unpaired Student’s *t*-test. Source data are provided as a Source Data file.

To dissect the functional necessity of LGR5 in skin mechanoadaptation, we generated fibroblast-specific conditional *Lgr5* knockout mice (*Lgr5*^fl/fl^; *Col1a2-CreER* mice, hereafter *Lgr5*^cKO^) (Supplementary Fig. 6a). Western blot confirmed LGR5 depletion in expanded skin of *Lgr5*^cKO^ mice (Fig. 5a, b). Strikingly, under repetitive mechanical loading, *Lgr5*^cKO^ skin ruptured prematurely, while littermate controls maintained structural integrity (Fig. 5c). Consistently, *Lgr5*^cKO^ mice displayed defective adaptive remodeling during skin expansion, as reflected by increased tissue stiffness, reduced ultimate tensile strength, and hyperactive matrix contractility (Fig. 5d–g). Histological and cellular analysis revealed regenerative impairments in both dermal and epidermal compartments of LGR5-deficient skin. The dermis showed pronounced fibrosis and inflammatory infiltration, marked by elevated myofibroblast and immune cell accumulation, excessive matrix deposition, and mildly diminished fibroblast proliferation (Fig. 5h, i and Supplementary Fig. 6b). The epidermis displayed thinning, alongside impaired basal cell proliferation and differentiation (Supplementary Fig. 6c, d). Immune profiling further demonstrated increased CD45^+^ cell infiltration in *Lgr5*^cKO^ skin, primarily due to expanded myeloid-lineage cells, particularly mononuclear phagocytes (Fig. 5j, k). Among these phagocytes, the proportion of MHC II ^hi^ macrophages was significantly elevated, indicative of heightened immune activation (Fig. 5j, l). In vitro assay using primary murine dermal fibroblasts (MDFs) recapitulated the increased matrix contractility (Fig. 5m, n), suggesting a cell-autonomous function of LGR5 in fibroblasts.

To validate the mechanical dependence of LGR5 function, we extended our analyses to two complementary models. In the chronic pressure model, *Lgr5* deletion selectively impaired full-skin adaptive remodeling in mechanically stressed plantar skin, while skin from unloaded regions showed no discernible phenotypic differences (Supplementary Fig. 6e–h). During dorsal wound healing—a process accompanied by intrinsic mechanical tension—*Lgr5* loss did not affect initial wound closure but significantly delayed late-stage tissue remodeling and exacerbated fibrosis (Supplementary Fig. 6i–m). By contrast, tail wounds, which heal in a tension-sparing microenvironment, showed normal healing kinetics in *Lgr5*^cKO^ mice (Supplementary Fig. 6i–k). These data underscore the context-dependent role of LGR5 in mechanical stress-responsive tissue remodeling.

Collectively, our results identify dermal LGR5 as a critical regulator of skin mechanoadaptation, which maintains tissue plasticity and prevents maladaptive fibro-inflammatory remodeling in response to mechanical stress.

### Conditional *Lgr5* upregulation drives skin mechanoadaptation

**Fig. 6:**
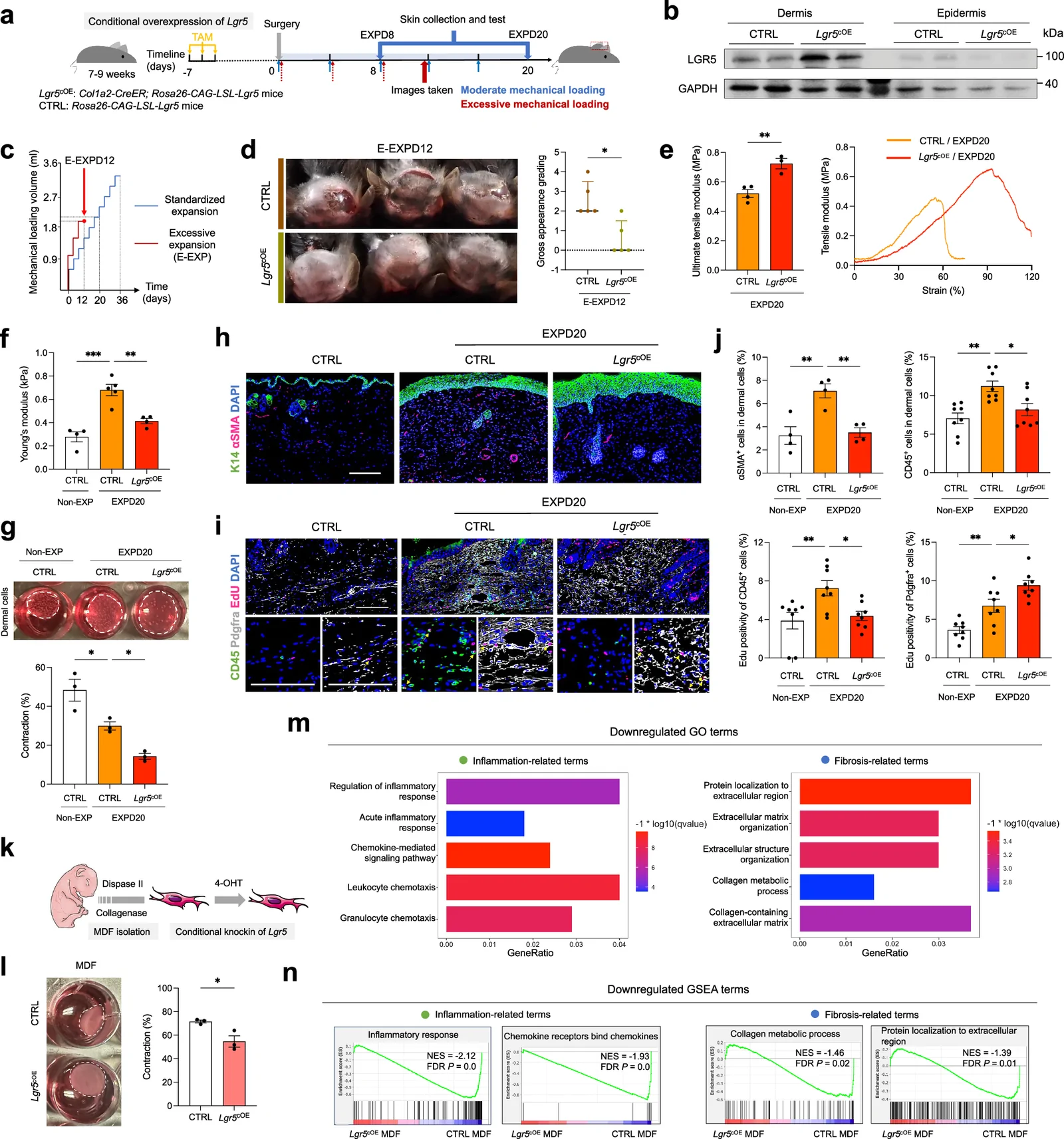
*Lgr5* overexpression in fibroblasts enhances tissue resilience and restrains fibro-inflammatory responses. **a** Schematic timeline for assessing mechanoadaptation in *Lgr5*^cOE^ mice and their littermate controls (CTRL) following skin expansion. Blue arrows indicate standardized expansion (**e–j**), while red arrows indicate excessive expansion (**c, d**). **b** Western blot of LGR5 in dermis and epidermis of non-expanded skin of CTRL versus *Lgr5*^*cOE*^ mice. **c** Mechanical loading protocol comparing excessive expansion (E-EXP) with standardized expansion. **d** Macroscopic evaluation (*n* = 5 mice). Left, representative images of the expanded region; right, quantification of appearance scoring (*P = 0.0317). **e** Biomechanical assessment of tensile strength (*n* = 3–4 mice). Left, quantification (**P = 0.0055); right, representative curves. **f** Quantification of tissue stiffness (*n* = 4–5 mice) (**P = 0.0027, ***P = 0.0001). **g** Matrix contraction assay (*n* = 3 mice). Top, representative images; bottom, quantification of contraction percentage (*P = 0.0255/0.0485). **h** Immunofluorescence staining for K14 and αSMA (scale bar, 100 μm). **i** Immunofluorescence staining for EdU, CD45 and Pdgfra (scale bar, 100 μm). **j** Quantification of αSMA^+^ cells (**P = 0.0039 / 0.0061) (*n* = 4 mice), and of CD45^+^ cells (*P = 0.0206, **P = 0.0016), and EdU positivity of CD45^+^ cells (*P = 0.0274, **P = 0.0094) and Pdgfra^+^ cells (*P = 0.0261, **P = 0.0079) (*n* = 8 mice). **k** Schematic workflow for the isolation and induction of *Lgr5*^cOE^ MDFs. Images provided by Servier Medical Art (https://smart.servier.com), licensed under CC BY 4.0 (https://creativecommons.org/licenses/by/4.0/). **l** Matrix contraction assay (*n* = 3 biological replicates). Left, representative images; right, quantification of contraction percentage (*P = 0.0264). **m** Bar plots showing the significantly downregulated GO terms associated with inflammation (left) and fibrosis (right) in *Lgr5*^cOE^ MDFs. **n** GSEA plots showing the significantly downregulated gene sets for inflammation (left) and fibrosis (right) in *Lgr5*^cOE^ MDFs. Data are presented as mean ± SEM for all panels except (**d**). For dataset (**d**), data are shown as scatter plots with median and interquartile range, and statistical significance was determined by the two-tailed Mann-Whitney test. Otherwise, statistical significance was determined by one-way ANOVA with Tukey’s post hoc test or two-tailed unpaired Student’s *t*-test. Source data are provided as a Source Data file.

We next asked whether *Lgr5* overexpression is sufficient to promote skin mechanoadaptation using fibroblast-specific conditional *Lgr5* overexpression mice (*Rosa26-LSL-CAG-Lgr5; Col1a2-CreER* mice, hereafter *Lgr5*^cOE^) (Supplementary Fig. 7a). Western blot verified LGR5 overexpression in the dermis of *Lgr5*^cOE^ mice (Fig. 6a, b). Under excessive mechanical loading, *Lgr5*^cOE^ skin exhibited markedly improved resistance to mechanical failure compared with littermate controls (Fig. 6c, d). Correspondingly, *Lgr5*^cOE^ animals exhibited superior biomechanical properties during standardized expansion, including decreased tissue stiffness, increased ultimate tensile strength, and reduced matrix contractility (Fig. 6e–g). Histologically, *Lgr5*^cOE^ skin showed thickened dermis and attenuated fibro-inflammatory responses, characterized by reduced myofibroblast accumulation, diminished immune infiltration, and moderately enhanced fibroblast proliferation (Fig. 6h–j and Supplementary Fig. 7b, c). The epidermis of *Lgr5*^cOE^ mice showed thickening due to enhanced keratinocyte proliferation and differentiation (Supplementary Fig. 7b–e). These phenotypes were recapitulated in the chronic pressure and wound-healing models. *Lgr5* overexpression specifically enhanced adaptive structure formation in mechanically loaded plantar skin but not in unloaded regions (Supplementary Fig. 7f–i). During dorsal wound healing, *Lgr5*^cOE^ accelerated late-stage healing and improved regenerative remodeling, mirroring wound‑induced hair follicle neogenesis (WIHN) that favors a softened environment (Supplementary Fig. 7j–n), while tail skin healing—independent of mechanical tension—remained unaffected (Supplementary Fig. 7j–l).

To confirm these pro-mechanoadaptive functions in vitro, we conducted complementary experiments using primary MDFs overexpressing *Lgr5* (*Lgr5*^cOE^ MDFs). *Lgr5*^cOE^ MDFs displayed a transcriptome skewed toward anti-inflammatory and anti-fibrotic programs, coupled with reduced matrix contraction (Fig. 6k–n and Supplementary Fig. 8a). Transwell chemotaxis assays confirmed that *Lgr5*^cOE^ MDFs suppressed immune cell recruitment in vitro (Supplementary Fig. 8b). Additionally, *Lgr5*^cOE^ MDFs exhibited a proliferation advantage (Supplementary Fig. 8c). To assess evolutionary conservation, we generated HFFs stably overexpressing LGR5 (*LGR5*^OE^ HFFs). These cells displayed conserved anti-fibrotic and anti-inflammatory signatures (Supplementary Fig. 8d–g), accompanied by profound alterations in major ECM components and their regulatory factors (Supplementary Fig. 8h, i), suggesting enhanced matrix remodeling and a shift toward reduced matrix density. Functional assays validated increased proliferation and reduced matrix contraction (Supplementary Fig. 8j–l). Interestingly, *LGR5* overexpression effectively counteracted the pro-fibrotic and matrix-contracting effects induced by the constitutively active *YAP*^S127A^ (Supplementary Fig. 8m–o). Together, these data demonstrate the evolutionarily conserved function of LGR5 in mediating mechanoadaptive tissue remodeling.

### LGR5 inhibits JAK-STAT activity via JAK1 interaction

**Fig. 7:**
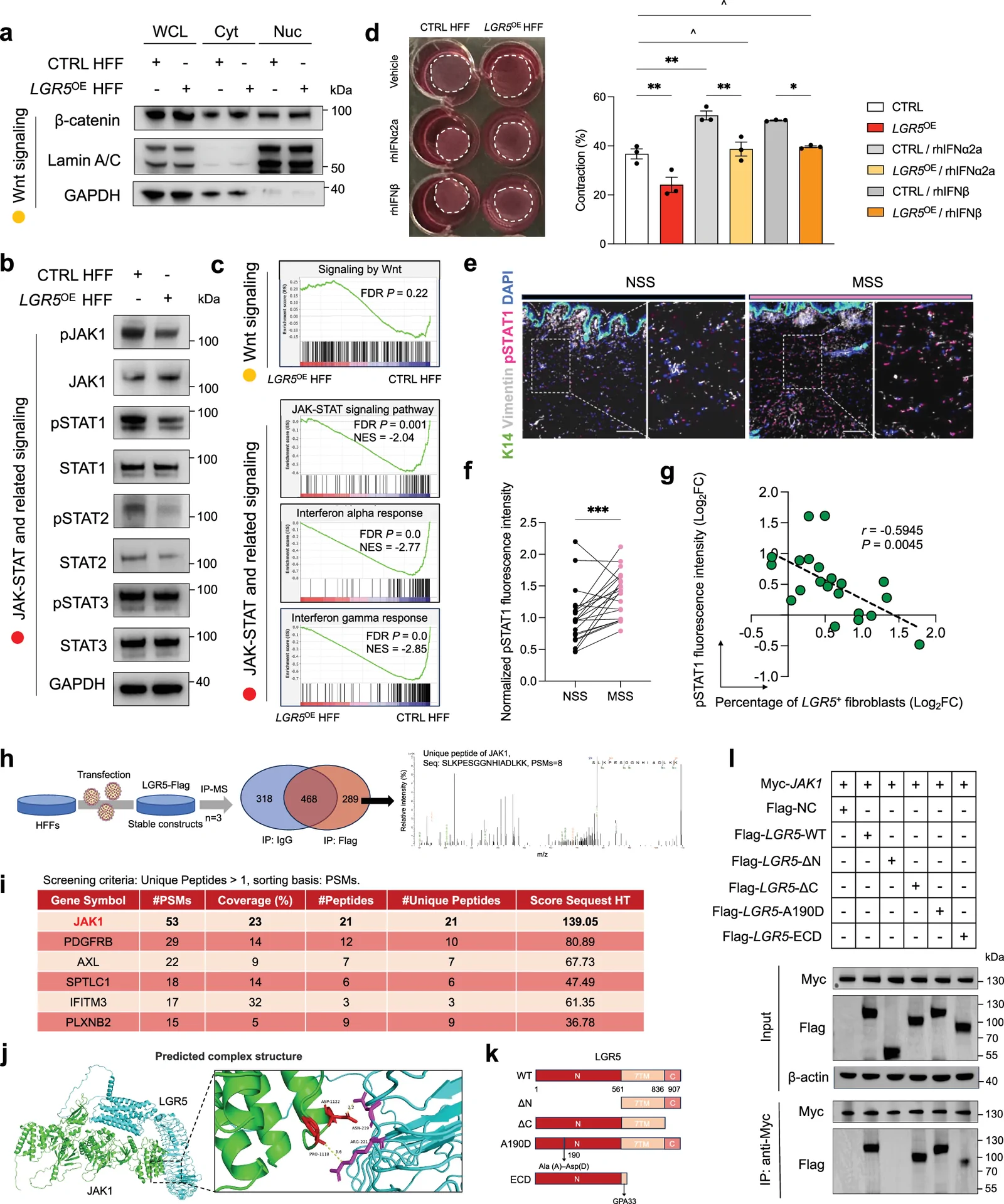
LGR5 antagonizes JAK-STAT signaling through interaction with JAK1. **a** Western blot of β-catenin expression and distribution in whole cell lysates (WCL), cytoplasm (Cyt) and nucleus (Nuc) fractions in control (CTRL) and *LGR5*^OE^ HFFs. GAPDH and Lamin A/C served as loading controls. **b** Western blot of JAK-STAT pathway in CTRL and *LGR5*^OE^ HFFs. **c** GSEA plots showing enrichment of Wnt signaling, JAK-STAT signaling, and related interferon signaling gene sets in *LGR5*^cOE^ HFFs. **d** Matrix contraction assay mediated by *LGR5*^OE^ or CTRL HFFs, treated with or without rhIFNα2a and rhIFNβ (*n* = 3 biological replicates). Left, representative images; right, quantification of contraction percentage (^*P* = 0.9809 / 0.9114, *P = 0.0271, **P = 0.0016 / 0.0093 / 0.0049). **e** Representative immunofluorescence images of Vimentin, K14 and pSTAT1 in the indicated human skin samples (scale bar, 100 μm). **f** Quantification of normalized pSTAT1 fluorescence intensity in pairs (*n* = 21 pairs) (***P = 0.0008). **g** Correlation between log_2_ fold change (MSS/NSS) in dermal pSTAT1 fluorescence intensity and the percentage of *LGR5*^*+*^ fibroblasts (*n* = 21 pairs). **h** Schematic workflow for IP-MS in Flag-tagged *LGR5*^OE^ HFFs, identifying JAK1 as a putative protein binding to LGR5. Images provided by Servier Medical Art (https://smart.servier.com), licensed under CC BY 4.0 (https://creativecommons.org/licenses/by/4.0/). **i** Table of top-enriched proteins identified by IP-MS. **j** Predicted structure of the LGR5-JAK1 protein complex. **k** Diagrams of the Flag‑tagged full‑length and truncated/mutated versions of LGR5. **l** Co‑IP of JAK1 and different Flag‑LGR5 constructs. Lysates from HFF co‑expressing Myc‑JAK1 and different Flag‑LGR5 constructs were immunoprecipitated with anti‑Myc antibody and immunoblotted as indicated. Data are presented as mean ± SEM. Statistical significance was determined by one-way ANOVA with Tukey’s post hoc test (**d**), two-tailed paired Student’s *t*-test (**f**), and two-tailed Pearson correlation analysis (**g**). Source data are provided as a Source Data file.

To identify the signaling pathways through which LGR5 regulates fibroblast function, we performed Western blot analysis and global transcriptomic profiling in *LGR5*^OE^ HFFs (Fig. 7a–c). Surprisingly, Wnt signaling, the canonical LGR5-dependent signaling in epithelial cells^43^, remained largely unchanged (Fig. 7a, c). Notably, JAK-STAT signaling, a pathway widely hyperactivated in inflammatory and fibrotic disorders^44,45^, was robustly suppressed in *LGR5*^OE^ HFFs, marked by pronounced inhibition of STAT1 and STAT2 phosphorylation, with minimal effect on STAT3 activation (Fig. 7b, c). Corresponding analyses in primary murine dermal fibroblasts (MDFs) isolated from *Lgr5*^cOE^ and *Lgr5*^cKO^ mice confirmed this trend: *Lgr5* overexpression repressed JAK-STAT activity, whereas *Lgr5* ablation augmented it, with no detectable changes in Wnt signaling output in either genotype (Supplementary Fig. 9a–e). To functionally validate this regulatory axis, we assessed the responsiveness of JAK-STAT signaling to interferon (IFN) stimulation. IFN-induced JAK-STAT activation was significantly attenuated in *LGR5*^OE^ HFFs or *Lgr5*^cOE^ MDFs, compared to control cells (Supplementary Fig. 9f–h). Collagen contraction assays further demonstrated that IFN-induced JAK-STAT activation reversed the attenuation of matrix contractility observed in *LGR5*^OE^ HFFs or *Lgr5*^cOE^ MDFs (Fig. 7d and Supplementary Fig. 9i). Conversely, the excessive matrix contractility of *Lgr5*^cKO^ MDFs was rescued by treatment with the JAK inhibitor ruxolitinib (Supplementary Fig. 9j). The physiological relevance of this regulation was underscored in vivo: mechanical stretching induced STAT1 phosphorylation in mouse skin, which was blunted upon *Lgr5* overexpression in fibroblasts (Supplementary Fig. 9k). Critically, we extended these findings to human tissue samples, as MSS dermis exhibited robust pSTAT1 activation compared to NSS, and the relative level of pSTAT1 was inversely correlated with the relative abundance of LGR5^+^ fibroblasts (Fig. 7e–g). This observation directly links LGR5 expression to JAK-STAT modulation in the human skin mechanoresponse.

To delineate the molecular mechanism by which LGR5 inhibits the JAK–STAT pathway, we conducted Flag-based immunoprecipitation coupled with mass spectrometry (IP-MS) in *LGR5*^OE^ HFFs. Interestingly, this screen identified Janus kinase 1 (JAK1) as a top interactor of LGR5 (Fig. 7h, i, and Supplementary Data 1). Protein structure modeling further suggested that the N-terminal domain of LGR5 engages the kinase domain of JAK1, with the predicted binding interface spatially proximal to the known binding site for its canonical ligand R-spondin1^46,47^(Fig. 7j). We confirmed this direct LGR5-JAK1 interaction by co-immunoprecipitation. Notably, an N-terminal truncation (ΔN) of LGR5 completely abolished binding to JAK1 (Fig. 7k, l). By contrast, LGR5-JAK1 interaction was preserved in three LGR5 variants: a C-terminal truncation (ΔC), a chimeric construct containing the LGR5 ectodomain (ECD), and a point mutant (A190D) at a key N-terminal residue required for R-spondin1 engagement^46^(Fig. 7k, l). Consistent with the prediction of spatially adjacent but distinct binding interfaces, treatment with R-spondin1 reversed LGR5-mediated JAK-STAT inhibition, but this restorative effect was attenuated in *LGR5*^OE^ cells relative to controls (Supplementary Fig. 9l). These results establish that LGR5 preferentially targets JAK1 via its N-terminal domain to mediate inhibition in fibroblasts, with this interaction potentially involving competition with the canonical ligand R-spondin1.

Together, these findings define a non-canonical mechanism whereby LGR5 interacts with JAK1 to negatively regulate JAK-STAT activity, with a preferential impact on the STAT1/2 branch, thereby fine-tuning fibroblast mechanoresponsiveness.

### JAK1 inhibiton mimics LGR5^*+*^ MIRF-driven regeneration

**Fig. 8:**
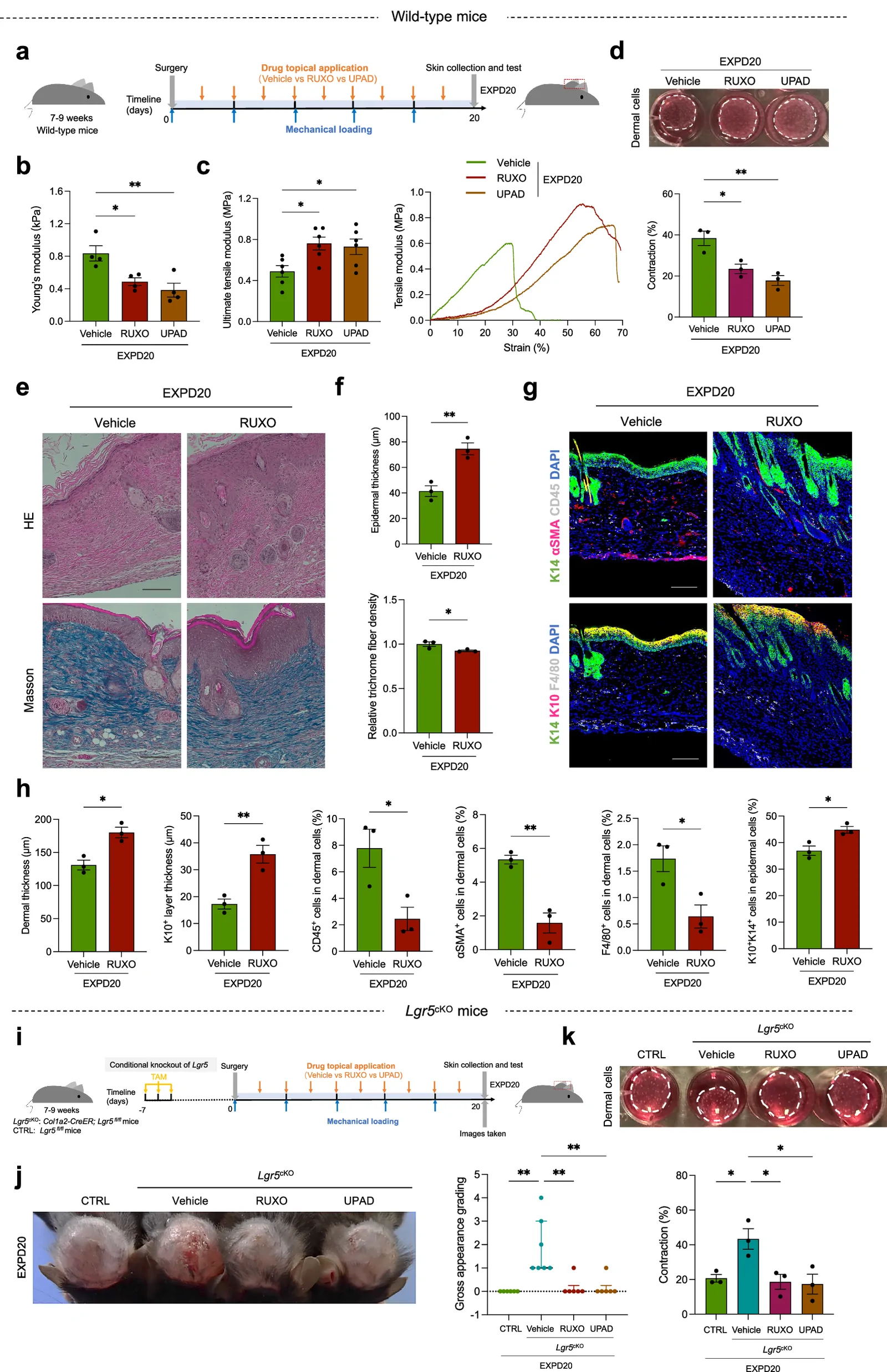
Pharmacological JAK1 inhibition recapitulates LGR5-mediated regenerative advantage. **a** Schematic timeline for assessing mechanoadaptation in wild-type mice following skin expansion, treated topically with vehicle, ruxolitinib (RUXO) or upadacitinib (UPAD). **b** Quantification of tissue stiffness (*n* = 4 mice) (*P = 0.0285, **P = 0.0070). **c** Biomechanical assessment of tensile strength (*n* = 6 mice). Left, quantification (*P = 0.0492/0.0253); right, representative curves. **d** Matrix contraction assay (*n* = 3 mice). Top, representative images; bottom, quantification of contraction percentage (*P = 0.0213, **P = 0.0047). **e** Representative images of the expanded skin of indicated mice stained with H&E staining and Masson’s trichrome staining (scale bar, 100 μm). **f** Quantification of epidermal thickness (**P = 0.0062) and relative trichrome density (*P = 0.0495) (*n* = 3 mice). **g **Immunofluorescence analysis of expanded skin of indicated mice. Upper panels, representative images of K14, αSMA and CD45; lower panels, representative images of K14, K10 and F4/80; scale bar, 100 μm. **h** Quantification of the following parameters (*n* = 3 mice): dermal thickness (*P = 0.0106); K10^+^ layer thickness (**P = 0.0081); the percentage of CD45^+^ cells (*P = 0.0342), αSMA^+^ cells (**P = 0.0043), and F4/80^+^ cells (*P = 0.0287); and the percentage of K10^+^K14^+^ cells (*P = 0.0221). **i** Schematic timeline for assessing mechanoadaptation in *Lgr5*^cKO^ mice following skin expansion, treated topically with vehicle, RUXO or UPAD, alongside littermate controls. **j** Macroscopic evaluation (*n* = 6–7 mice). Left, representative images of the expanded region; right, quantification of appearance scoring (**P = 0.0088/0.0013/0.0088). **k** Matrix contraction assay (*n* = 3 mice). Top, representative images; bottom, quantification of contraction percentage (*P = 0.0202/0.0400/0.0265). Data are presented as mean ± SEM for all panels except (**j**). For dataset (**j**), data are shown as scatter plots with median and interquartile range, and statistical significance was determined by the Kruskal–Wallis test followed by Dunn’s multiple comparisons test. Otherwise, statistical significance was determined by a two-tailed unpaired Student’s *t*-test or one-way ANOVA with Tukey’s post hoc test. Source data are provided as a Source Data file.

Given the central role of JAK-STAT suppression in LGR5-mediated mechanoadaptation, we asked whether pharmacological JAK inhibition could phenocopy the regenerative advantage of LGR5. To test this, we topically applied either the non-selective JAK1/2 inhibitor ruxolitinib or the selective JAK1 inhibitor upadacitinib to wild-type mice undergoing mechanical stretch (Fig. 8a). Relative to vehicle controls, both treatments significantly enhanced tissue resilience, increasing ultimate tensile strength while reducing tissue stiffness and matrix contractility of dermal cells (Fig. 8b–d). H&E and Masson’s trichrome staining further revealed a thickened dermal-epidermal architecture and reduced excessive collagen deposition (Fig. 8e, f and Supplementary Fig. 10a, b). Immunofluorescence analysis confirmed reduced accumulation of αSMA^+^ myofibroblasts and reduced CD45^+^ immune infiltration, concomitant with enhanced epidermal basal cell proliferation and differentiation, indicating attenuated fibro-inflammatory responses and improved regenerative output (Fig. 8g, h and Supplementary Fig. 10c, d).

We next tested whether JAK inhibition could rescue the mechanoadaptive deficits in *Lgr5*^cKO^ mice. Topical administration of ruxolitinib or upadacitinib during skin expansion substantially ameliorated the premature rupture phenotype and restored mechanical performance in *Lgr5*^cKO^ mice (Fig. 8i–k). Histological and cellular analyses further demonstrated restored dermal stromal organization, reduced fibro-inflammatory responses, and recovery of epidermal homeostasis in treated *Lgr5*^cKO^ mice (Supplementary Fig. 10e–h). These data confirm that JAK1 inhibition can functionally compensate for *Lgr5* deficiency in vivo.

Collectively, these data establish JAK1 as a therapeutically actionable node downstream of LGR5 and demonstrate that topical JAK inhibitors emulate the pro-regenerative, anti-fibrotic effects of LGR5^*+*^ MIRFs in mechanically challenged skin.

## Discussion

Here, we identify LGR5^*+*^ MIRFs as a critical mechanoregulatory brake that channels diverse mechanical stress into a unified regenerative response. This finding prompts a conceptual shift in stromal biology: whereas fibroblasts are typically viewed as effectors that promote fibro-inflammatory responses^14,16,23,48,49^, LGR5^*+*^ MIRFs restrain these maladaptive processes while promoting balanced matrix remodeling, thereby enabling functional mechanoadaptation.

The core molecular logic of this brake is a YAP-to-LGR5 negative-feedback axis. We demonstrate that YAP/TAZ‑driven matrix deposition is necessary to launch adaptive remodeling; however, its persistent overactivation, frequently associated with poor mechanical outcomes, can propagate a cycle of tissue fibrosis, scarring and stiffening^15,50–52^. Here we identify a self‑limiting system in which mechanical force activates YAP, which then partners with AP-1 at a distal enhancer to drive LGR5 transcription. In turn, LGR5 expression curbs fibrogenesis and tissue contraction, directly opposing the matrix-producing and matrix-contracting roles of YAP/TAZ and AP-1. Mechanistically, LGR5 engages JAK1 through a non-canonical interaction to suppress JAK-STAT signaling, tempering the fibro-inflammatory outputs of sustained mechanoactivation. While the JAK-STAT pathway is well-established in inflammatory signaling^44^, growing evidence also implicates it in fibrotic processes across tissues such as bone marrow^53,54^, liver^55,56^, heart^57^, and lung^58^. Our study now provides a distinct mechanobiological perspective: we position the LGR5-JAK1 interaction as a dedicated braking mechanism that is induced by mechanical stress to prevent the default fibro-inflammatory trajectory typically associated with chronic mechanoactivation.

This axis establishes a self-limiting circuit that fine-tunes the remodeling balance, permitting necessary matrix adaptation while preventing its pathological overdrive. We propose that the balance between adaptive and maladaptive mechanotransduction is dictated by stress intensity: moderate loads engage LGR5 as a protective brake, whereas excessive or chronic stress overwhelms this circuit, leading to pathological fibrosis and even macroscopic skin damage. Thus, LGR5 induction may represent a compensatory response with finite protective capacity that can be exhausted under extreme loading.

The relevance of this LGR5-mediated regulatory circuit extends beyond our models. Its physiological importance is highlighted by its presence in the healthy human dermal reticular layer, as well as its documented reduction and signaling dysregulation in scleroderma^59^, a fibrotic skin disorder characterized by aberrant mechanotransduction^60^. Our findings provide a mechanistic basis for this pathology, as LGR5 loss leads to fibro-inflammatory dysregulation. Beyond skin, recent studies have localized LGR5 to mesenchymal niches of the lung^61^, intestine^62^, and prostate^63,64^. These observations collectively point to a conserved role for LGR5⁺ stromal cells in supporting epithelial maintenance. Notably, our data extend this concept by showing that under mechanical stress, LGR5⁺ MIRFs contribute to epithelial adaptive regeneration. They likely achieve this by fostering a permissive niche characterized by a softened matrix and reduced inflammatory infiltration, conditions known to support epithelial growth^65–67^. Considering these cross-organ parallels and pathological clues, LGR5^*+*^ stromal cells may act as conserved facilitators of integrated stromal‑epithelial co‑adaptation in mechanically challenged tissues.

LGR5⁺ MIRFs define a mechanically induced state that, once established, exhibits relative stability, even after load removal in certain cases. Their persistence in mechanically compromised skin thus appears paradoxical. This is resolved by understanding their role not in immediately counteracting physical ECM disruption and mechanical properties impairment, but in orchestrating the subsequent repair program: constraining maladaptive fibro-inflammation and steering the deposition of functional matrix to restore long-term mechanical properties. This model explains why their ablation locks tissue in a mechanically damaged state, whereas their overexpression accelerates regenerative remodeling and improves biomechanical outcomes. Thus, LGR5⁺ MIRFs orchestrate a transition from injury toward functional mechanoadaptation and long‑term tissue resilience.

The functional criticality of this LGR5-JAK1 axis underscores its translational potential. Since topical JAK inhibitors are clinically available and effectively recapitulate the regenerative advantage mediated by LGR5⁺ MIRFs in vivo^68^, they offer a practical mechanotherapeutic strategy to optimize tissue expansion outcomes, mitigate fibrosis or striae, and enhance regenerative remodeling in chronic wounds.

**Fig. 9:**
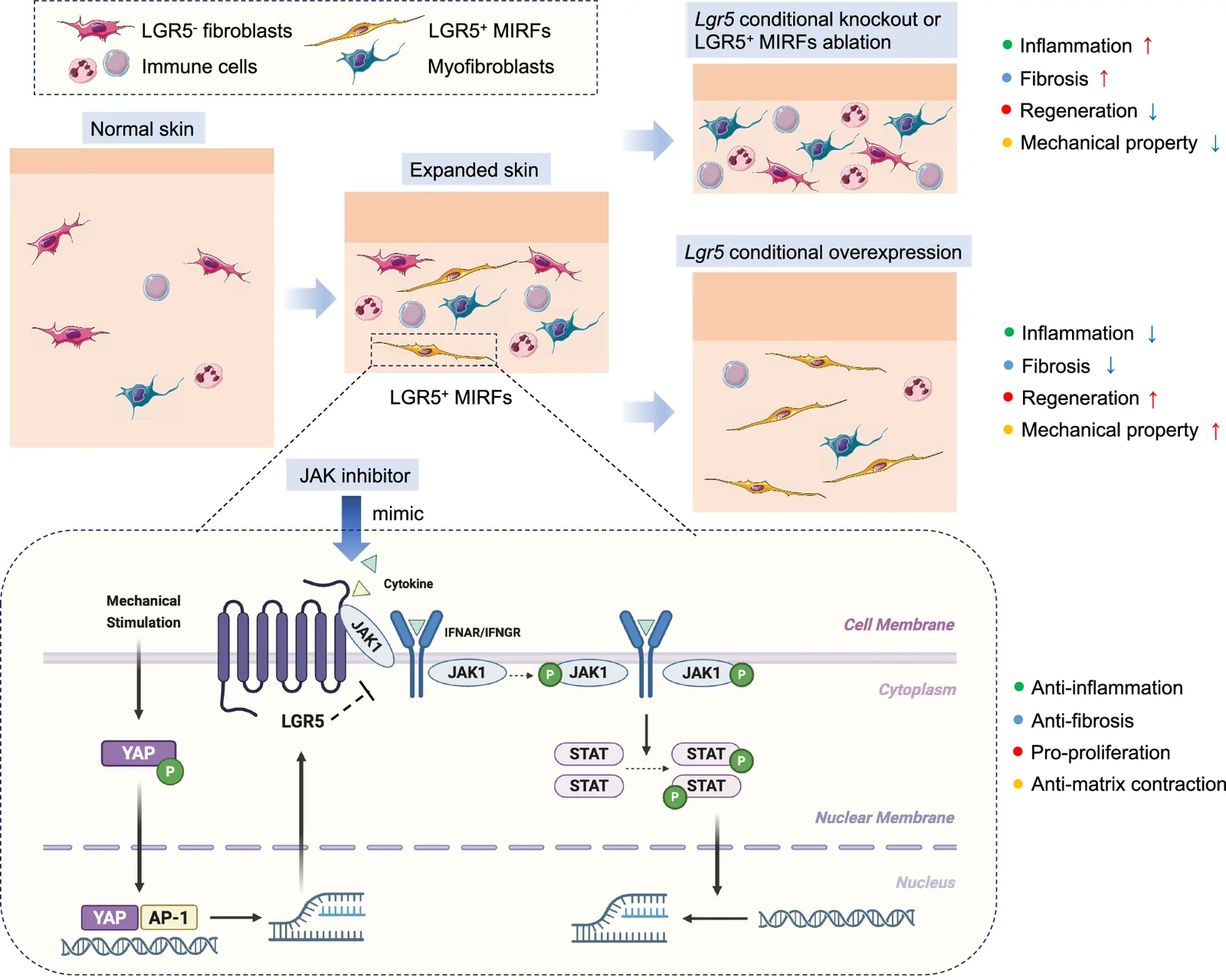
Proposed working model of LGR5-mediated skin mechanoadaptation. Mechanical stretch activates a YAP/AP-1-dependent transcriptional program that induces LGR5 expression in dermal fibroblasts, giving rise to LGR5^+^ mechanically induced regulatory fibroblasts (LGR5^+^ MIRFs). LGR5^+^ MIRFs promote adaptive tissue remodeling by suppressing JAK–STAT signaling, thereby limiting inflammation and fibrosis while enhancing tissue regeneration and mechanical extensibility. Conversely, depletion of LGR5^+^ MIRFs or fibroblast-specific deletion of *Lgr5* impairs mechanoadaptation, whereas fibroblast-specific *Lgr5* overexpression or topical JAK1 inhibition promotes adaptive remodeling. Created in BioRender. Cheng, X. (2026) https://BioRender.com/2633jlq. Images provided by Servier Medical Art (https://smart.servier.com), licensed under CC BY 4.0 (https://creativecommons.org/licenses/by/4.0/).

Beyond its canonical role as an epithelial stem cell marker, our work redefines LGR5 as a dedicated stromal mechanoresponder that couples extracellular mechanics to intracellular kinase regulation (Fig.9). By unveiling the LGR5^*+*^ MIRF-driven braking circuit, our work provides a new conceptual framework for understanding how tissues achieve plasticity under mechanical stress, with broad clinical implications for guiding mechanical adaptation toward functional regeneration.

## Methods

### Ethics statement

All experiments complied with relevant ethical regulations. Animal experiments were approved by the Institutional Animal Care and Use Committee (IACUC) of the Shanghai Institute of Nutrition and Health, Chinese Academy of Sciences (Approval No. SINH- 2020-ZL-1). Human studies were approved by the Ethical Review Board of Shanghai Ninth People’s Hospital, Shanghai Jiao Tong University School of Medicine (Approval No. SH9H-2025-T124-3 and No. 2020-2018-129-T107-1).

### Animals

All mice were maintained under specific-pathogen-free (SPF) conditions with a 12-h light/12-h dark cycle (temperature: 18–23 °C; humidity: 40–60%) and fed a standard rodent diet. Mice between 7 and 12 weeks of age were used unless otherwise indicated. All surgical operations were performed under anesthesia to minimize discomfort and suffering. The mouse studies were not randomized and not blinded. For the skin expansion model, only male mice were used, consistent with our previous study^34^; in all other experiments, both male and female mice were used.

All mouse lines used in this study were maintained on the C57BL/6J genetic background. Wild-type mice (7-8 weeks old) were obtained from Shanghai SLAC Laboratory Animal. The following transgenic mouse lines were obtained from commercial vendors: *Lgr5-CreERT2*^69^ (GemPharmatech, Strain NO: T003768), *Rosa26-LSL-tdTomato-DTR* (Shanghai Model Organisms, Strain NO: NM-KI-210094), *Col1a2-CreER*^70^ (The Jackson Laboratory, Stock NO: 029567), *Pdgfra-Dre* (Shanghai Model Organisms, Strain NO: NM-KI-220193), *Rosa26-RSR-LSL-tdTomato-DTR* (Shanghai Model Organisms, Strain NO: NM-KI-190086)^36,71^, *Lgr5*^fl/fl^ (GemPharmatech, Strain NO: T006315), *Yap*^fl/fl^*;Taz*^fl/fl^ (The Jackson Laboratory, Stock NO: 030532)^72^, *Fos*^fl/fl^ (GemPharmatech, Strain NO: T005760), *Lep*^ob/ob^ (GemPharmatech, Strain NO: T001461).

The *Rosa26-LSL-CAG-Lgr5* conditional knock-in mouse line was generated in this study by Cyagen Biosciences via CRISPR-Cas9-mediated homologous recombination at the endogenous *Rosa26* locus. Briefly, a donor vector containing the “CAG promoter-loxP-PGK-Neo-6×SV40 pA-loxP-Kozak-Mouse *Lgr5* CDS-WPRE-BGH pA” cassette was co-injected with *Rosa26*-targeting gRNA (sequence: 5′-CTCCAGTCTTTCTAGAAGATGGG-3′) and Cas9 mRNA into fertilized C57BL/6J mouse eggs. F0 founder mice were screened by PCR using two primer pairs targeting the 5′ and 3′ junctions of the integrated cassette (Primer Pair 1: F1 5′-AAAGATCGCTCTCCACGCCCTAG-3′, R1 5′-GATGGGGAGAGTGAAGCAGAACG-3′, expected mutant band: 2.8 kb; Primer Pair 2: F2 5′-CTGTCCATTAACTCGGACGATGT-3′, R2 5′-ATGGGAAGTTAGTAGCAAACAAGAG-3′, expected mutant band: 3.3 kb).

### Human studies

Human tissue samples were obtained from discarded surgical specimens collected during plastic and reconstructive surgery procedures at Shanghai 9th People’s Hospital. The study included 6 individuals who underwent skin expansion (age range: 4–48 years; 3 males, 3 females) and 15 women with striae (age range: 18–49 years). Written informed consent was obtained from all participants. The influence of gender or sex was not investigated in this study.

### Cell culture

Primary HFFs and MDFs were isolated from human foreskin and newborn mouse skin tissues, respectively, following the primary dermal cell isolation protocol as described below. HFFs, MDFs, and HEK293T cells were maintained in complete Dulbecco’s modified Eagle’s medium (DMEM) containing 10% (v/v) fetal bovine serum (FBS) and 1% (v/v) penicillin-streptomycin. All cells were used at low passage, maintained below 70% confluency, and cultured at 37 °C in a humidified atmosphere with 5% CO₂.

### Skin expansion mouse model

The mouse skin expansion model was adapted from a previously reported protocol^34^. Briefly, mice were anesthetized, and the dorsal skin was disinfected. A 1-cm transverse incision was made in the cervical dorsal skin to create a subcutaneous pocket, into which a sterile silicone expander (Wanhe Plastic Materials, 0002621) was implanted. The incision was then sutured. Mechanical loading was initiated immediately after surgery by injecting sterile water into the expander and was sequentially increased according to the following two protocols:

- Standardized expansion: An initial injection of 0.6 mL sterile water was followed by subsequent injections of 0.3 mL every 4 days.
- Excessive expansion: An initial injection of 1.0 mL sterile water was followed by subsequent injections of 0.5 mL every 4 days.

Except where specified, the standardized expansion protocol was applied throughout the study. Any mouse that developed skin breakdown exceeding 50% of the expansion area prior to the scheduled endpoint was euthanized and excluded from the study. Skin tissues overlying the apex of the expansion dome, which experiences the most stable mechanical stress, were harvested at specified time points for analysis.

### Wound healing mouse model

Two independent full-thickness excisional wound models (dorsal and tail) were established. For all procedures, mice were anesthetized with isoflurane, and the surgical site was disinfected with 75% ethanol. The dorsal wound model was created by making an 8-mm diameter circular, full-thickness wound at the midline center of the back using a biopsy punch. The tail wound model was created by making a full-thickness rectangular wound (3 mm × 10 mm, width × length) on the dorsal side of the tail, 15 mm from the body, using a scalpel^16^. For both models, any immediate bleeding was controlled, and the wound bed was cleared. Wound healing was documented photographically at serial time points using a Sony α6000L camera. The wound area was quantified using ImageJ software, and the percentage of wound closure was calculated for each animal as [(initial wound area–wound area at indicated time point) / initial wound area] × 100%.

### Bleomycin-induced skin fibrosis

Skin fibrosis in mice was induced by daily subcutaneous injections of bleomycin (200 μL of a 1 mg/mL solution) (APExBIO, A8331) for two weeks^73^. Subcutaneous injections of PBS served as controls.

### Imiquimod-induced skin inflammation

To induce psoriatic inflammation, the shaved back skin of mice was treated daily with 5% imiquimod cream (Med-shine Pharma, H20030129) for 5 consecutive days^74^. Mice treated with the cream vehicle served as controls.

### Young’s modulus measurement

The Young’s modulus of skin samples was measured using a FastScan Bio atomic force microscope (AFM) (Bruker, MA, USA). For mouse skin, fresh tissues were collected, and the subcutaneous fat was carefully removed. The samples were kept in ice-cold PBS and prepared for measurement with the dermal side up. For human clinical specimens, measurements were performed on prepared fresh-frozen sections. All measurements were conducted by instrumental operators from the Instrumental Analysis Center of Shanghai Jiao Tong University who were blinded to the experimental design, following a standardized procedure. For each sample, 3-6 random locations were measured, and the average value was calculated to represent the individual’s Young’s modulus. Force curves were processed and analyzed using the NanoScope_Analysis_x86 software (Bruker) in accordance with the manufacturer’s instructions.

### Measurement of tensile strength

Biomechanical properties of skin tissues were assessed by measuring the ultimate tensile strength using a universal testing machine (Shimadzu AGS-X, Japan). The protocol was adapted from established methods^44^. Skin samples, comprising either freshly dissected mouse tissues or clinical specimens that were snap-frozen and gradually thawed on the day of testing, were cut into rectangular strips with a consistent orientation (0.3-0.6 cm wide × 1.5 cm long). Samples were maintained in PBS at 4 °C and tested within the same day. For larger specimens, two technical replicates were prepared and tested to obtain an average value. To ensure secure gripping and prevent slippage, the longitudinal ends of each strip were affixed to sandpaper with cyanoacrylate adhesive, leaving a 1 cm central region for uniaxial elongation. A tensile force was then applied at a constant displacement rate of 20 mm/min until tissue failure, identified by a conspicuous drop in the stress-strain curve. The ultimate tensile strength (UTS) in MPa was calculated as the force at failure (N) divided by the original cross-sectional area (mm²). Any specimens that exhibited technical artifacts, such as slippage at the grips or adhesive contact with the active measurement zone, were excluded from subsequent analysis.

### Drug administration in mice

Tamoxifen induction in transgenic mice was achieved using one of the following protocols. For most experiments, tamoxifen (Sigma-Aldrich, D4693) was dissolved in corn oil (MCE, HY-Y1888) at 10 mg/mL and administered via intraperitoneal injection at 200 mg/kg daily for three consecutive days. For the comparative analysis of skin across different anatomical regions under homeostasis, mice instead received an AIN-76A maintenance diet containing 400 ppm tamoxifen (ReadyDietech, RD21102601) for three consecutive weeks. This dietary approach was employed to ensure a uniform and sustained induction while avoiding the acute stress associated with injection.

For genetic ablation of LGR5^*+*^ MIRFs, *Lgr5*-DTR mice received intraperitoneal injections of diphtheria toxin (DT) at 15 µg/kg for three consecutive days.

For topical treatment with Ruxolitinib (Selleck, 941678-49-5) or Upadacitinib (Selleck, 1310726-60-3), mice were treated every other day for 20 consecutive days on the shaved expanded skin using a hydrogel vehicle (1% sodium hyaluronate and 20% propylene glycol in water) or the same vehicle containing 0.5% (w/v) JAK inhibitor (Ruxolitinib or Upadacitinib), which was first dissolved in DMSO and then mixed with the hydrogel vehicle at a ratio of 9:1 (v/v) immediately prior to application.

For intradermal administration of XAV-939 (MCE, 284028-89-3), GsMTx4 (MCE, HY-P1410), and verteporfin (MCE, 129497-78-5), each mouse received a total volume of 60 µL of the respective drug solution (0.5 mg/mL) via intradermal injection at three separate points around the expanded skin. The treatment was performed every other day for 8 consecutive days.

### Dermal-epidermal separation

Following adipose tissue removal, freshly collected mouse or human skin samples were washed with DPBS and cut into 3-5 mm² pieces. The pieces were digested with Dispase II (5 mg/mL, Roche) at 37 °C for 1 h or at 4 °C overnight. The dermis and epidermis were then mechanically dissociated using forceps. The isolated tissues were then further processed either by enzymatic digestion to obtain primary cells, or by homogenization for RNA or protein extraction.

### Primary dermal cell collection

The dermal tissue was separated as described above and then digested in HBSS containing Collagenase (2.5 mg/mL) and DNase I (50 U/mL) at 37 °C for 2 h. To obtain a single-cell suspension, the digested tissue was repeatedly aspirated and dispensed with a pipette (~20 times) followed by sequential filtration through 70 μm and 40 μm cell strainers. Cells were collected by centrifugation, washed with DPBS, and resuspended in complete culture medium for immediate use or further analysis.

### Matrix contraction assay

The matrix contraction assay was adapted from a previously reported protocol with minor modifications^75^. Following cell preparation, a collagen working solution was prepared on ice by mixing 3 mg/mL rat tail type I collagen (Thermo Fisher Scientific, A1048301), DMEM, fetal bovine serum, and 1 N NaOH at a volume ratio of 1:1.65:0.3:0.05. For each contraction assay, 2×10⁵ cells (HFFs, MDFs, or primary dermal cells) were resuspended in 1 mL of the prepared collagen solution and plated into a well of a 24-well plate. The plate was then incubated at 37 °C with 5% CO₂ for 1 h to allow for gel polymerization. After solidification, 1 mL of fresh DMEM supplemented with 10% FBS was carefully added on top of each gel. Where indicated, the medium was supplemented with rhIFNα2a (1000 U/mL), rhIFNβ (1000 U/mL), mIFNβ (1000 U/mL), or the inhibitor ruxolitinib (5 μM). To initiate contraction, gels were gently released from the well walls using a bent needle. Gel images were captured at a single endpoint (12-48 h later), and the gel area was quantified using ImageJ software (National Institutes of Health, Bethesda, MD). The contraction rate was calculated as the percentage of area reduction relative to the original well area.

### Cell treatment

Cellular senescence was induced using three distinct models: for genotoxic stress, MDFs or HFFs were treated with 50 μg/mL bleomycin (APExBIO, A8331) for 20 h and then cultured for an additional 7‑10 days; for UVB‑induced senescence, HFFs were exposed to 100 mJ/cm² UVB irradiation (SGLinker UV Crosslinker, SAGECREATION) once daily for three consecutive days; and replicative senescence was modeled using late‑passage MDFs (passage >10).

To activate CreER‑mediated recombination in MDFs, 4‑hydroxytamoxifen (MCE, HY‑16950) was added to the culture medium at 1 μM and maintained for 48 h.

For cytokine and recombinant protein treatments, cells were incubated with rhIFNα2a (1000 IU/mL) for 30 or 60 min, rhIFNβ (1000 IU/mL) for 30 or 60 min, mIFNβ (1000 IU/mL) for 30 or 120 min, or rhR‑spondin‑1 (0.5 or 2 nM) for 24 h.

Stable gene overexpression was achieved via lentiviral transduction using the vector pHBLV‑CMV‑MCS‑3×FLAG‑EF1‑ZsGreen‑T2A‑PURO, into which human *LGR5*, human *YAP* (encoding the p.S127A mutant), human *FOS*, and mouse *Lgr5* had been cloned. Lentiviral particles and controls (Hanheng Biotechnology, Shanghai, China) were applied to cells at ~50% confluence in 5 mL medium containing 10 μg/mL polybrene (Hanheng Biotechnology, HB‑PB‑500); after 4 h, the volume was brought to 10 mL with complete medium. The virus‑containing medium was replaced with fresh medium after 24 h, and selection with 1 μg/mL puromycin was performed for 48 h starting 72 h post‑transduction. For transient transfection, HFFs were transfected with plasmids using EZ trans Plus reagent (Life‑iLab, AC04L011), and cells were harvested or assayed 48 h after transfection.

### In vitro immune cell migration assay

Spleens were isolated from 8-week-old wild-type male mice. Splenocytes were obtained by mechanical dissociation, followed by treatment with red blood cell lysis buffer (Beyotime, C3702) according to the manufacturer’s instructions and resuspended at 1×10⁶ cells/mL. For the migration assay, 100 µL of splenocyte suspension was added to the upper chamber of 24-well Transwell plates. MDFs were seeded in the lower chambers at 1×10⁴ or 2×10⁴ cells per well, and cultured in medium with or without 1000 U/mL mIFNγ. After 48 h of incubation at 37 °C with 5% CO₂, migrated cells in the lower chamber were collected by gentle pipetting and scraping, and then quantified by flow cytometry using the protocols described below.

### Flow cytometry

For immune profiling of mouse dermal tissue, single-cell suspensions were collected as mentioned previously, and stained with Fixable Viability Dye eFluor 780 (BioLegend, 65-0865-18) followed by staining with an antibody cocktail containing mouse Fc block (BD Biosciences, 553141) on ice for 30 min. The following antibodies were used: PE anti-CD45 (1:100, BioLegend, 103105), PE/Cyanine 7 anti-CD3 (1:100, BioLegend, 100219), FITC anti-CD11b (1:100, BioLegend, 101205), APC anti-Ly6G (1:100, BioLegend, 127613), BV605 anti-MHC II (1:100, BioLegend, 107639).

For quantification of migrated immune cells in vitro, harvested cells were stained with Zombie Aqua Dye (BioLegend, 77143) followed by staining with PE anti-CD45 (1:100, BioLegend, 103105) containing Fc block on ice for 30 min. To normalize samples, Precision Count Beads (BioLegend, 424902) were added to all samples before counting by flow cytometry.

For cell cycle and proliferation analysis, cultured MDFs were pulsed with 10 µM EdU for 3 h prior to collection and then processed using the BeyoClick™ EdU Cell Proliferation Kit (Beyotime) according to the manufacturer’s instructions. Briefly, cells were stained with the kit’s EdU reaction mixture followed by Hoechst 33342 for nuclear counterstaining.

All samples were analyzed on a CytoFLEX LX flow cytometer (Beckman Coulter), and data were processed using FlowJo software. Gating strategies are provided in the Supplementary Methods.

### Immunoblotting (IB) and immunoprecipitation (IP)

Tissue or cellular lysates were prepared using RIPA lysis buffer (Epizyme, PC101) supplemented with a protease and phosphatase inhibitor cocktail (Beyotime, P1045). After centrifugation to remove debris, the clarified supernatants were denatured by heating at 95 °C for 10 min and stored at −80 °C until use. For immunoblotting, protein samples were separated by electrophoresis under denaturing conditions on 4-20% gradient SurePAGE gels (Epizyme, LK310) at 120 V for 60 min and then transferred onto PVDF membranes at 300 mA for 100 min. The membranes were blocked with blocking solution (Epizyme, PS108) for 1 h before incubation with specific primary antibodies overnight at 4 °C. Following washes, membranes were incubated with appropriate horseradish peroxidase (HRP)-conjugated secondary antibodies for 1 h at room temperature. To optimize antibody usage and enable multiplex detection, membranes were routinely sectioned according to molecular weight markers prior to probing. Equal protein loading was verified for all samples, and protein bands were visualized using an ECL solution (Epizyme, SQ201). For subcellular fractionation, nuclear and cytoplasmic fractions were isolated using NE-PER™ extraction reagents (Thermo Scientific, 78833) according to the manufacturer’s instructions, followed by the standard immunoblotting procedure. For immunoprecipitation, HFFs were lysed in IP lysis buffer (Yeasen, 20118ES60) containing the protease and phosphatase inhibitor cocktail (Beyotime, P1045). Cleared supernatants were incubated with the indicated BeyoMag™ Magnetic Beads (Beyotime, P2115, P2118, P2173, or P2171) overnight at 4 °C. The beads were washed extensively, and the immunoprecipitated proteins were eluted, denatured, and analyzed by standard immunoblotting.

The following primary antibodies were employed: GAPDH (Rabbit, 1:3000, Epizyme, LF206), LGR5 (Rabbit, 1:1000, abcam, ab75850), YAP (Rabbit, 1:1000, Cell Signaling Technology, 14074), COL1A1 (Rabbit, 1:1000, Cell Signaling Technology, 72026), COL3A1 (Rabbit, 1:1000, Cell Signaling Technology, 63034), ELN (Mouse, 1:1000, Santa Cruz Biotechnology, sc-58756), αSMA (Rabbit, 1:1000, Cell Signaling Technology, 19245), Lamin A/C (Rabbit, 1:3000, Proteintech, 10298-1), β-catenin (Rabbit, 1:1000, Cell Signaling Technology, 8480), JAK1 (Mouse, 1:1000, Cell Signaling Technology, 50996), pJAK1 (Rabbit, 1:1000, Cell Signaling Technology, 3331), STAT1 (Rabbit, 1:1000, Cell Signaling Technology, 9172), pSTAT1 (Rabbit, 1:1000, Cell Signaling Technology, 9167), STAT2 (Rabbit, 1:1000, HUABIO, ET1702-18), pSTAT2 (Rabbit, 1:1000, Cell Signaling Technology, 4441), STAT3 (Mouse, 1:1000, Santa Cruz Biotechnology, sc-8019), pSTAT3 (Rabbit, 1:1000, Cell Signaling Technology, 9145), MMP3 (Mouse, 1:1000, Proteintech, 66338-1), DYKDDDDK Tag/Flag (Rabbit, 1:1000, Cell Signaling Technology, 14793), Myc Tag (Rabbit, 1:1000, Cell Signaling Technology, 2276), c-FOS (Rabbit, 1:1000, Abcam, ab222699), c-JUN (Rabbit, 1:1000, Cell Signaling Technology, 9165), TAZ (Mouse, 1:1000, Santa Cruz Biotechnology, sc-518026), β-actin (Mouse, 1:3000, Epizyme, LF201), p16 (Mouse, 1:1000, Santa Cruz Biotechnology, sc-1661).

Representative IB and IP results shown in the figures were obtained from three independent experiments with similar results. Uncropped scans of all blots from the main figures are provided in the accompanying Source data. Uncropped scans for Supplementary Figures. are included at the end of the Supplementary Information.

### Mass spectrometry assay (MS)

Protein samples immobilized on anti-Flag or anti-IgG beads were subjected to reduction with DTT and alkylation with iodoacetamide, followed by overnight tryptic digestion at 37 °C. Peptides were extracted, desalted using C18 solid-phase extraction, and vacuum-dried. The dried peptides were reconstituted in 0.1% formic acid and separated on an EASY-nLC 1000 system with a C18 analytical column. Mass spectrometry analysis was performed on an Orbitrap Fusion Lumos instrument in data-dependent acquisition mode, with full scans at 60,000 resolution (m/z 350–1600) and HCD fragmentation at 30% collision energy. Raw data were processed using Proteome Discoverer 2.4 and searched against the SwissProt human database with trypsin specificity, carbamidomethylation as a fixed modification, and acetylation/oxidation as variable modifications. Peptide and protein identifications were filtered at 1% FDR. Identified proteins were compared between groups using Venn diagram analysis. Proteins specifically enriched in the Anti-Flag group along with related specificity metrics are listed in Supplementary Data 1.

### Plasmid construction

All plasmids were constructed using seamless cloning and verified by Sanger sequencing. The plasmids generated in this study include:

- pGL3-Basic-*LGR5* promoter: The core human LGR5 promoter was cloned into the pGL3-Basic vector.
- pGL3-Basic-*LGR5* promoter-*LGR5* enhancer^WT^: The candidate human LGR5 enhancer sequence was inserted downstream of the luciferase gene in the plasmid above.
- pGL3-Basic-*LGR5* promoter-*LGR5* enhancer^MT^: A mutant version was created by site-directed mutagenesis of the core enhancer binding site to “GTCAGAC”.
- pcDNA3.1- *YAP*^S127A^: Human *YAP1* CDS containing the constitutively-activated p.S127A mutation was inserted between the BamHI and XbaI sites.
- pcDNA3.1-Flag-*LGR5*-WT: Full-length human *LGR5* was inserted between the EcoRI and BamHI sites of pcDNA3.1-EF1a-mcs-3flag-CMV-EGFP.
- pcDNA3.1-Flag-*LGR5*-ΔN: Human *LGR5* lacking the N-terminal domain (residues 562–907) was inserted between the EcoRI and BamHI sites of pcDNA3.1-EF1a-mcs-3flag-CMV-EGFP^76^.
- pcDNA3.1-Flag-*LGR5*-ΔC: Human *LGR5* lacking the C-terminal domain (residues 1–836) was inserted between the EcoRI and BamHI sites of pcDNA3.1-EF1a-mcs-3flag-CMV-EGFP^76^.
- pcDNA3.1-Flag-*LGR5*-A190D: Human *LGR5* with the p.A190D point mutation was inserted between the EcoRI and BamHI sites of pcDNA3.1-EF1a-mcs-3flag-CMV-EGFP^46^.
- pcDNA3.1-Flag-*LGR5*-ECD: A chimeric construct containing the human *LGR5* ectodomain (residues 1–561) fused to the transmembrane domain of human GPA33 (residues 234–319) was inserted between the EcoRI and BamHI sites of pcDNA3.1-EF1a-mcs-3flag-CMV-EGFP^46,77^.
- pcDNA3.1-Myc-*JAK1*: Human *JAK1* CDS with C-terminal Myc tag was inserted between the BamHI and XhoI sites of pcDNA3.1-T2A-RFP.

The plasmids generated in this study are available from the corresponding authors upon reasonable request.

### Luciferase activity assay

For luciferase assays, HEK293T cells in the logarithmic growth phase were seeded into 24-well plates. When cells reached approximately 70% confluency, they were co-transfected using GMTrans Liposomal Transfection Reagent (Genomeditech, TG-10015) according to the manufacturer’s instructions. Each transfection mixture contained 250 ng of the indicated pGL3-based firefly luciferase reporter plasmid, 250 ng of either the YAP^S127A^ expression plasmid or an empty vector control (pcDNA3.1), and 25 ng of the pRL-TK plasmid (expressing Renilla luciferase for normalization) in Opti-MEM (Gibco, 31985070).

At 48 h post-transfection, cells were washed with PBS and lysed with cell lysis buffer. The lysates were centrifuged for 5 min at 4 °C to remove cellular debris. Firefly and Renilla luciferase activities were measured sequentially from the same supernatant aliquot using a commercial Dual-Luciferase Reporter Assay Kit (Genomeditech, GM-040502A) on a SpectraMax L microplate reader (Molecular Devices). Firefly luciferase activity for each sample was normalized to the corresponding Renilla luciferase activity to control for transfection efficiency. The relative luciferase activity was then calculated by setting the normalized value of the control group (co-transfected with empty reporter vector and empty expression vector) to 1.

### Molecular docking

The three-dimensional structures of JAK1 and LGR5, modeled with AlphaFold 3^78^, were prepared and employed in global protein-protein docking using GRAMM^79^. The resulting decoys were clustered and scored, and the top-ranked cluster was selected for detailed analysis of the interaction interface.

### RNA extraction and qRT–PCR analysis

Following the manufacturers’ protocols, we extracted total RNA with TRIzol reagent (Invitrogen, 15596018CN) and reverse-transcribed it into cDNA using a dedicated kit (Tiangen, KR118-02). Gene expression was quantified by real-time PCR on an Applied Biosystems instrument using qPCR SYBR Green Master Mix (Yeasen, 11202ES08). Data analysis employed the 2^−ΔΔCt^ method, normalizing to GAPDH as an internal reference. The corresponding primer sequences are provided in Supplementary Data 2.

### Mouse genotyping

Offspring of transgenic mice were genotyped by PCR analysis of toe-derived DNA. DNA was extracted with a mouse tissue lysis kit (Yeasen, 19697ES70), followed by PCR using a 2× HotStart PCR genotyping master mix (Yeasen, 10108ES03). The sequences of genotyping primers are listed in Supplementary Data 3.

### CUT&RUN sequencing and bioinformatics analysis

HFFs were harvested and bound to Concanavalin A-coated magnetic beads. Permeabilized cells were then incubated with a primary antibody against YAP (Rabbit, Cell Signaling Technology, 14074) overnight at 4 °C. After washing, the protein A/G-MNase enzyme was added to bind the antibody. Following a second incubation, targeted chromatin digestion was initiated by the addition of CaCl₂. The reaction was stopped, and the released DNA fragments were purified. Libraries for next-generation sequencing were constructed from the purified DNA. The quality and concentration of the final libraries were assessed using Qubit 3.0 and Agilent 2100 Bioanalyzer. Qualified libraries were sequenced on an Illumina platform with a 150-bp paired-end strategy.

Raw sequencing reads were first processed with Trim Galore to remove adapter sequences and low-quality bases. Quality control was performed using FastQC. The cleaned reads were then aligned to the human reference genome (hg38) using BWA-MEM (v0.7.17). Duplicates were marked, and uniquely mapped reads were retained for subsequent analysis. Peak calling was performed using MACS2 (v2.2.6) with a q-value threshold of 0.05, comparing the YAP samples against the IgG control to identify significant enrichment regions. The Fraction of Reads in Peaks (FRiP) was calculated for each sample to assess data quality. Biological replicates showed high reproducibility, as confirmed by Pearson correlation analysis using deepTools. Differential binding analysis between conditions was conducted using DESeq2, with peaks exhibiting an adjusted *p*-value (padj) < 0.05 considered statistically significant. De novo motif discovery within the YAP-binding peaks was performed using HOMER (v4.10). Genomic visualization of the binding profiles was performed using IGV 2.19.4.

### RNA sequencing and bioinformatics analysis

For bulk RNA-seq data generated in this study, total RNA was isolated from cultured HFFs or MDFs, with integrity verified using an Agilent Bioanalyzer 2100 system. Sequencing libraries were prepared using the standard Illumina protocol: poly(A)+ mRNA was enriched, fragmented, and reverse-transcribed into double-stranded cDNA. After end repair, adenylation, and adapter ligation, cDNA fragments of 370-420 bp were size-selected and PCR-amplified. Libraries were sequenced on an Illumina platform to generate 150-bp paired-end reads. Raw reads were quality-controlled and adapter-trimmed using fastp. Clean reads were aligned to the GRCh38 (human) or GRCm39 (mouse) reference genome using HISAT2. Read counts for each gene were generated using featureCounts, and FPKM values were calculated to quantify gene expression levels. Differential gene expression analysis was performed using the DESeq2 R package. Heatmaps were generated using the pheatmap R package. Volcano plots were generated using the ggplot2 R package. Differentially expressed genes (DEGs) for volcano plot visualization and Gene Ontology (GO) analysis were defined as genes with an absolute log_2_ fold change (|log_2_FC | ) > 1.2 and adjusted *P*-value < 0.05.

Published scRNA-seq datasets, including fibroblasts from the mouse skin expansion model (GSE281659) and neonatal mouse dorsal skin (GSE189241)^34,80^, were reanalyzed. For GSE281659, fibroblasts from days 8 and 36 post-expansion were pooled to analyze the functional role of *Lgr5*^*+*^ fibroblasts. *Lgr5*^*+*^ and *Lgr5*^-^ subsets were defined based on the median expression level of *Lgr5*. To identify high-confidence intersecting candidates, DEGs were defined using the following thresholds: |log_2_FC | > 0.585 (equivalent to a linear fold change > 1.5) and adjusted *P*-value < 0.01. For GO enrichment analysis, a more inclusive threshold (|log_2_FC | > 0.25 and adjusted *P*-value < 0.01) was applied to capture broader biological processes. For GSE189241, neonatal fibroblast data were reanalyzed using the online tool CytoNavigator.

The Xenium data reanalyzed in this study were deposited in GSE289263. Spatial transcriptomic analysis was performed using transcript-level data (transcripts.csv.gz). High-quality transcripts (quality value ≥ 40) were retained, and unassigned codewords were excluded. Spatial gene expression was visualized by mapping individual transcripts to their original spatial coordinates.

GO enrichment analysis was performed using the clusterProfiler R package with the enrichGO function. Gene Set Enrichment Analysis (GSEA) was conducted on both bulk RNA-seq and scRNA-seq using GSEA software (v4.2.3) in pre-ranked mode^81^. Gene sets for GSEA used in this study originated from https://www.gsea-msigdb.org/gsea/msigdb, including “WP: Overview of proinflammatory and profibrotic mediators”, “Hallmark: Inflammatory response”, “Reactome: Chemokine receptors bind chemokines”, “GO_BP: Collagen metabolic process”, “GO_BP: Protein localization to extracellular region”, “Reactome: Collagen biosynthesis and modifying enzymes”, “Reactome: ECM proteoglycans”, “Hallmark: Wnt beta catenin signaling”, “KEGG: JAK-STAT signaling pathway”, “Hallmark: Interferon alpha response”, “Hallmark: Interferon gamma response”, “Reactome: Signaling by Wnt”.

### Histological evaluation and immunohistochemistry (IHC)

Standard paraffin embedding was performed, encompassing tissue fixation, dehydration, clearing, paraffin infiltration, and embedding, after which sections were cut and mounted on slides for histological and immunohistochemical (IHC) analyses. All staining procedures were conducted by Servicebio (Wuhan, China).

For hematoxylin and eosin (H&E) staining, mounted sections were first deparaffinized in xylene and rehydrated through a graded ethanol series. After washing, the sections were stained with hematoxylin, differentiated, blued, and subsequently counterstained with eosin. Following dehydration and clearing, the slides were sealed with neutral balsam. Masson’s trichrome staining was carried out on deparaffinized and rehydrated sections. The protocol involved sequential staining with Weigert’s iron hematoxylin for nuclei, a Biebrich scarlet-acid fuchsin mixture for cytoplasm and muscle fibers, phosphomolybdic acid for differentiation, and aniline blue for collagen. Finally, sections were dehydrated, cleared, and mounted. Image quantification was performed with ImageJ software. Dermal thickness was defined as the distance between the epidermal-dermal junction and the dermal-adipose junction.

For IHC, sectioned samples underwent deparaffinization and rehydration. Antigen retrieval was performed by heating the slides in citric acid buffer at 95 °C. Endogenous peroxidases were quenched with 3% H₂O₂, and non-specific binding sites were blocked with 10% goat serum. The sections were then incubated with primary antibodies at 4 °C overnight. After washing, the signals were developed using appropriate secondary antibodies, streptavidin-peroxidase, and a DAB substrate, followed by hematoxylin counterstaining. The primary antibodies used in this study were: LGR5 (Rabbit, 1:200, Affinity, DF2816) and Ki67 (Rabbit, 1:200, Abcam, ab15580).

### Immunofluorescence (IF) staining

For immunofluorescence (IF) analysis of tissues, cryosections were prepared from OCT-embedded frozen samples. Sections or cultured cells were fixed with 4% paraformaldehyde for 10 min at room temperature. All samples were then blocked and permeabilized for 1 h at room temperature in a buffer containing 2.5% normal donkey serum, 2.5% normal goat serum, 1% BSA, and 0.3% Triton X-100. Subsequently, samples were incubated with primary antibodies overnight at 4 °C, followed by species-matched fluorochrome-conjugated secondary antibodies for 1 h at room temperature. After washing, samples were mounted with DAPI Fluoromount-G™ (Beyotime) and imaged. Image acquisition was performed on a Zeiss Axio Imager A2 microscope.

The following primary antibodies were used: K14 (Chicken, 1:500, BioLegend, 906004), RFP/tdTomato (Rabbit, 1:1000, Rockland, 600-401-379), CD45 (Rat, 1:200, BioLegend, 103101), αSMA (Rabbit, 1:200, Cell Signaling Technology, 19245), PDGFRA (Goat, 1:100, R&D Systems, AF1062), Vimentin (Mouse, 1:200, Santa Cruz Biotechnology, sc-6260), Perilipin-1 (Rabbit, 1:200, Cell Signaling Technology, 9349), K10 (Rabbit, 1:500, BioLegend, 905404), F4/80 (Rat, 1:200, BioLegend, 123102), Sca1 (Rat, 1:200, Santa Cruz Biotechnology, sc-52601), Dlk1 (Mouse, 1:200, Santa Cruz Biotechnology, sc-376755), CD31 (Rabbit, 1:200, Servicebio, GB113151), DPP4/CD26 (Rabbit, 1:200, Servicebio, GB115320), pSTAT1 (Rabbit, 1:200, Cell Signaling Technology, 9167).

### In vivo EdU incorporation assay

For the EdU incorporation experiments in vivo, adult mice were injected intraperitoneally with 50 mg/kg EdU (Beyotime, ST067) 24 h before being killed. Skin tissues were collected and prepared as frozen sections, and IF staining was performed as mentioned above. After incubation with secondary antibodies, sections were stained with the BeyoClick^TM^ EdU Cell Proliferation Kit (Beyotime, C0081) according to the manufacturer’s instructions.

### Two-color fluorescence in situ hybridization (FISH)

RNA FISH was performed using the PinpoRNA™ 2.0 Universal Multicolor Fluorescence RNA In Situ Detection Kit (Pinpoease, PIF2000). Briefly, paraffin-embedded human skin sections were baked at 60 °C, deparaffinized, rehydrated, and subjected to antigen retrieval in boiling 1× Pretreatment Solution B plus. After digestion with 1× Digestive Enzyme II at 40 °C, sections were hybridized at 40 °C for 2 h with a probe mixture containing the ready-to-use Human PDGFRA RNA Probe (Type I, Pinpoease, 51561-A1) and the Human LGR5 RNA Probe (Type II, Pinpoease, 85492-A2, diluted 1:40 in Type I hybridization buffer). Following hybridization and washes, signal amplification was carried out sequentially with Reaction 1 (40 °C, 25 min) and Reaction 2 (40 °C, 15 min). The first target (PDGFRA, Channel I) was detected using Reaction 3 Type I and a tyramide-fluorophore conjugate (room temperature, 30 min). After HRP inactivation with an HRP inhibitor, the second target (LGR5, Channel II) was detected using Reaction 3 Type II and a second tyramide-fluorophore conjugate. Nuclei were counterstained with DAPI. Finally, the sections were mounted using DAPI Fluoromount-G™ (Beyotime), and image acquisition was performed under a Zeiss Axio Imager A2 microscope.

### CCK-8 assay for cell growth

Cell viability was assayed by a CCK-8 kit (Beyotime, C0037), with the absorbance at 450 nm measured using a microplate reader.

### Gross appearance grading for mouse skin expansion

A semi-quantitative gross appearance scoring system was employed to evaluate the condition of the expanded mouse skin. The condition was graded from 0 to 4 as follows: Score 0: Healthy skin or mild erythema only. Score 1: Visible signs of inflammation (including scabbing) or skin breakdown affecting <5% of the expansion area. Score 2: Skin breakdown affecting 5–20% of the area. Score 3: Skin breakdown affecting 20–40% of the area. Score 4: Skin breakdown affecting >40% of the area. The score for each sample was determined by consensus between two independent evaluators, based on standardized photographs of the mouse expansion site taken at fixed time points.

### Statistical analysis

No statistical methods were used to predetermine sample size. The number of replicates (n) for each group is detailed in the corresponding figure legends. Unless otherwise noted, all quantitative data are presented as mean ± SEM. The normality of data distributions was verified through visual inspection of distributions (Q‑Q plots) and the Shapiro‑Wilk test. Based on this assessment, comparisons between two groups were performed using a two‑tailed unpaired Student’s *t*‑test for parametric data, with Welch’s correction where variances were unequal, or the Mann‑Whitney U test for non‑parametric data. For comparisons across more than two groups with a single independent variable, one‑way ANOVA followed by Tukey’s post hoc test was used for parametric data, or the Kruskal‑Wallis test with Dunn’s correction for non‑parametric data. For data involving two independent variables, two‑way ANOVA followed by Šidák’s multiple comparisons test was applied. For paired comparisons, a paired two‑tailed Student’s *t*‑test was used. Correlation analyses were performed using Pearson’s correlation coefficient. Statistical significance was defined as *P* < 0.05 and is denoted in figures as follows: ^, *P* > 0.05; *, *P* < 0.05; **, *P* < 0.01; ***, *P* < 0.001; ****, *P* < 0.0001. All statistical analyses were performed using GraphPad Prism 10.4.1.

### Reporting summary

Further information on research design is available in the Nature Portfolio Reporting Summary linked to this article.

## Supplementary information

Supplementary Information

Peer Review file

Description of Additional Supplementary Files

Supplementary Data 1-4

Reporting Summary

## Source data

Source Data

## Data Availability

The raw sequencing data generated in this study have been deposited in the Genome Sequence Archive (GSA) at the National Genomics Data Center (NGDC). HFF data are available in the GSA for Human repository under the accession number HRA015588. MDF data are available under the accession number CRA035643 in the GSA repository [https://ngdc.cncb.ac.cn/gsa/browse/CRA035643]. Previously published datasets analyzed in this study are listed in Supplementary Data 4. Source data are provided with this paper.

## Code availability

No custom code was developed for this study. Standard software and publicly available packages used for data analysis are described in the Methods section.
