# Supplementary material for: LGR5-positive fibroblasts orchestrate skin mechanoadaptation through JAK1 modulation
Source: Nat Commun. 2026 Sep 11;17:10733. doi: 10.1038/s41467-026-77113-y (PMC13658777; doi:10.1038/s41467-026-77113-y)

Supplementary Figures

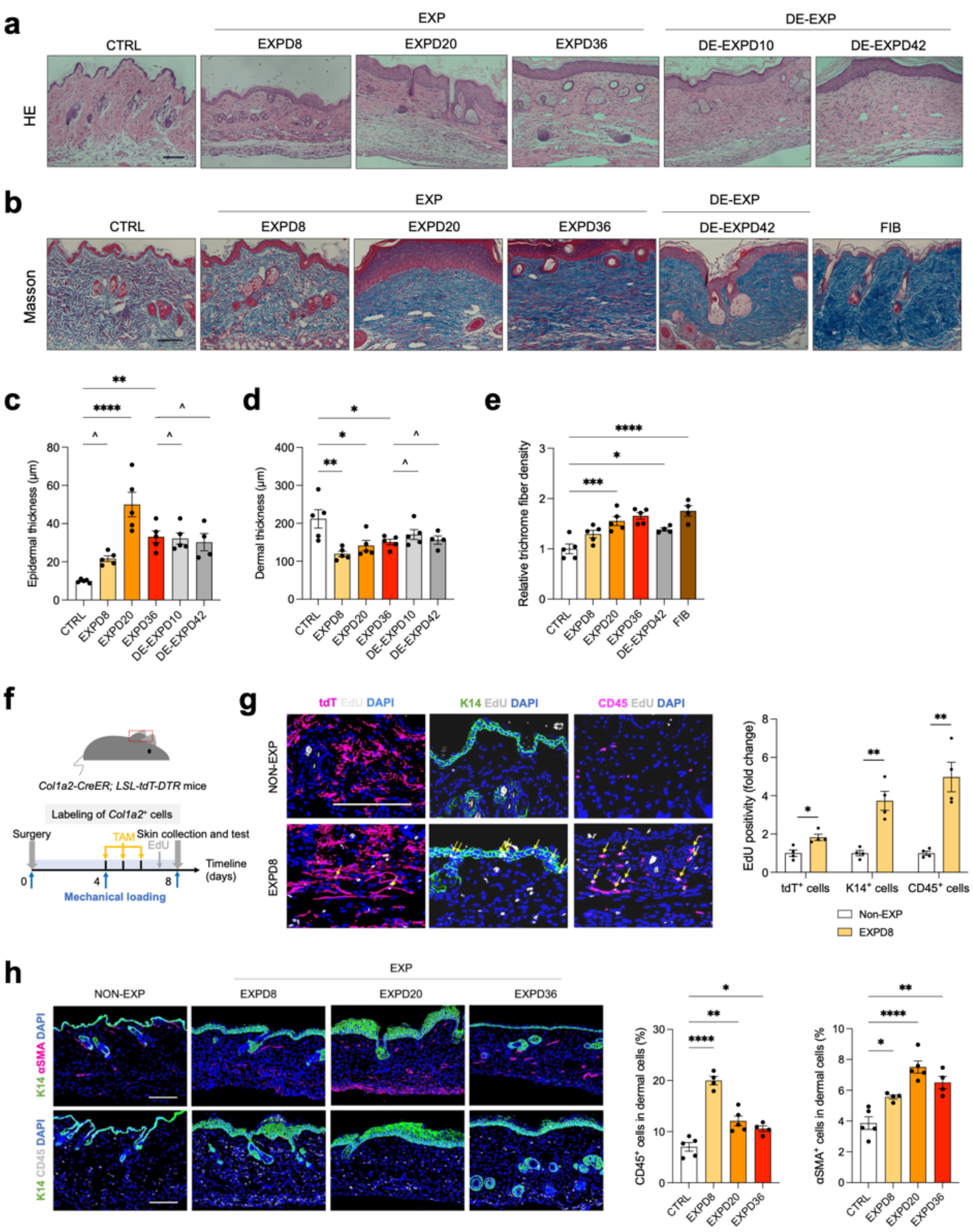

Supplementary Figure 1. The murine skin expansion model is characterized by fibro-inflammatory dermal remodeling and epidermal proliferation

a-e, H&E staining and Masson's trichrome staining of skin from indicated mice. (a) Representative images of H&E staining (scale bar, 100 μm) (n = 4–5 mice). (b) Representative images of Masson's trichrome staining (scale bar, 100 μm). FIB (bleomycin-induced skin fibrosis) skin served as a positive control. (c-e) Quantification of epidermal thickness (^*P* = 0.9943 / 0.2275 / 0.9999, ***P* = 0.0016, *****P* < 0.0001), dermal thickness (^*P* = 0.9998 / 0.9116, **P* = 0.0499 / 0.0177, ***P* = 0.0013), and relative trichrome fiber density (**P* = 0.0381, ****P* = 0.0008, *****P* < 0.0001).

f, Schematic timeline for tracing *Col1a2*$^+$ fibroblasts in *Col1a2*-tracing mice following skin expansion.

g, Immunofluorescence co-staining for EdU and indicated markers (tdT, K14, CD45) in skin from indicated *Col1a2*-tracing mice (n = 4 mice). Left, representative images (scale bar, 100 μm); right, quantification of the relative EdU positivity of tdT$^+$ fibroblasts (**P* = 0.0125), K14$^+$ basal epithelial cells (***P* = 0.0018), and CD45$^+$ immune cells (***P* = 0.0023).

h, Immunofluorescence staining for K14, CD45 and αSMA (n = 4–5 mice). Left,

representative images (scale bar, 100 μm); right, quantification of the percentage of $CD45^+$ cells (*P = 0.0471, **P = 0.0027, ****P < 0.0001) and αSMA^+ cells (*P = 0.0287, **P = 0.0010, ****P < 0.0001).
Data are presented as mean ± SEM. Statistical significance was determined by one-way ANOVA with Tukey's post hoc test or two-tailed unpaired Student's t-test. Source data are provided as a Source Data file.

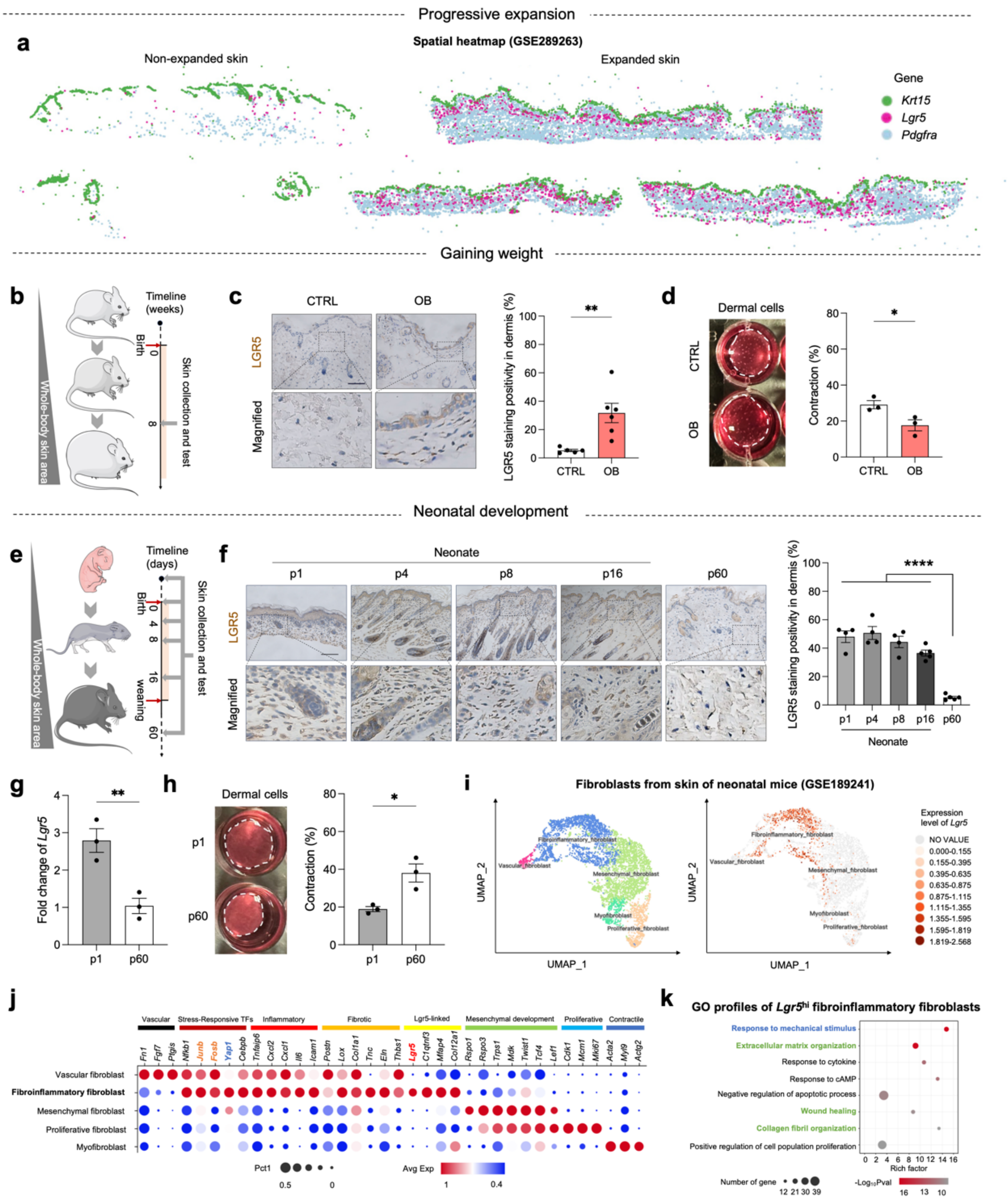

Supplementary Figure 2. LGR5^{+} MIRFs are induced across different stretching scenes

a, Spatial map of *Krt15*, *Lgr5* and *Pdgfra* from spatial Xenium assay. Biological replicates of non-expanded and expanded mouse skin are shown.

b, Schematic timeline of weight gain in *ob/ob* mice (OB) and sample collection. Images provided by Servier Medical Art (https://smart.servier.com), licensed under CC BY 4.0 (https://creativecommons.org/licenses/by/4.0/).

c, IHC staining for LGR5 in skin of control and OB mice (n = 5–6 mice). Left, representative images (scale bar, 100 μm); right, quantification of LGR5^{+} dermal cells (***P* = 0.0071).

d, Matrix contraction assay (n = 3 mice). Left, representative images of collagen gels; right, quantification of contraction percentage (**P* = 0.0396).

e, Schematic timeline of postnatal mouse development and sample collection. Images provided by Servier Medical Art (https://smart.servier.com), licensed under CC BY 4.0 (https://creativecommons.org/licenses/by/4.0/).
f, IHC staining for LGR5 in mouse skin at indicated postnatal days (n = 4–5 mice). Left, representative images (scale bar, 100 μm); right, quantification of $LGR5^+$ dermal cells (****P < 0.0001). P, postnatal day.
g, qPCR analysis of *Lgr5* mRNA in the dermis of indicated mice (n = 3 mice) (***P* = 0.0097).
h, Matrix contraction assay (n = 3 mice). Left, representative images of collagen gels; right, quantification of contraction percentage (**P* = 0.0186).
i, UMAP visualization of fibroblast clusters and *Lgr5* expression from neonatal mouse skin, based on scRNA-seq data.
j, Dot plot showing expression of marker genes for identified fibroblast clusters**,** based on scRNA-seq data.
k, Bubble plot showing significantly enriched GO terms in $Lgr5^+$ fibroblasts from neonatal mouse skin, based on scRNA-seq data.
Data are presented as mean ± SEM. Statistical significance was determined by one-way ANOVA with Tukey's post hoc test or two-tailed unpaired Student's t-test. Source data are provided as a Source Data file.

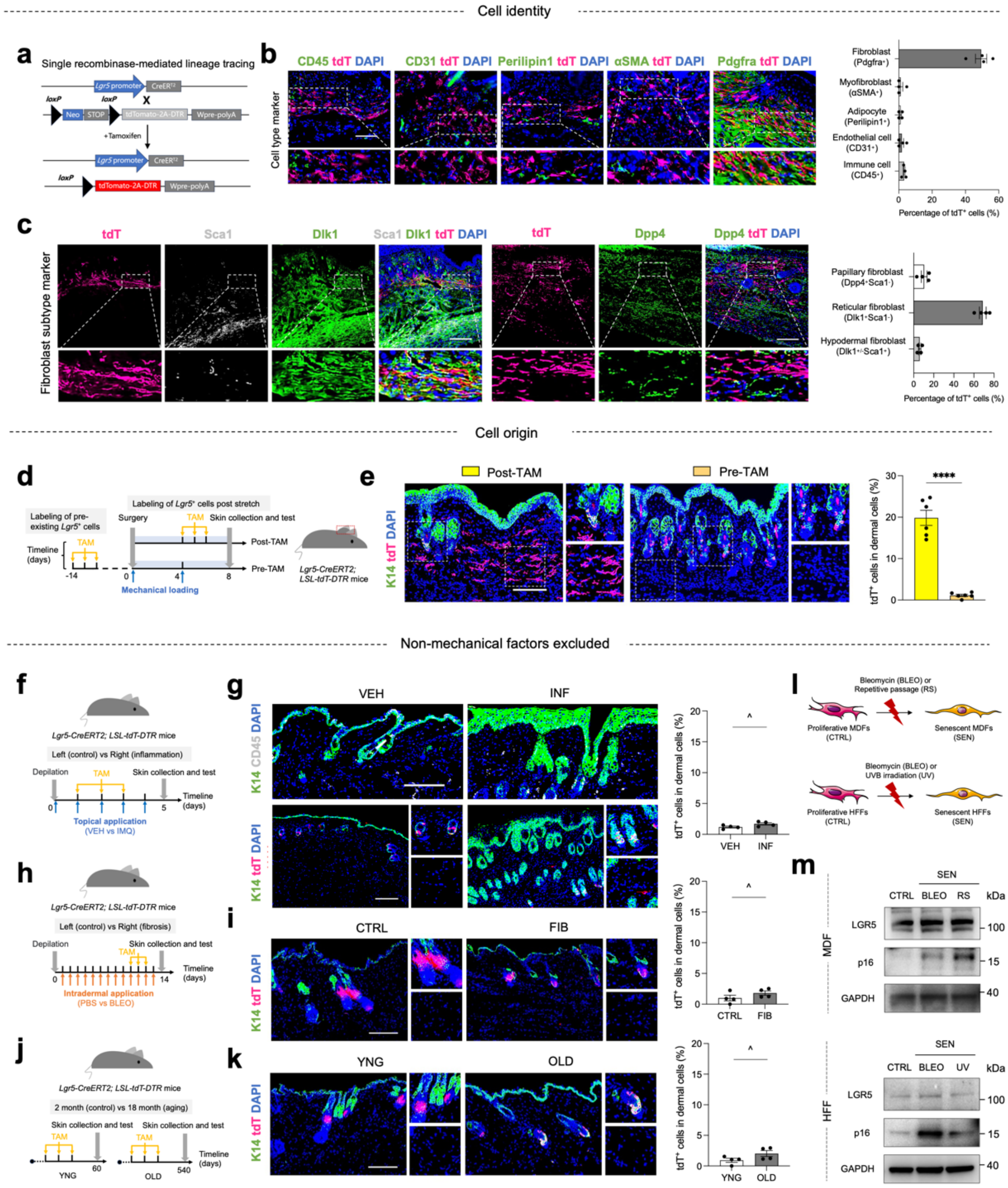

Supplementary Figure 3. *Lgr5*$^{+}$ MIRFs represent *de novo*-induced reticular fibroblasts

a, Schematic of the lineage tracing strategy in *Lgr5*-tracing mice.

b, Immunofluorescence co-staining for tdT and markers (CD45, CD31, Perilipin1, αSMA, Pdgfra) in expanded skin (n = 4 mice). Left, representative images (scale bar, 100 μm); right, quantification of tdT^{+} cells within each cell type.

c, Immunofluorescence staining for tdT with Sca1 and Dlk1 (left) or Dpp4 (middle) in expanded skin (n = 4 mice). Left and middle, representative images (scale bar, 100 μm); right, quantification of tdT^{+} cells within each fibroblast subtype.

d, Schematic timeline for tracing the origin of *Lgr5*$^{+}$ MIRFs by administering tamoxifen before (pre-TAM) or after (post-TAM) mechanical loading.

e, Immunofluorescence staining for K14 and tdT in expanded skin (n = 6 mice). Left,

representative images (scale bar, 100 μm); right, quantification of tdT^+ cells (****$P < 0.0001$).

f-g, Tracing *Lgr5*$^+$ cells in mice with imiquimod (IMQ)-induced skin inflammation (INF). Vehicle (VEH)-treated mice served as controls. (f) Schematic timeline. (g) Immunofluorescence staining for K14 with CD45 or tdT (n = 4 mice). Left, representative images (scale bar, 100 μm); right, quantification of tdT^+ cells (^$P = 0.0743$).

h-i, Tracing *Lgr5*$^+$ cells in mice with bleomycin (BLEO)-induced skin fibrosis (FIB). PBS-treated mice served as controls (CTRL). (h) Schematic timeline. (i) Immunofluorescence staining for K14 and tdT (n = 4 mice). Left, representative images (scale bar, 100 μm); right, quantification of tdT^+ cells (^$P = 0.2111$).

j-k, Tracing *Lgr5*$^+$ cells in young (YNG) and old (OLD) mice. (j) Schematic timeline. (k) Immunofluorescence staining for K14 and tdT (n = 4 mice). Left, representative images (scale bar, 100 μm); right, quantification of tdT^+ cells (^$P = 0.1092$).

l, Timeline for senescence induction in mouse dermal fibroblasts (MDFs) and human foreskin fibroblasts (HFFs). BLEO, bleomycin-induced senescence; RS, replicative senescence; UV, UVB-induced senescence. Images provided by Servier Medical Art (https://smart.servier.com), licensed under CC BY 4.0 (https://creativecommons.org/licenses/by/4.0/).

m, Western blot of LGR5 and p16 in proliferative (CTRL) versus senescent (SEN) MDFs (upper) and HFFs (lower).

Data are presented as mean ± SEM. Statistical significance was determined by two-tailed unpaired Student's t-test. Source data are provided as a Source Data file.

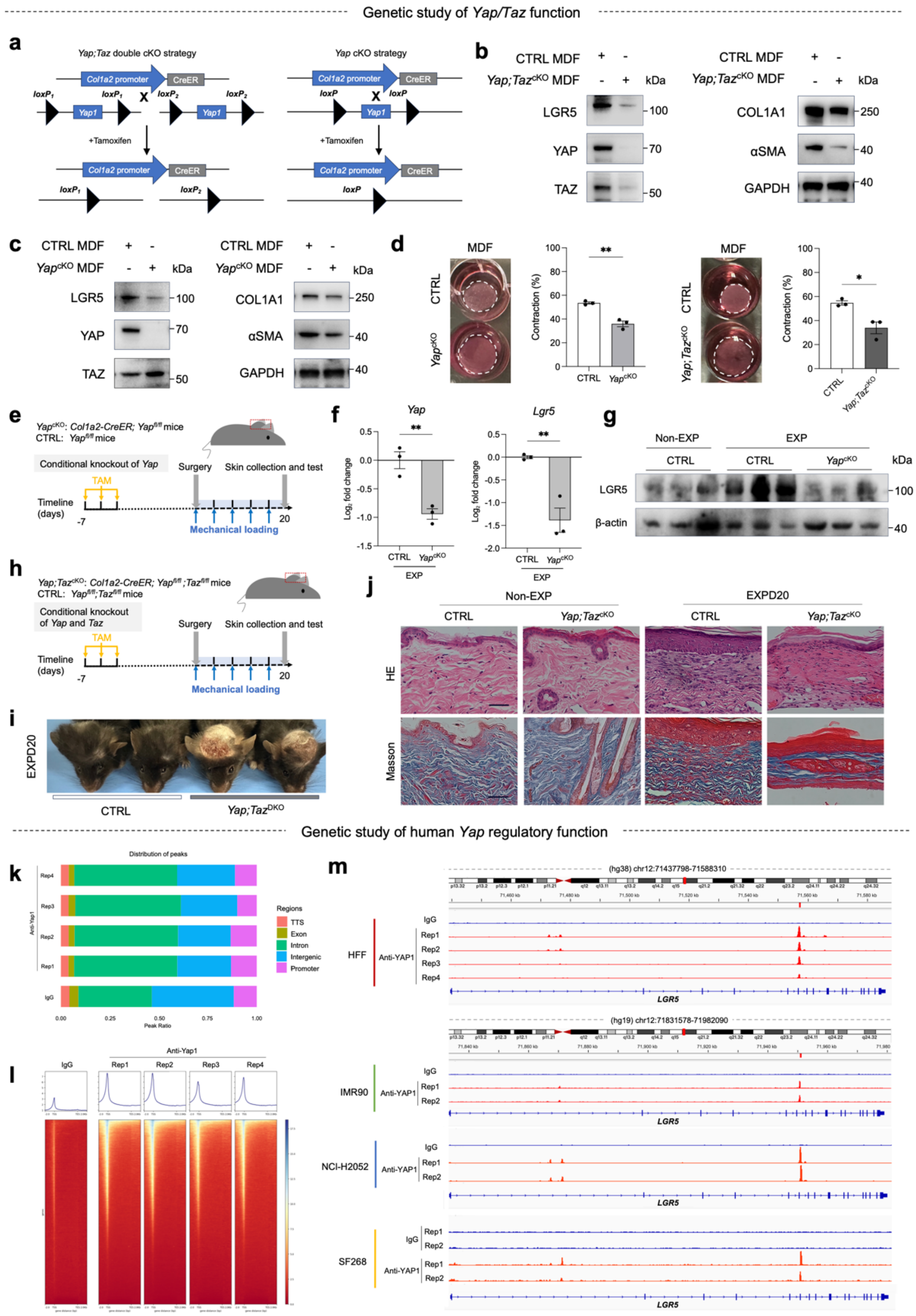

Supplementary Figure 4. Yap/Taz induce *Lgr5* expression while promoting matrix deposition and contraction in fibroblasts

a, Schematic of the conditional knockout strategy of *Yap* and *Taz*. Left, double knockout of *Yap* and *Taz*; right, single knockout of *Yap*.

b, Western blot of LGR5, YAP, TAZ, COL1A1 and αSMA in control versus *Yap;Taz*^{cKO} MDFs.

c, Western blot of LGR5, YAP, TAZ, COL1A1 and αSMA in control versus *Yap*^{cKO} MDFs.

d, Matrix contraction assay mediated by control versus *Yap;Taz*^{cKO} MDFs (left), and control versus *Yap*^{cKO} MDFs (right) (n = 3 biological replicates). For each panel, left: representative images of collagen gels; right: quantification of contraction percentage (**P* = 0.0162, ***P* = 0.0024).

e, Schematic timeline for assessing *Lgr5* expression in *Yap*^{cKO} mice and their littermate controls following skin expansion.

f, qPCR analysis of *Yap* and *Lgr5* mRNA in the expanded dermis of indicated mice (n = 3 mice) (***P* = 0.0056 / 0.0067).

g, Western blot of *Lgr5* in non-expanded and expanded dermis from control versus *Yap*^{cKO} mice (n = 3 mice).

h, Schematic timeline for assessing mechanoadaptation in *Yap;Taz*^{cKO} mice and their littermate controls following skin expansion.

i, Representative images of the expanded region in control versus *Yap;Taz*^{cKO} mice.

j, Representative images of H&E staining and Masson's trichrome staining of skin from control versus *Yap;Taz*^{cKO} mice (scale bar, 100 μm).

k, Percentage bar chart showing the distribution ratios of YAP-binding peaks across distinct genomic regions, derived from CUT&RUN sequencing data of HFFs.

l, TSS enrichment analysis based on CUT&RUN sequencing data of HFFs.

m, Genome browser tracks displaying sequencing signals at the LGR5 locus in HFF, IMR90, NCI-H2052, and SF268 cells.

Data are presented as mean ± SEM. Statistical significance was determined by two-tailed unpaired Student's t-test. Source data are provided as a Source Data file.

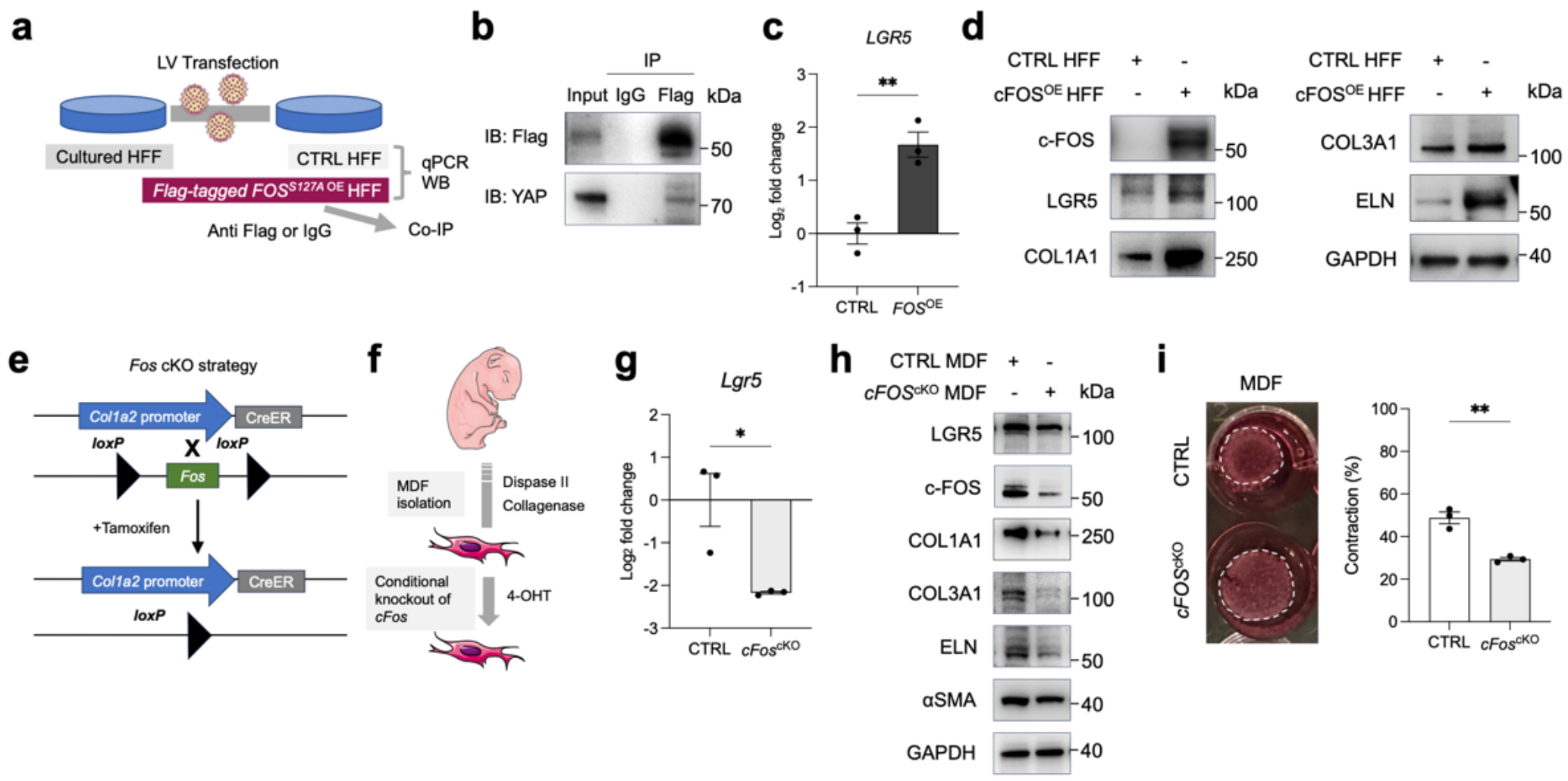

Supplementary Figure 5. c-FOS induces *Lgr5* expression while promoting matrix deposition and contraction in fibroblasts

a, Schematic workflow for the construction of control and Flag-tagged *FOS*^{OE} HFFs for Co-IP, qPCR and WB analysis.

b, Co-IP analysis of Flag-tagged c-FOS with YAP. Lysates from *FOS*^{OE} HFFs were subjected to IP using an anti-Flag antibody, followed by IB with antibodies against YAP and Flag.

c, qPCR analysis of *LGR5* mRNA in control versus *FOS*^{OE} HFFs (n = 3 biological replicates).

d, Western blot of LGR5, c-FOS, COL1A1, COL3A1 and ELN in control versus *FOS*^{OE} HFFs.

e, Schematic of the conditional knockout strategy of *Fos*.

f, Schematic workflow for the isolation and induction of *Fos*^{cKO} MDFs. Images provided by Servier Medical Art (https://smart.servier.com), licensed under CC BY 4.0 (https://creativecommons.org/licenses/by/4.0/).

g, qPCR analysis of *Lgr5* mRNA in control versus *Fos*^{cKO} MDFs (n = 3 biological replicates) (**P* = 0.0248).

h, Western blot of LGR5, c-FOS, COL1A1, COL3A1 and ELN in control versus *Fos*^{cKO} MDFs.

i, Matrix contraction assay (n = 3 biological replicates). Left, representative images of collagen gels; right, quantification of contraction percentage (***P* = 0.0024).

Data are presented as mean ± SEM. Statistical significance was determined by two-tailed unpaired Student's t-test. Source data are provided as a Source Data file.

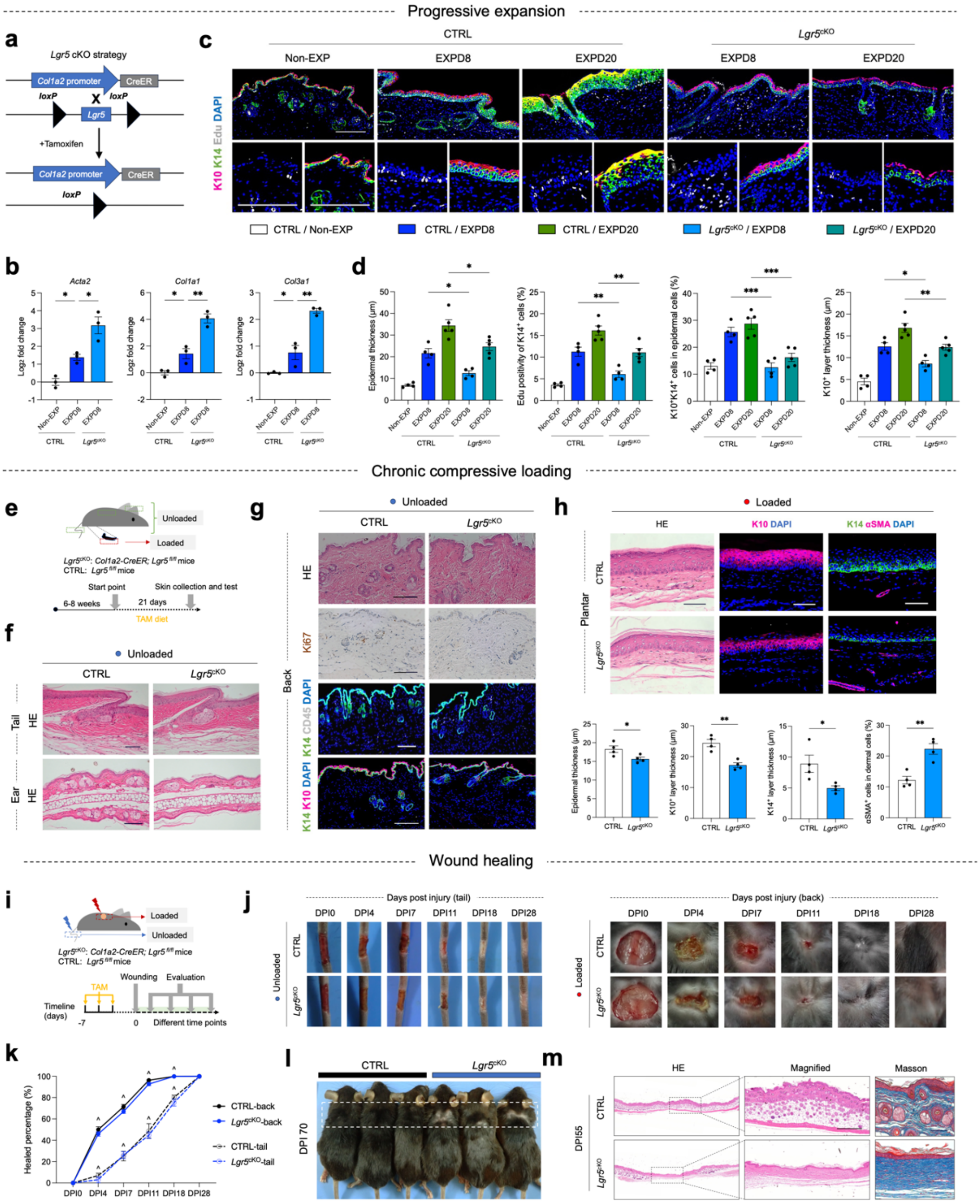

Supplementary Figure 6. *Lgr5* deficiency hinders mechanoadaptation of the skin under diverse mechanical stress

a, Schematic of the conditional *Lgr5* knockout strategy.

b, qPCR analysis of dermal *Acta2* (**P* = 0.0453 / 0.0151), *Col1a1* (**P* = 0.0378, ***P* = 0.0022), and *Col3a1* (**P* = 0.0384, ***P* = 0.0012) mRNA (n = 3 mice).

c-d, Immunofluorescence staining for EdU, K14 and K10 (*n* = 4–5 mice). (c) Representative images (scale bar, 100 μm). (d) Quantification of epidermal thickness (**P* = 0.0118 / 0.0375), EdU positivity of K14$^+$ cells (***P* = 0.0045 / 0.0095), the percentage of K10$^+$K14$^+$ cells (****P* = 0.0003 / 0.0007), and K10$^+$ layer thickness (**P* = 0.0405, ***P* = 0.0077).

e, Schematic timeline for assessing mechanoadaptation under homeostasis.

f, H&E staining of tail and ear skin (scale bar, 50 μm).

g, Analysis of tail and ear skin by H&E, Ki67 IHC, immunofluorescence staining for K14 and CD45 or K14 and K10 (scale bar, 100 μm).

h, Analysis of plantar skin by H&E and immunofluorescence staining for K10 or K14 and αSMA (n = 4 mice). Upper, representative images (scale bar, 50 μm). Lower, quantification of epidermal thickness (*P = 0.0398), $K10^+$ layer thickness (**P = 0.0023), $K14^+$ layer thickness (*P = 0.0345), and the percentage of $αSMA^+$ cells (**P = 0.0031).

i-k, Assessment of mechanoadaptation during wound healing. (i) Schematic timeline. (j) Representative images. Left, wounded back skin; right, wounded tail skin. (k) Quantification. Solid line, back skin (n = 10–11 mice) (^P = 0.8418 / 0.5723 / 0.1937 / 0.9967); dashed line, tail skin (n = 5–6 mice) (^P = 0.8701 / 0.9999 / 0.9964 / 0.7933). DPI, days post injury.

l, Representative images of back skin at DPI70. The wound area is outlined with a white dashed line.

m, H&E staining and Masson's trichrome staining at DPI55 (scale bar, 500 μm for HE, 100 μm for Masson).

Data are presented as mean ± SEM. For dataset (k), statistical significance was determined by two-way ANOVA with Šidák's multiple comparison test. Otherwise, statistical significance was determined by two-tailed unpaired Student's t-test or one-way ANOVA with Tukey's post hoc test. Source data are provided as a Source Data file.

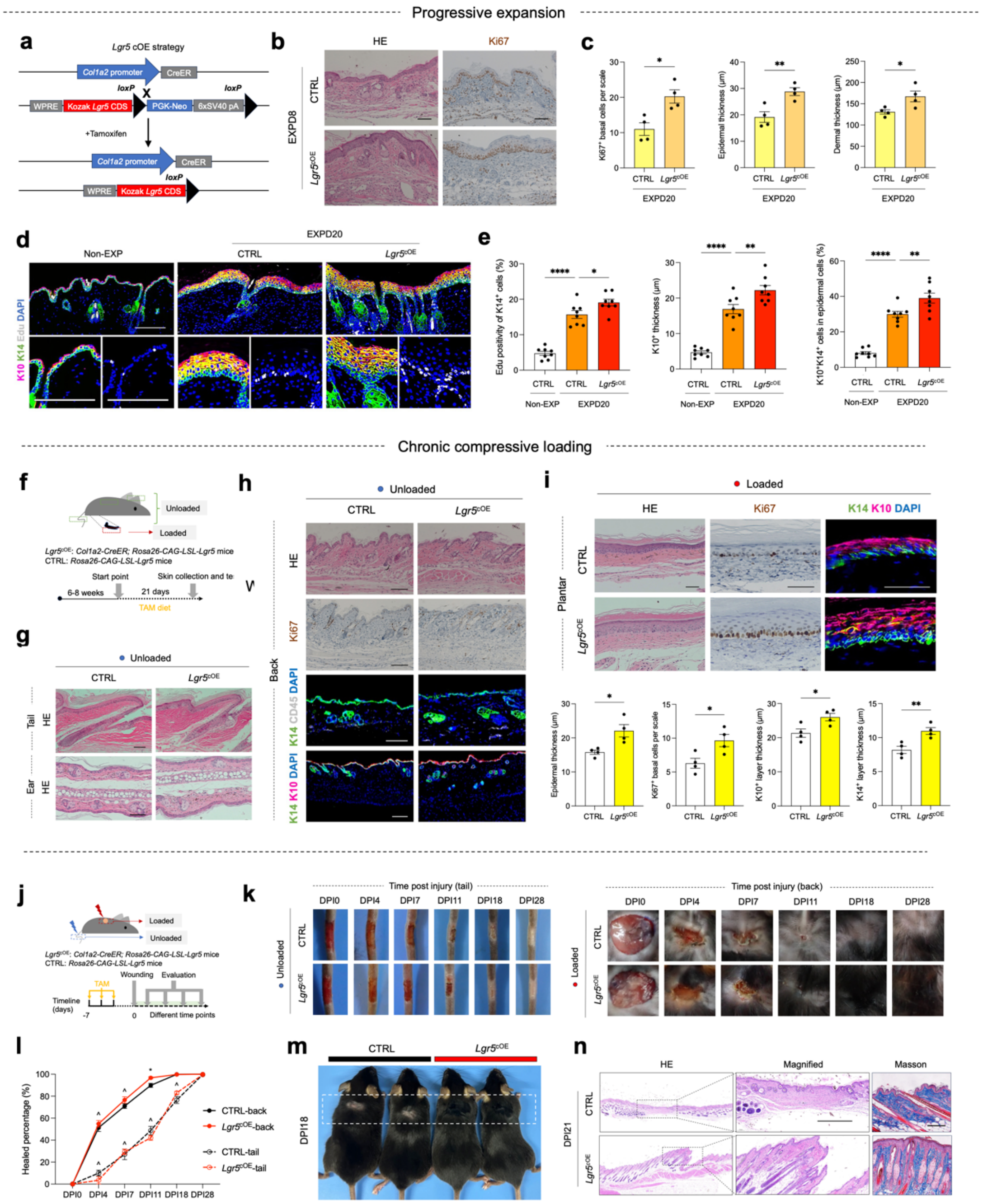

Supplementary Figure 7. Conditional *Lgr5* overexpression promotes skin mechanoadaptation

a, Schematic of the conditional *Lgr5* overexpression strategy.

b-c, H&E and Ki67 IHC staining (n = 4 mice). (b) Representative images (scale bar, 100 μm). (c) Quantification of number of $Ki67^+$ basal cells per scale (**P* = 0.0117), epidermal thickness (***P* = 0.0084) and dermal thickness (**P* = 0.0371).

d-e, Immunofluorescence staining for EdU, K14 and K10 (n = 8 mice). (d) Representative images (scale bar, 100 μm). (e) Quantification of EdU positivity of $K14^+$ cells (**P* = 0.0393, *****P* < 0.0001), $K10^+$ layer thickness (***P* = 0.0056, *****P* < 0.0001) and the percentage of $K10^+K14^+$ cells (***P* = 0.0091, *****P* < 0.0001).

f, Schematic timeline for assessing mechanoadaptation under homeostasis.
g, Analysis of tail and ear skin by H&E staining (scale bar, 50 μm).
h, Analysis of tail and ear skin by H&E, Ki67 IHC, and immunofluorescence staining for K14 and K10 (scale bar, 100 μm).
i, Analysis of plantar skin by H&E, Ki67 IHC, and immunofluorescence staining for K10 and K14 (n = 4 mice). Upper, representative images (scale bar, 50 μm). Lower, quantification of epidermal thickness (**P* = 0.0173), $Ki67^+$ basal cells per scale (**P* = 0.0280), $K10^+$ layer thickness (**P* = 0.0289), and $K14^+$ layer thickness (***P* = 0.0090).
j-l, Assessment of mechanoadaptation during wound healing. (j) Schematic timeline. (k) Representative images. Left, wounded back skin; right, wounded tail skin. (l) Quantification. Solid line, back skin (n = 10 mice) (^*P* = 0.9729 / 0.5983, **P* = 0.0278); dashed line, tail skin (n = 5 mice) (^*P* = 0.6667 / 0.9989 / 0.7206 / 0.5278). DPI, days post injury.
m, Representative images of back skin at DPI18. The wound area is outlined with a white dashed line.
n, H&E and Masson's trichrome staining at DPI21 (scale bar, 500 μm for H&E, 100 μm for Masson).
Data are presented as mean ± SEM. For dataset (l), statistical significance was determined by mixed-effects analysis with Šidák's multiple comparison test. Otherwise, statistical significance was determined by two-tailed unpaired Student's t-test or one-way ANOVA with Tukey's post hoc test. Source data are provided as a Source Data file.

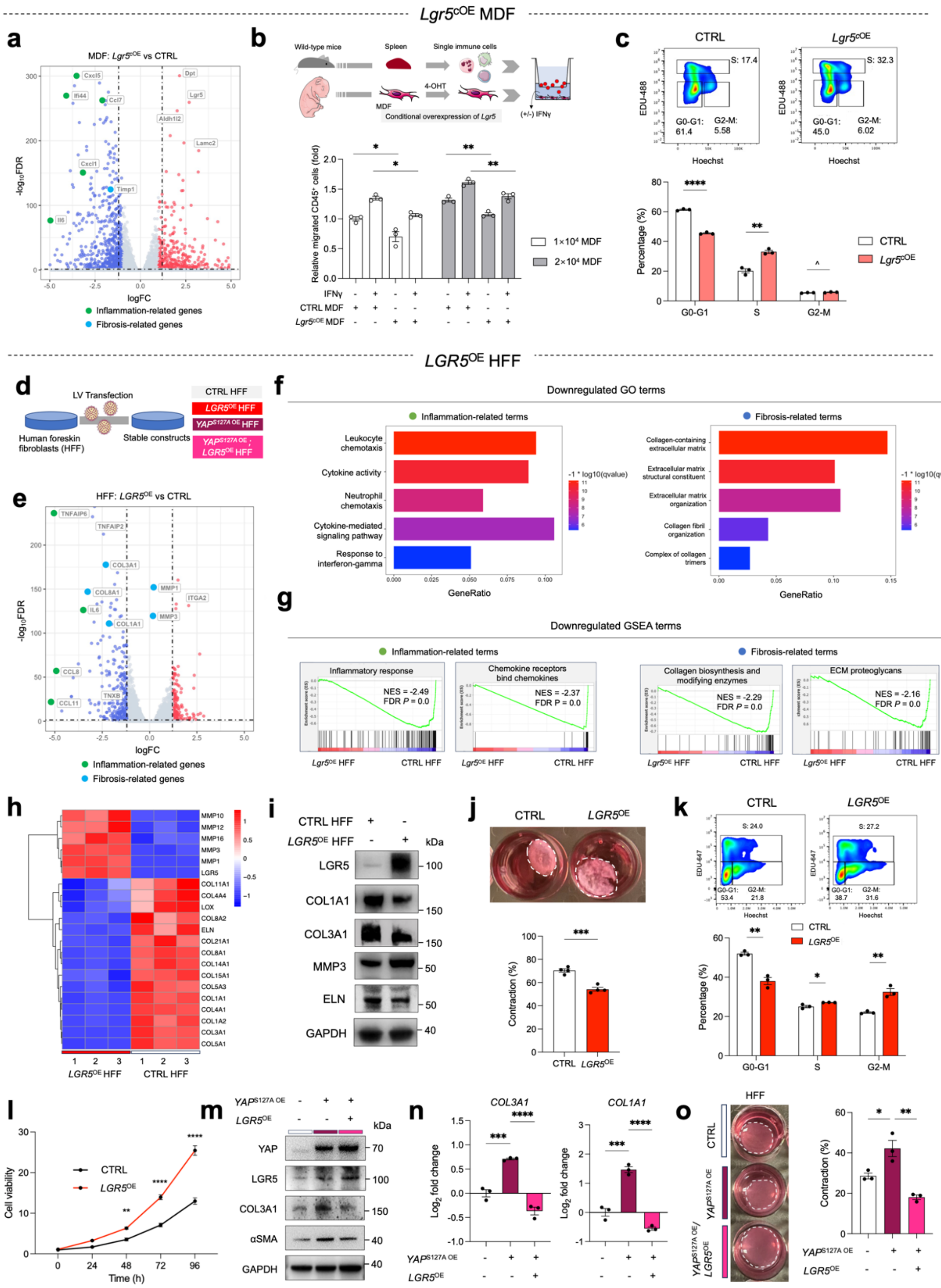

Supplementary Figure 8. *Lgr5* overexpression drives an anti-inflammatory, anti-fibrotic, and proliferative program in dermal fibroblasts across species

a, Volcano plot derived from bulk RNA-seq data, showing differentially expressed genes in *Lgr5*^{cOE} MDFs versus CTRL MDFs with several key genes highlighted.

b, Transwell assay assessing immune cell migration toward MDFs. Upper, schematic workflow. Lower, quantification of migrated CD45^{+} cells, with or without IFNγ stimulation (n = 3 biological replicates) (**P* = 0.0121 / 0.0152, ***P* = 0.0046 / 0.0058). Images provided

by Servier Medical Art (https://smart.servier.com), licensed under CC BY 4.0 (https://creativecommons.org/licenses/by/4.0/).

c, Cell-cycle analysis following EdU incorporation. Upper, representative plots (n = 3 biological replicates). Lower, quantification of cells in each cell-cycle phase (^*P* = 0.1646, ***P* = 0.0020, *****P* < 0.0001).

d, Schematic workflow for the construction of CTRL, *LGR5*^{OE}, *YAP*$^{S127A\ OE}$, and *YAP*$^{S127A\ OE}$; *LGR5*^{OE} HFFs. Images provided by Servier Medical Art (https://smart.servier.com), licensed under CC BY 4.0 (https://creativecommons.org/licenses/by/4.0/).

e, Volcano plot showing differentially expressed genes with several key genes highlighted.

f, Bar plots showing the significantly downregulated GO terms for inflammation (left) and fibrosis (right) in *LGR5*^{OE} HFFs.

g, GSEA plots showing the significantly downregulated gene sets for inflammation (left) and fibrosis (right) in *LGR5*^{OE} HFFs.

h, Heatmap of key extracellular-matrix proteins.

i, Western blot of LGR5, COL1A1, COL3A1, MMP3 and ELN.

j, Matrix contraction assay (n = 4 biological replicates). Upper, representative images of collagen gels; lower, quantification of contraction percentage (****P* = 0.0003).

k, Cell-cycle analysis following EdU incorporation (n = 3 biological replicates). Upper, representative plots. Lower, quantification of cells in each cell-cycle phase (**P* = 0.0272, ***P* = 0.0020 / 0.0039).

l, Representative CCK-8 cell growth curve (n = 8 technical replicates, representative of three independent experiments) (**P = 0.0019, ****P < 0.0001).

m, Western blot of LGR5, YAP, COL3A1 and αSMA.

n, qPCR analysis of *COL3A1* and *COL1A1* mRNA (n = 3 biological replicates) (****P* = 0.0005 / 0.0001, *****P* < 0.0001).

o, Matrix contraction assay (n = 3 biological replicates). Left, representative images; right, quantification of contraction percentage (**P* = 0.0219, ***P* = 0.0013).

Data are presented as mean ± SEM. For dataset (l), statistical significance was determined by two-way ANOVA with Šidák's multiple comparison test. Otherwise, statistical significance was determined by two-tailed unpaired Student's t-test or one-way ANOVA with Tukey’s post hoc test. Source data are provided as a Source Data file.

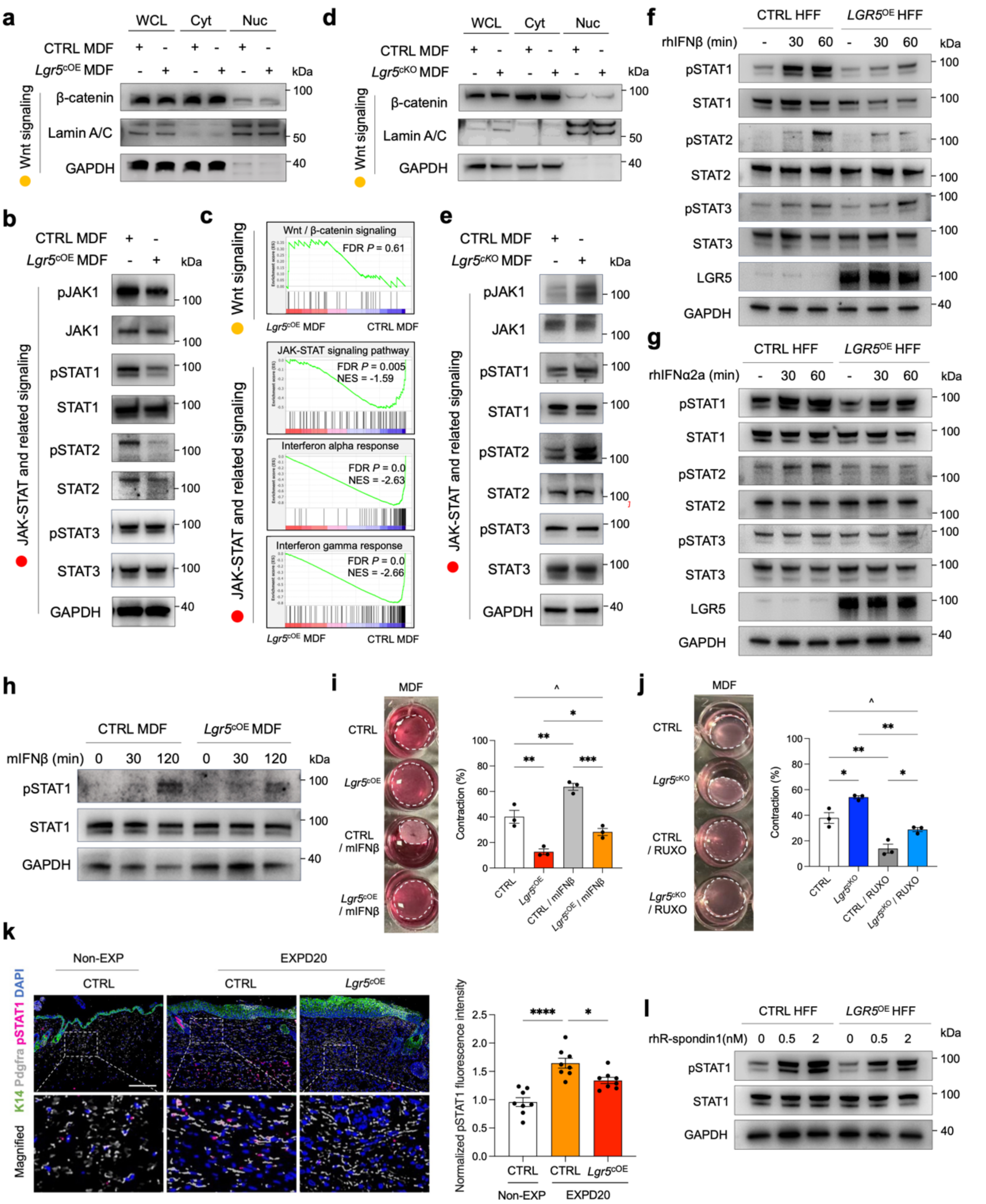

Supplementary Figure 9. LGR5 inhibits the JAK-STAT pathway across human and mouse systems

a, Western blot of β-catenin expression and distribution in cytoplasmic and nuclear fractions in control and *LGR5*^{cOE} MDFs. GAPDH and Lamin A/C served as loading controls.

b, Western blot of JAK-STAT pathway components in control and *Lgr5*^{cOE} MDFs.

c, GSEA plots showing enrichment of Wnt signaling and JAK-STAT or related signaling gene sets in *Lgr5*^{cOE} MDFs versus control MDFs.

d, Western blot of β-catenin expression and distribution in cytoplasmic and nuclear

fractions of control and *LGR5*^{cKO} MDFs. GAPDH and Lamin A/C served as loading controls.
e, Western blot of JAK-STAT pathway components in control and *Lgr5*^{cKO} MDFs.
f-g, Western blot of time-dependent activation of the JAK-STAT pathway in control and *LGR5*^{OE} HFFs with or without rhIFNβ (f) or rhIFNα2a (g) stimulation.
h, Western blot of time-dependent STAT1 activation in *Lgr5*^{cOE} and CTRL MDFs with or without mIFNβ stimulation.
i, Matrix contraction assay mediated by *Lgr5*^{cOE} and CTRL MDFs, treated with or without mIFNβ stimulation (n = 3 biological replicates). Left, representative images of collagen gels; right, quantification of contraction percentage (^*P* = 0.1372, **P* = 0.0455, ***P* = 0.0051 / 0.0019, ****P* = 0.0003).
j, Matrix contraction assay mediated by *Lgr5*^{cKO} and CTRL MDFs, treated with or without ruxolitinib (RUXO) (n = 3 biological replicates). Left, representative images of collagen gels; right, quantification of contraction percentage (^*P* = 0.1997, **P* = 0.0175 / 0.0276, ***P* = 0.0012 / 0.0017).
k, Immunofluorescence staining for Pdgfra, K14 and pSTAT1 in the skin of indicated mice (n = 8 mice). Left, representative images (scale bar, 100 μm); right, quantification of pSTAT1 fluorescence intensity (**P* = 0.0230, *****P* < 0.0001).
l, Western blot of concentration-dependent activation of the JAK-STAT pathway in control and *LGR5*^{OE} HFFs with or without R-spondin1.
Data are presented as mean ± SEM. Statistical significance was determined by one-way ANOVA with Tukey's post hoc test. Source data are provided as a Source Data file.

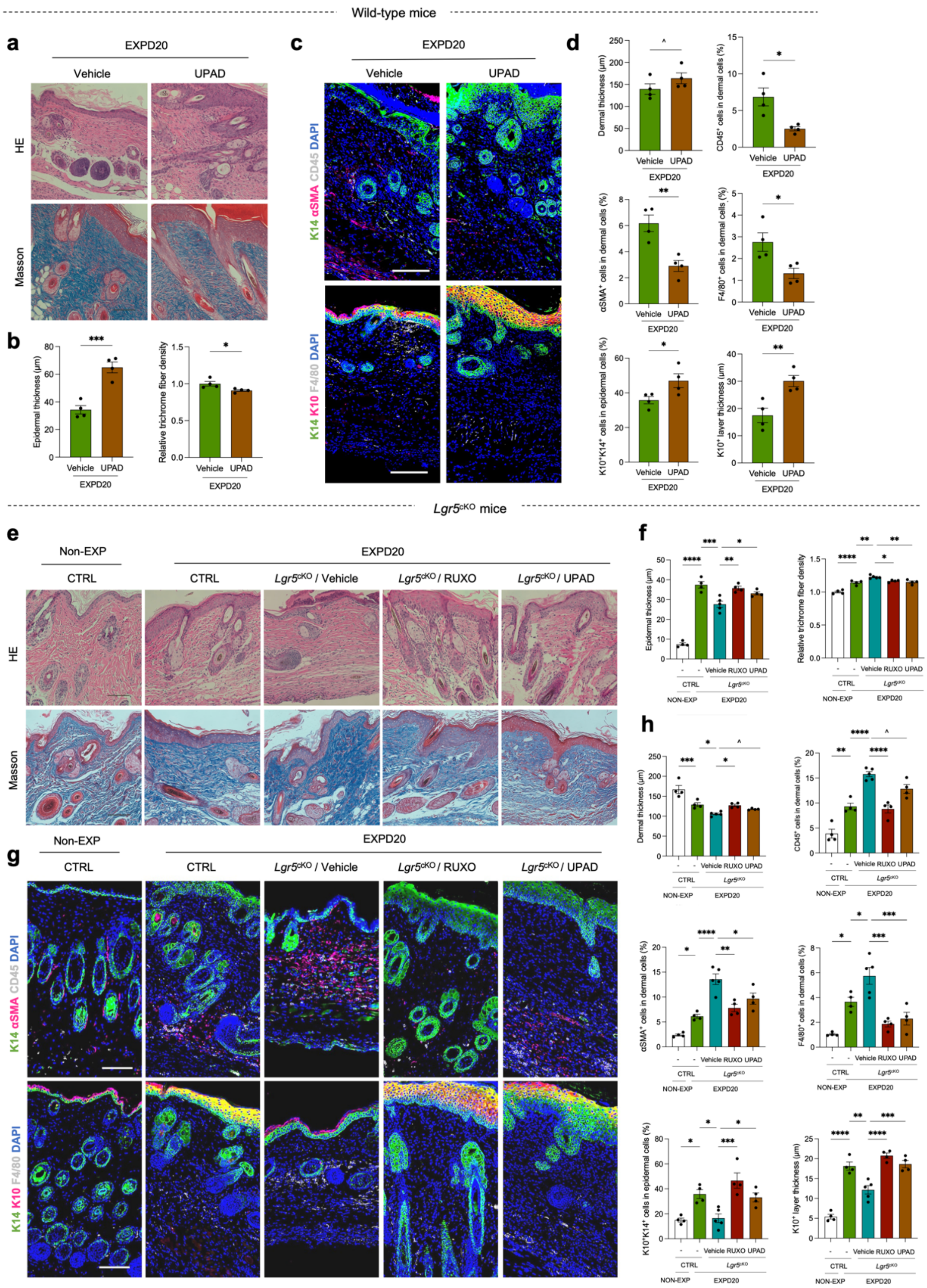

Supplementary Figure 10. Topical JAK1 inhibition accelerates full-thickness skin regeneration under mechanical stress

a-b, Histological analysis of the expanded skin of indicated mice by H&E staining and Masson's trichrome staining (n = 4 mice). (a) Representative images (scale bar, 100 μm). (b) Quantification of epidermal thickness (****P* = 0.0008) and relative trichrome density (**P* = 0.0340).

c-d, Immunofluorescence analysis of expanded skin of indicated mice (n = 4 mice). (c) Upper panels, representative images of K14, αSMA and CD45; lower panels, representative images of K14, K10 and F4/80; scale bar, 100 μm. (d) Quantification of the following parameters: dermal thickness (^*P* = 0.2055); the percentage of $CD45^+$ cells (**P* = 0.0136), $αSMA^+$ cells (***P* = 0.0051), and $F4/80^+$ cells (**P* = 0.0255); the percentage of $K10^+K14^+$ cells (**P* = 0.0497); and $K10^+$ layer thickness (***P* = 0.0092).
e-f, Histological analysis of the expanded skin of indicated mice by H&E staining and Masson's trichrome staining (n = 4–5 mice). (e) Representative images (scale bar, 100 μm). (f) Quantification of epidermal thickness (**P* = 0.0433, ***P* = 0.0021, ****P* = 0.0003, *****P* < 0.0001) and relative trichrome density (**P* = 0.0350, ***P* = 0.0028 / 0.0070, *****P* < 0.0001).
g-h, Immunofluorescence analysis of expanded skin of indicated mice (n = 4–5 mice). (g) Upper panels, representative images of K14, αSMA and CD45; lower panels, representative images of K14, K10 and F4/80; scale bar, 100 μm. (h) Quantification of the following parameters: dermal thickness (^*P* = 0.4686, **P* = 0.0302 / 0.0472, ****P* = 0.0008); the percentage of $CD45^+$ cells (^*P* = 0.0710, ***P* = 0.0010, *****P* < 0.0001), $αSMA^+$ cells (**P* = 0.0477 / 0.0269, ***P* = 0.0011, *****P* < 0.0001), and $F4/80^+$ cells (**P* = 0.0103 / 0.0342, ****P* = 0.0001 / 0.0005); the percentage of $K10^+K14^+$ cells (**P* = 0.0153 / 0.0178 / 0.0469, ****P* = 0.0003); and $K10^+$ layer thickness (***P* = 0.0015, *****P* < 0.0001).
Data are presented as mean ± SEM. Statistical significance was determined by two-tailed unpaired Student's t-test or one-way ANOVA with Tukey's post hoc test. Source data are provided as a Source Data file.

Supplementary Methods

- **Flow cytometry gating strategies**

1. *In vivo* dermal cells gating strategies

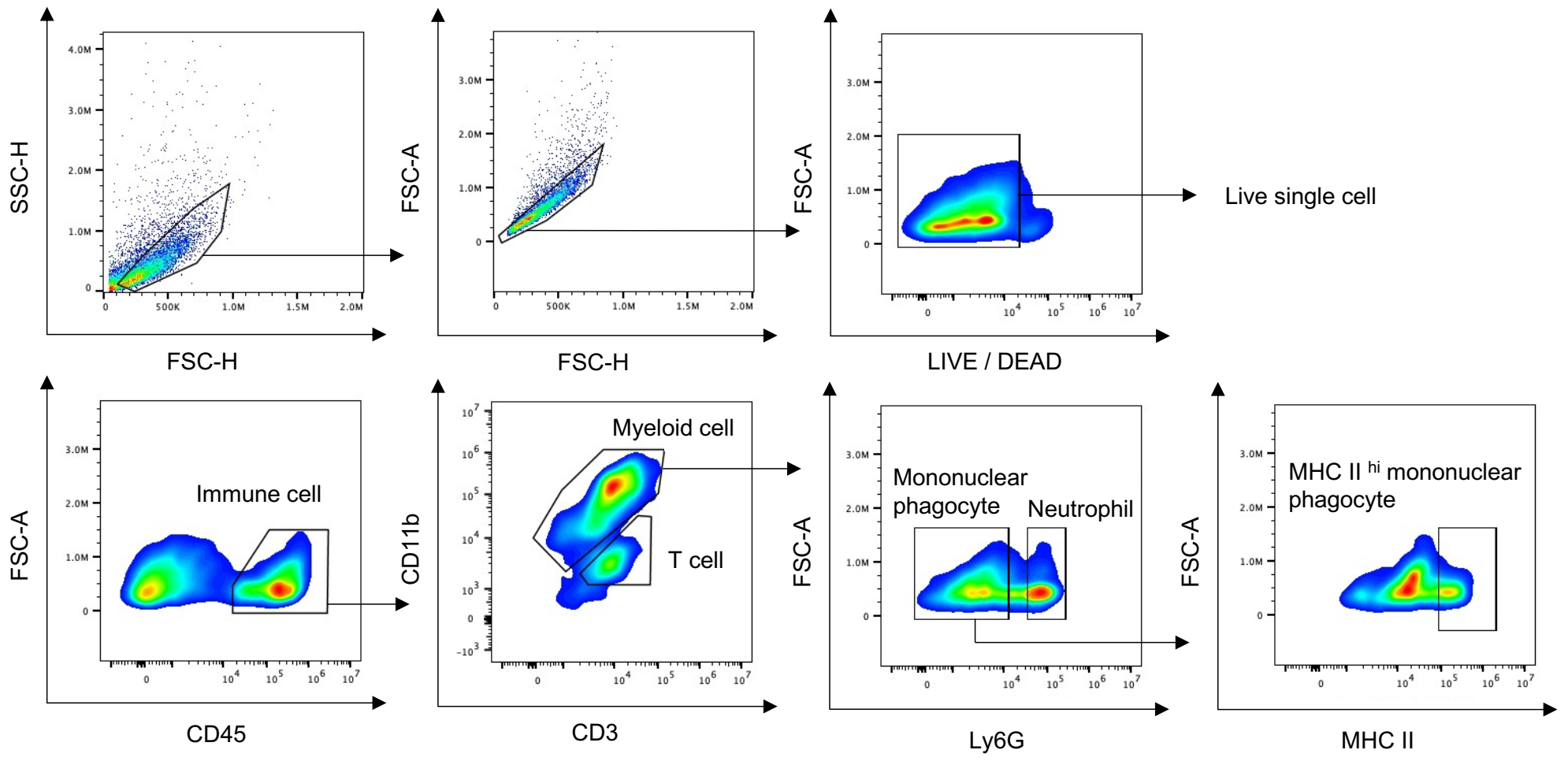

2. *In vitro* immune cells gating strategies

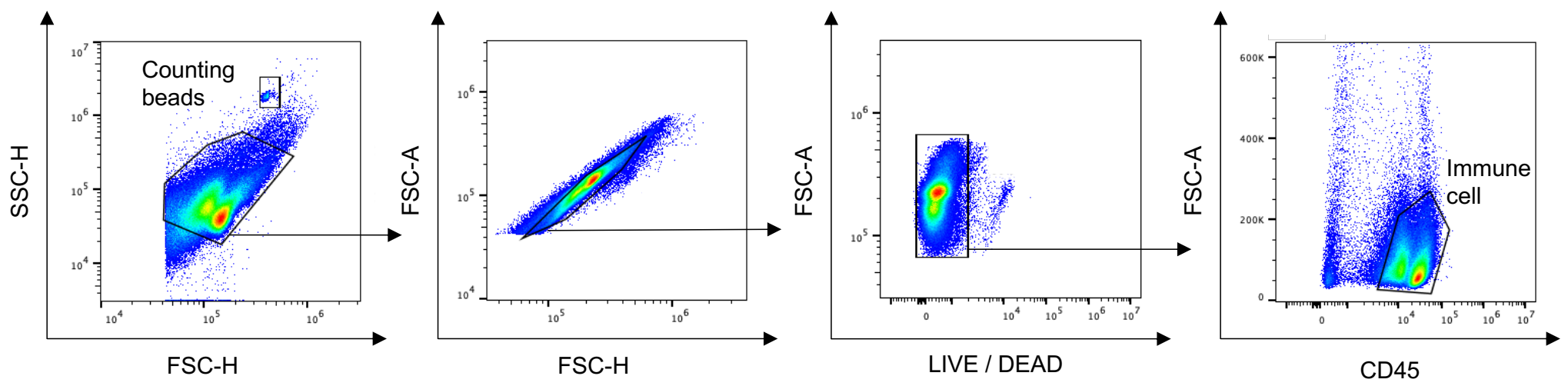

3. *In vitro* EdU incorporation assay gating strategies

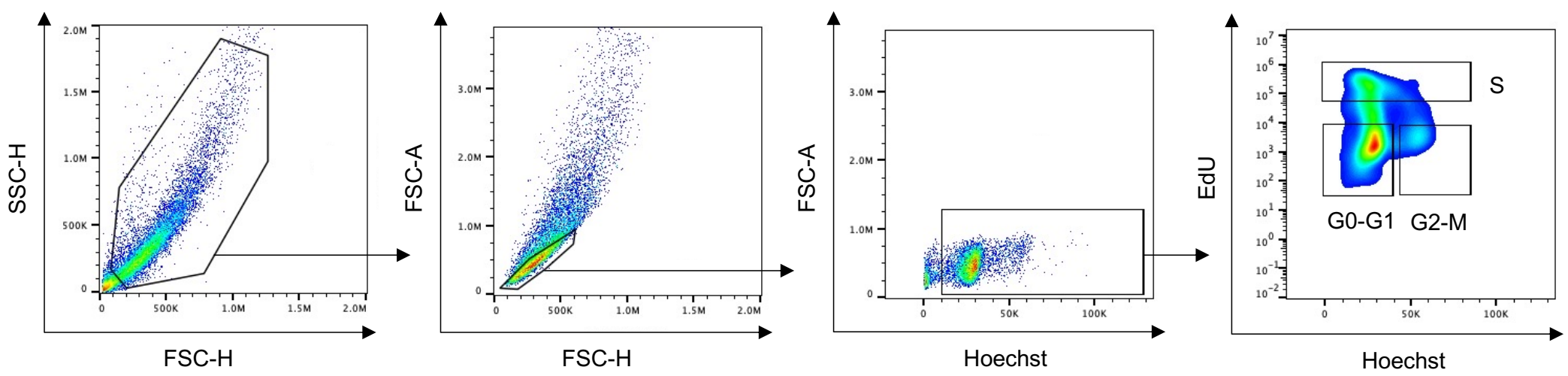

Uncropped blots for Supplementary Figures

Supplementary Figure 3m
(The blue dashed box indicates data for MDF,
and the red dashed box indicates data for HFF.)

p16

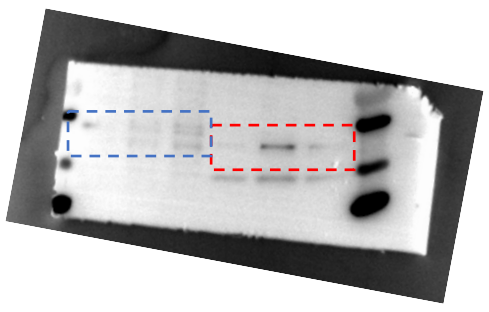

LGR5

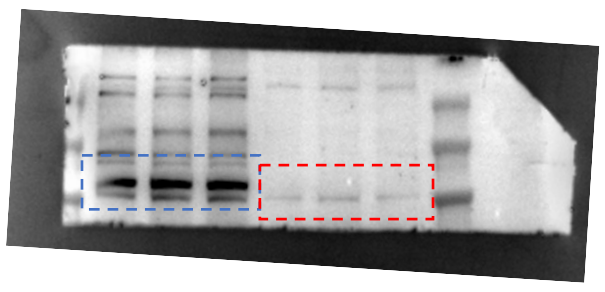

GAPDH

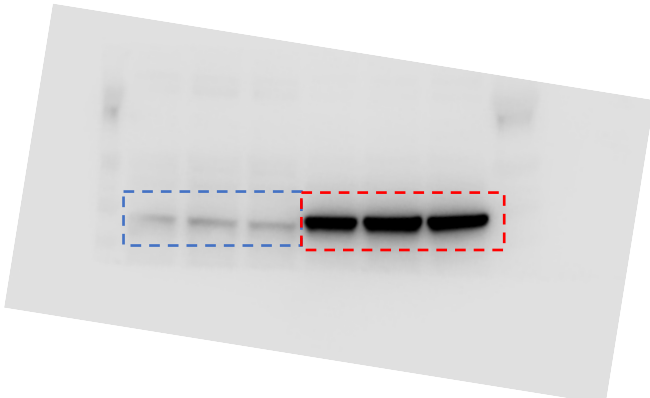

Supplementary Figure 4b, 4c
(The blue dashed box indicates 4b, and the red dashed box indicates 4c.)

LGR5 (different exposure time of the same band)

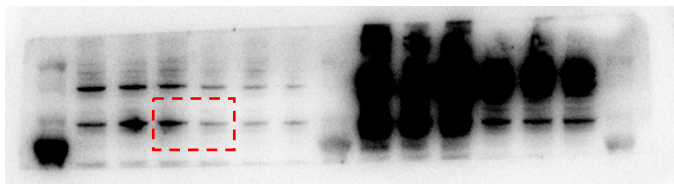

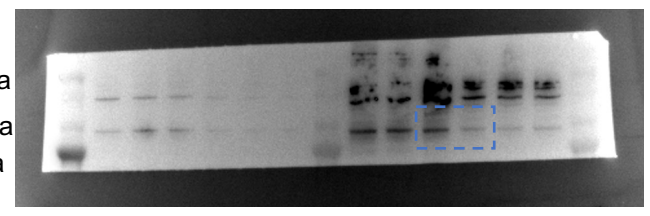

YAP

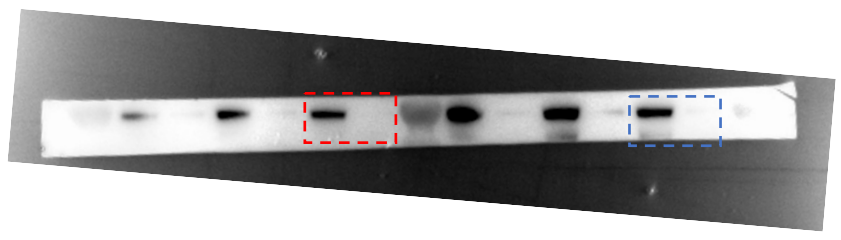

TAZ

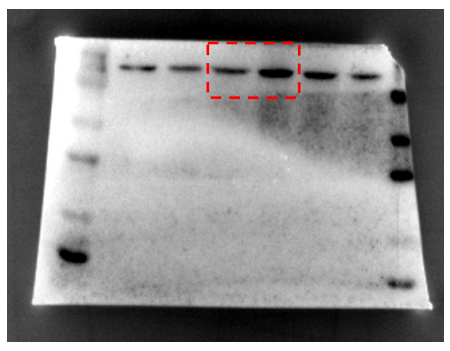

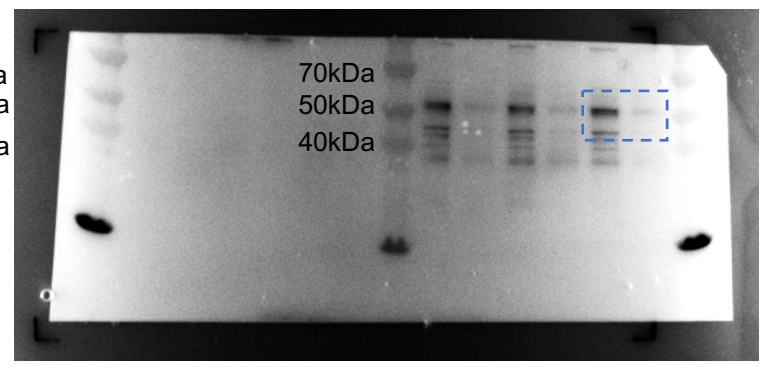

Col1a1

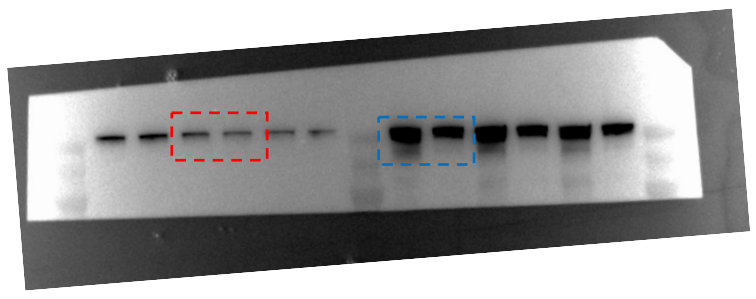

αSMA

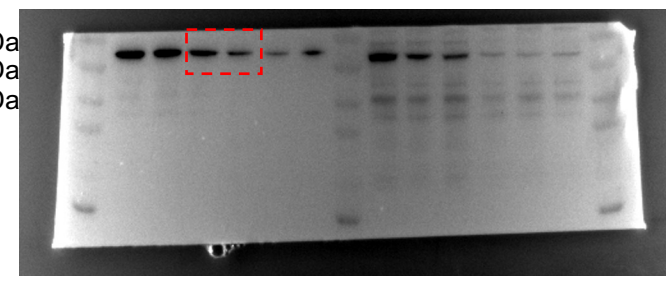

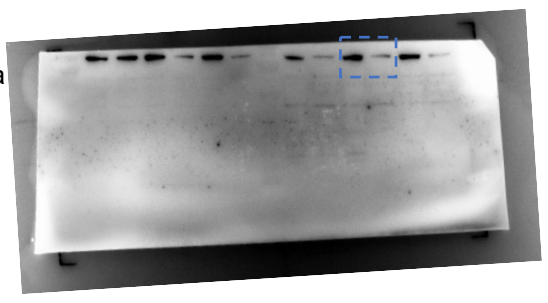

GAPDH

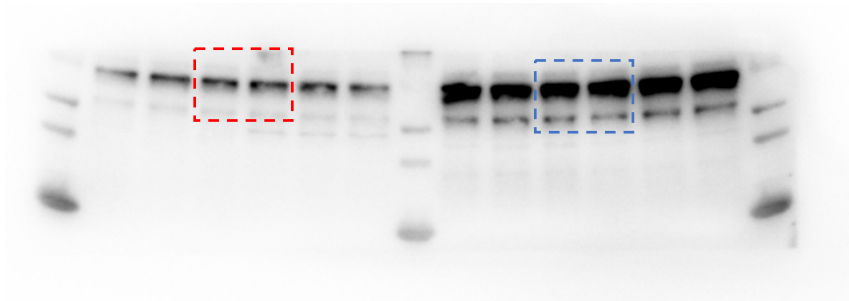

Supplementary Figure 4g

LGR5

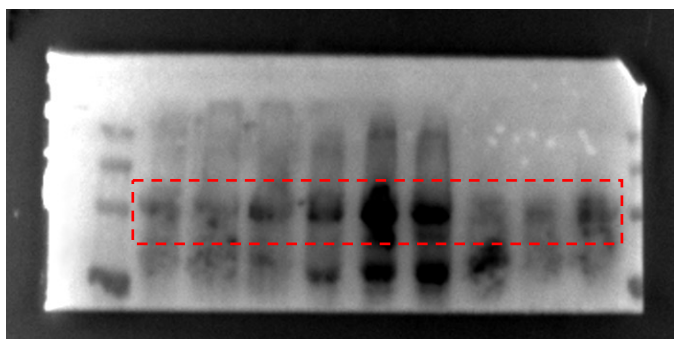

β-actin

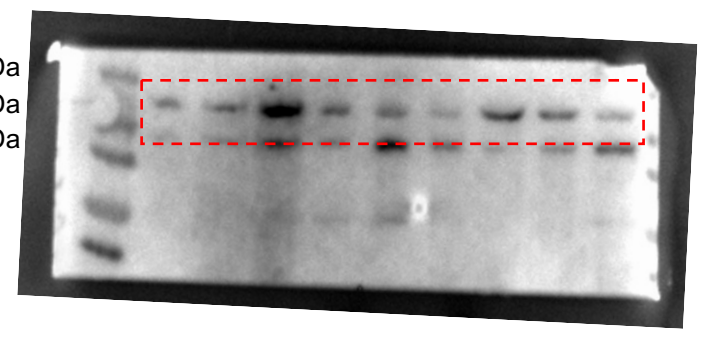

Supplementary Figure 5b

Flag

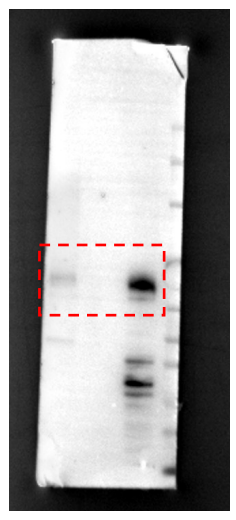

YAP

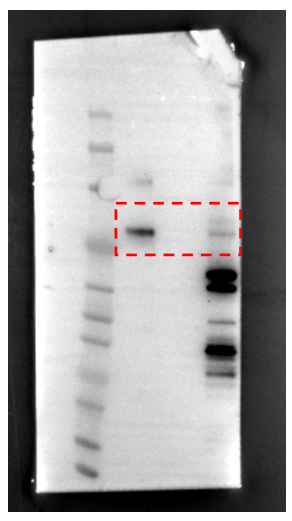

Supplementary Figure 5d

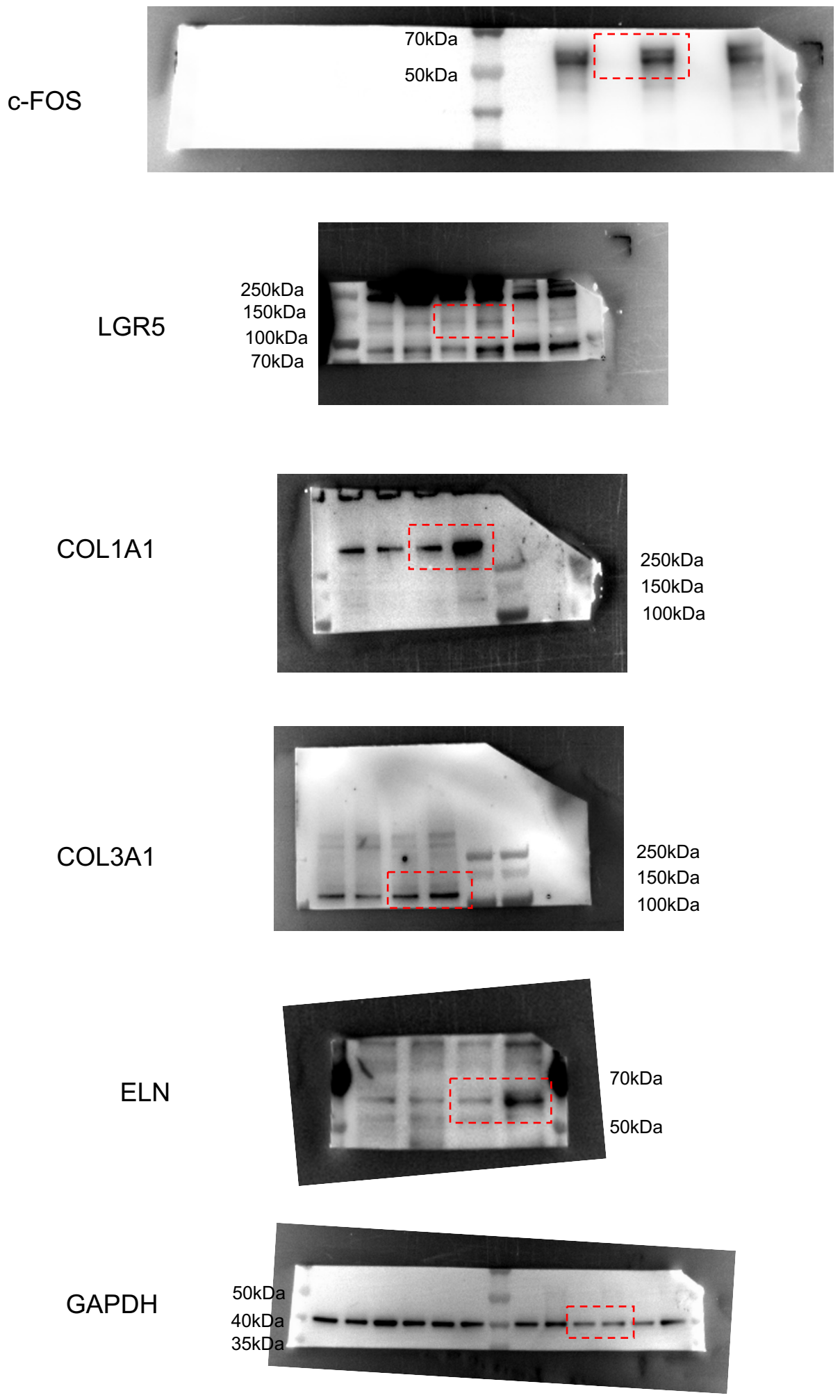

Supplementary Figure 5h

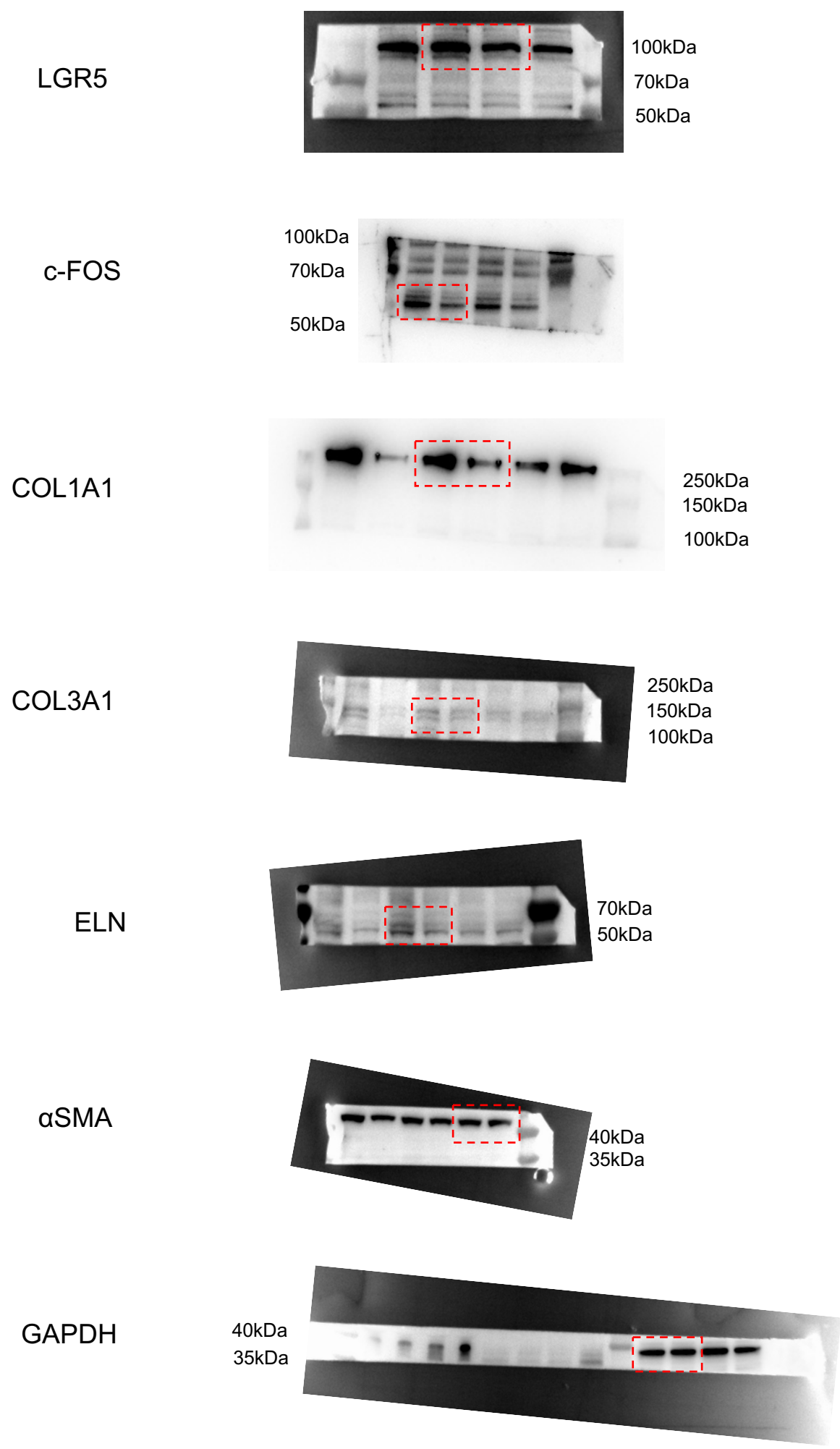

Supplementary Figure 8i

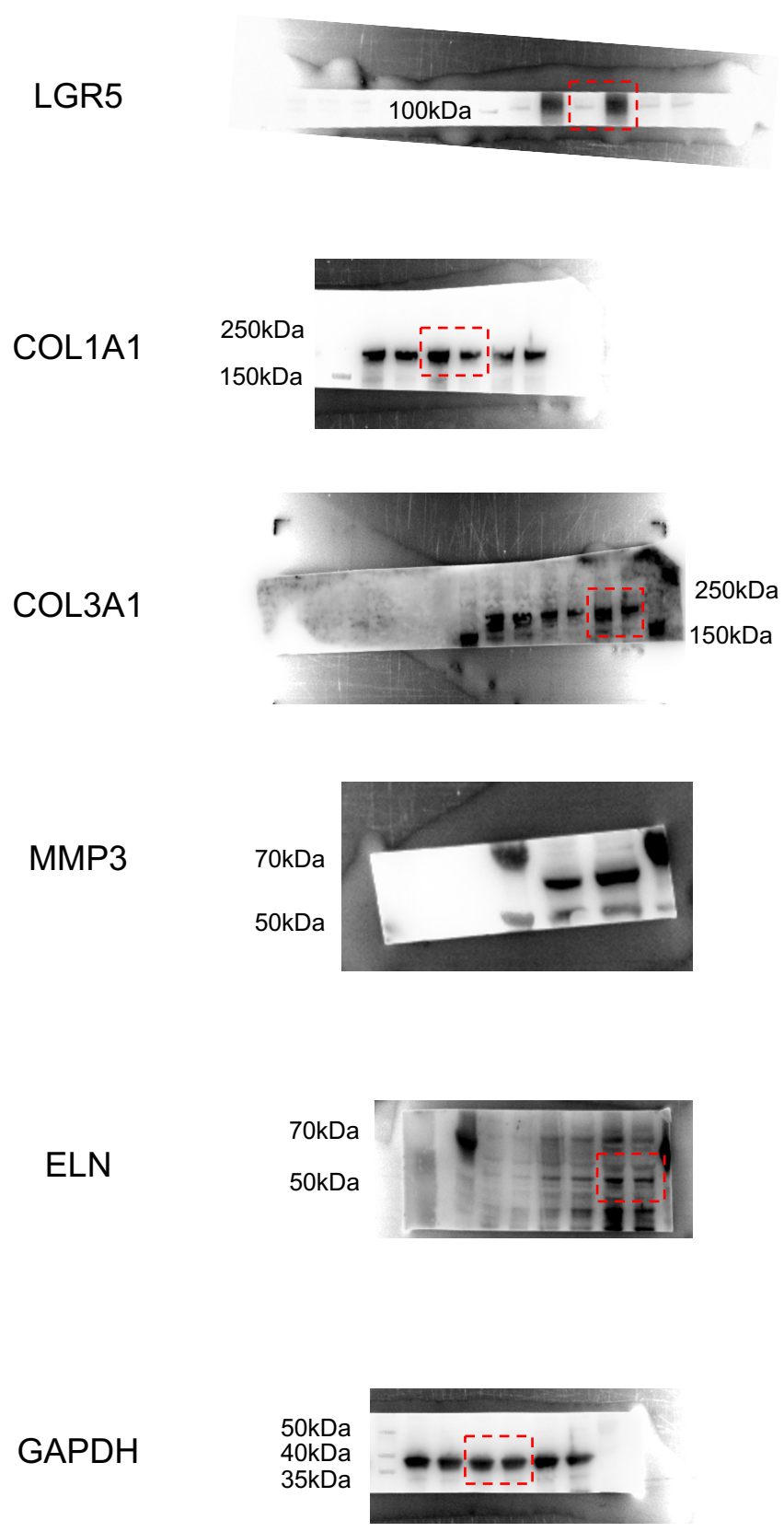

Supplementary Figure 8m

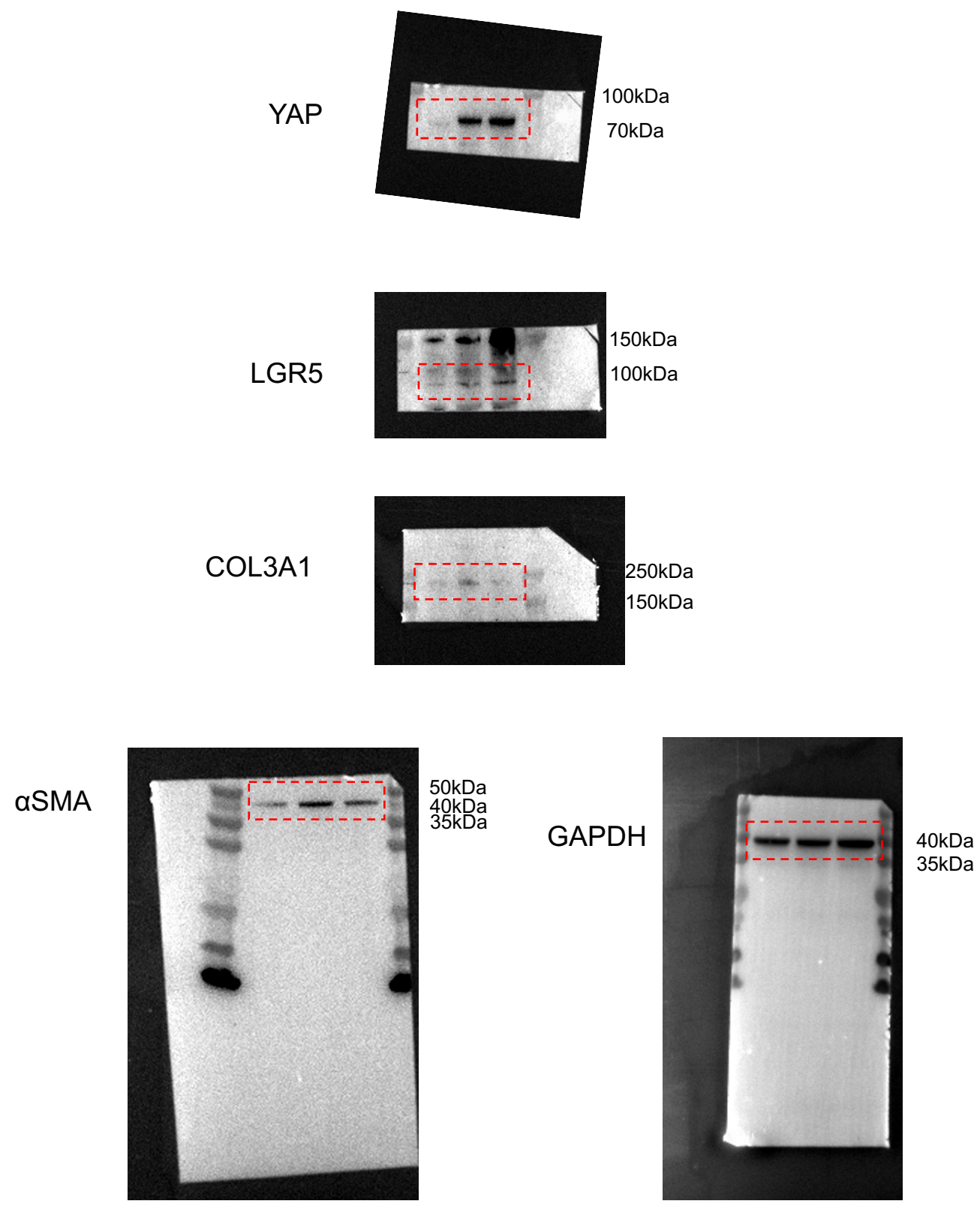

Supplementary Figure 9a

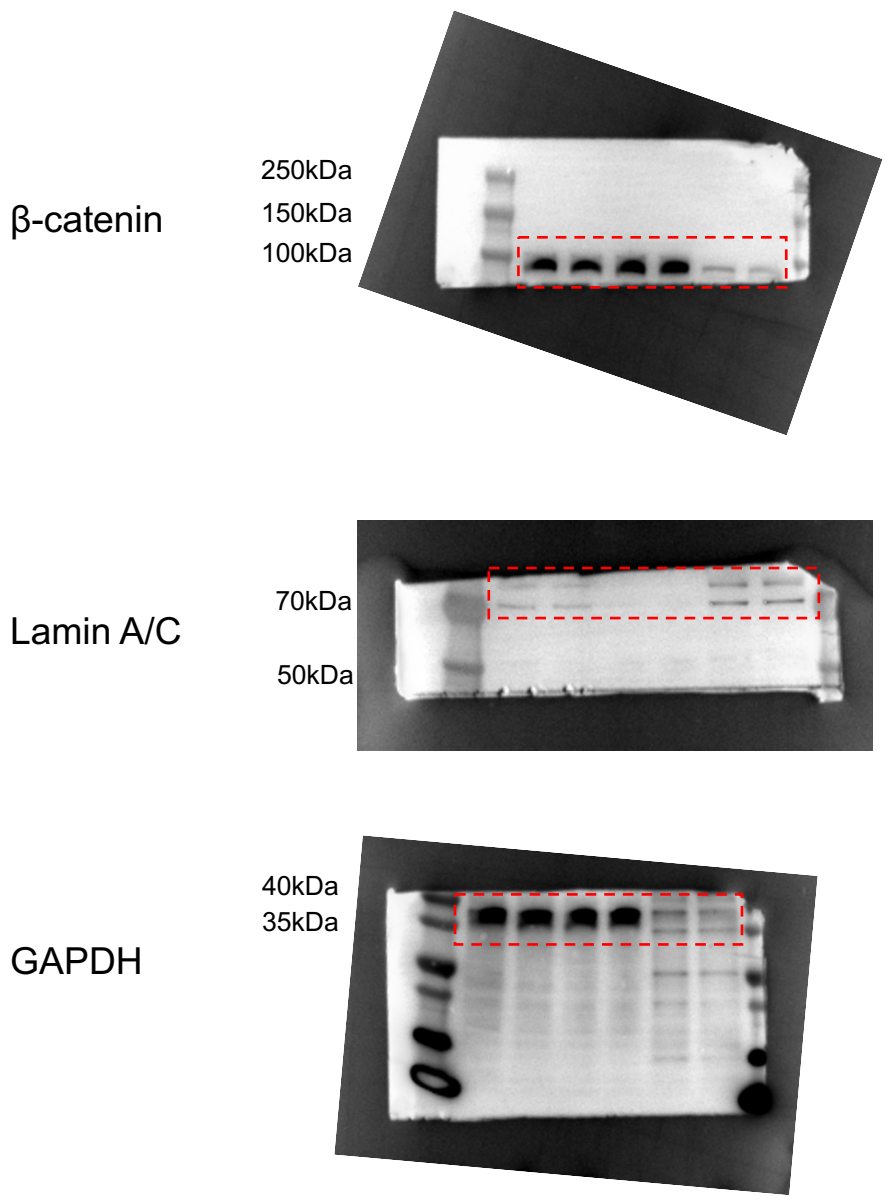

Supplementary Figure 9b

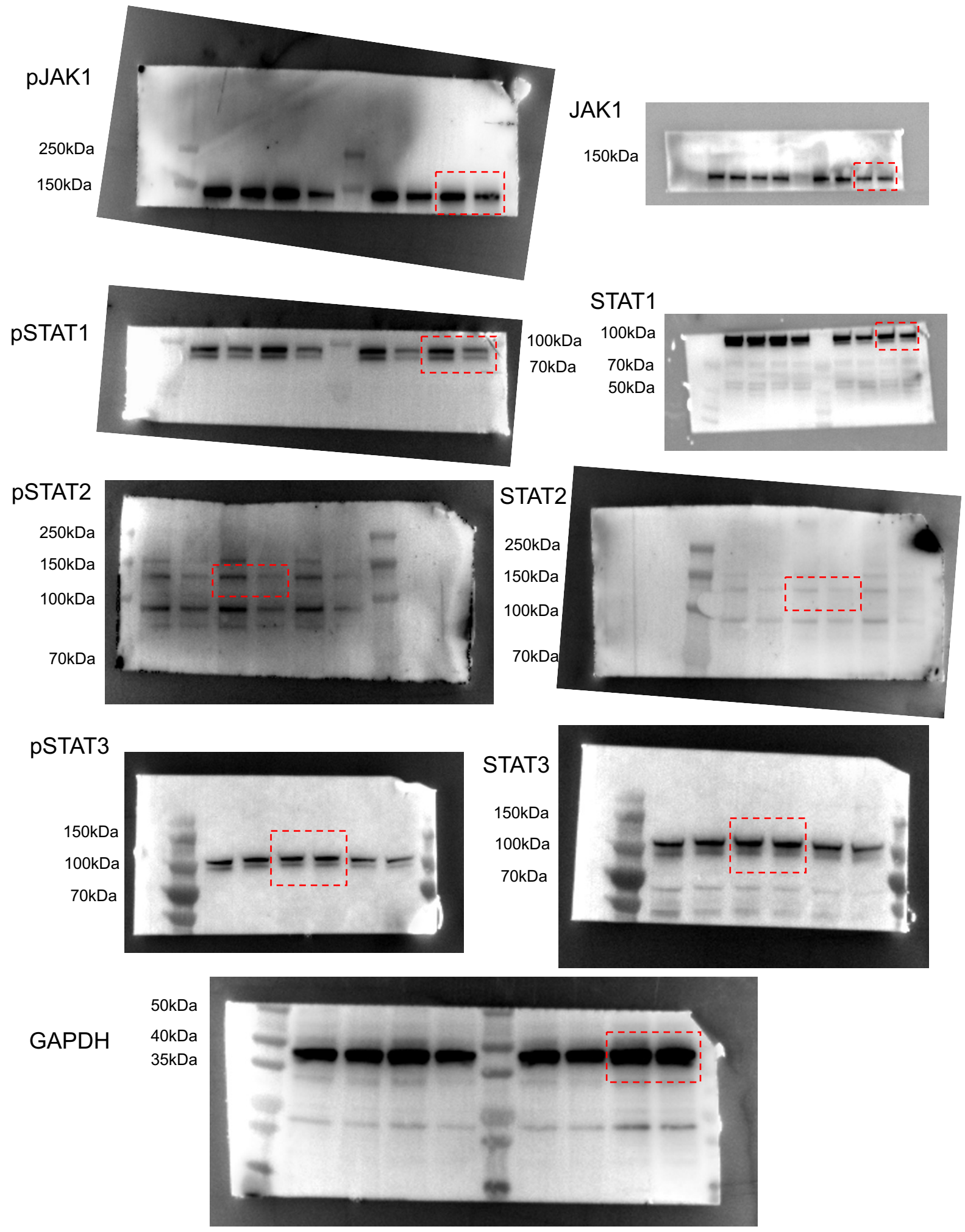

Supplementary Figure 9d

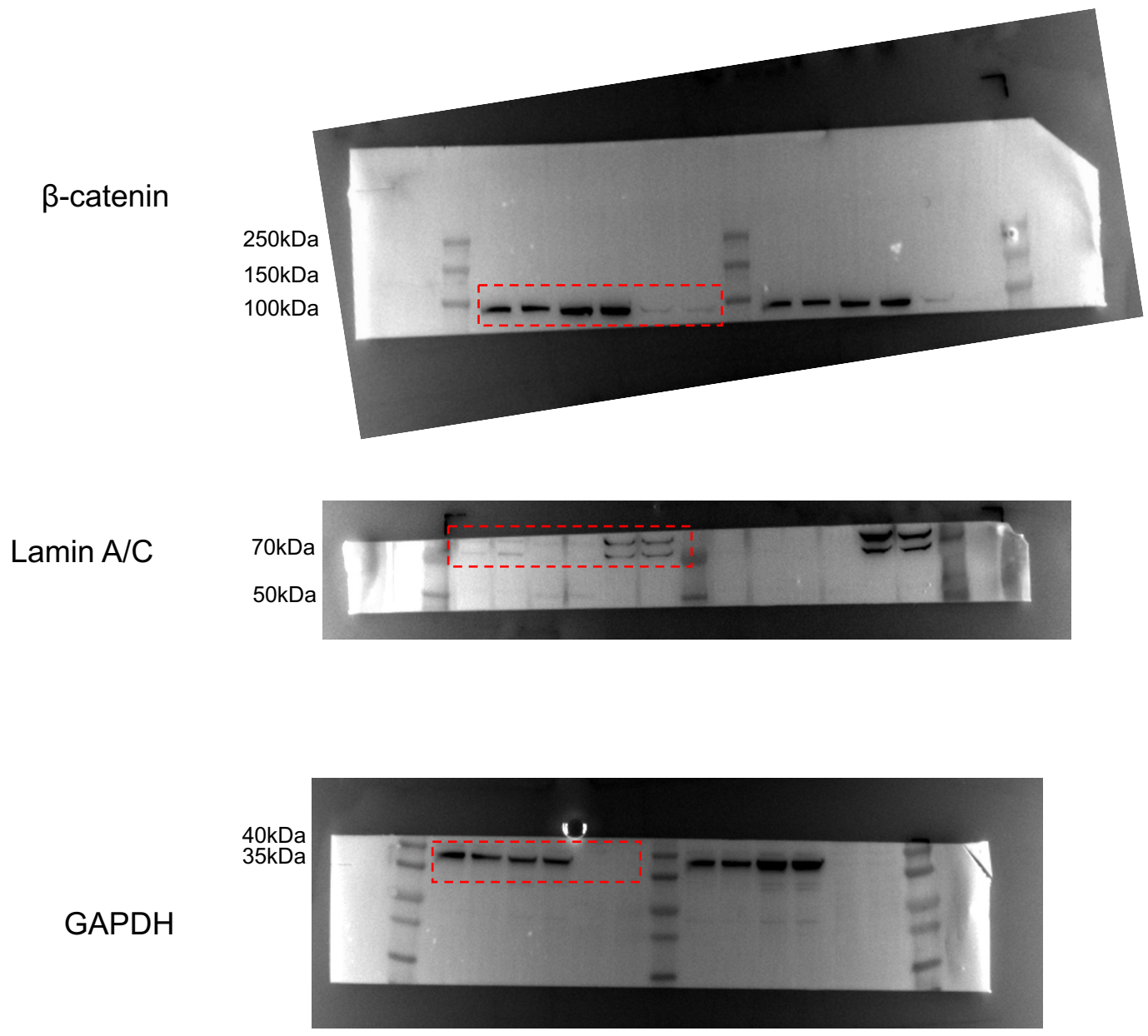

Supplementary Figure 9e

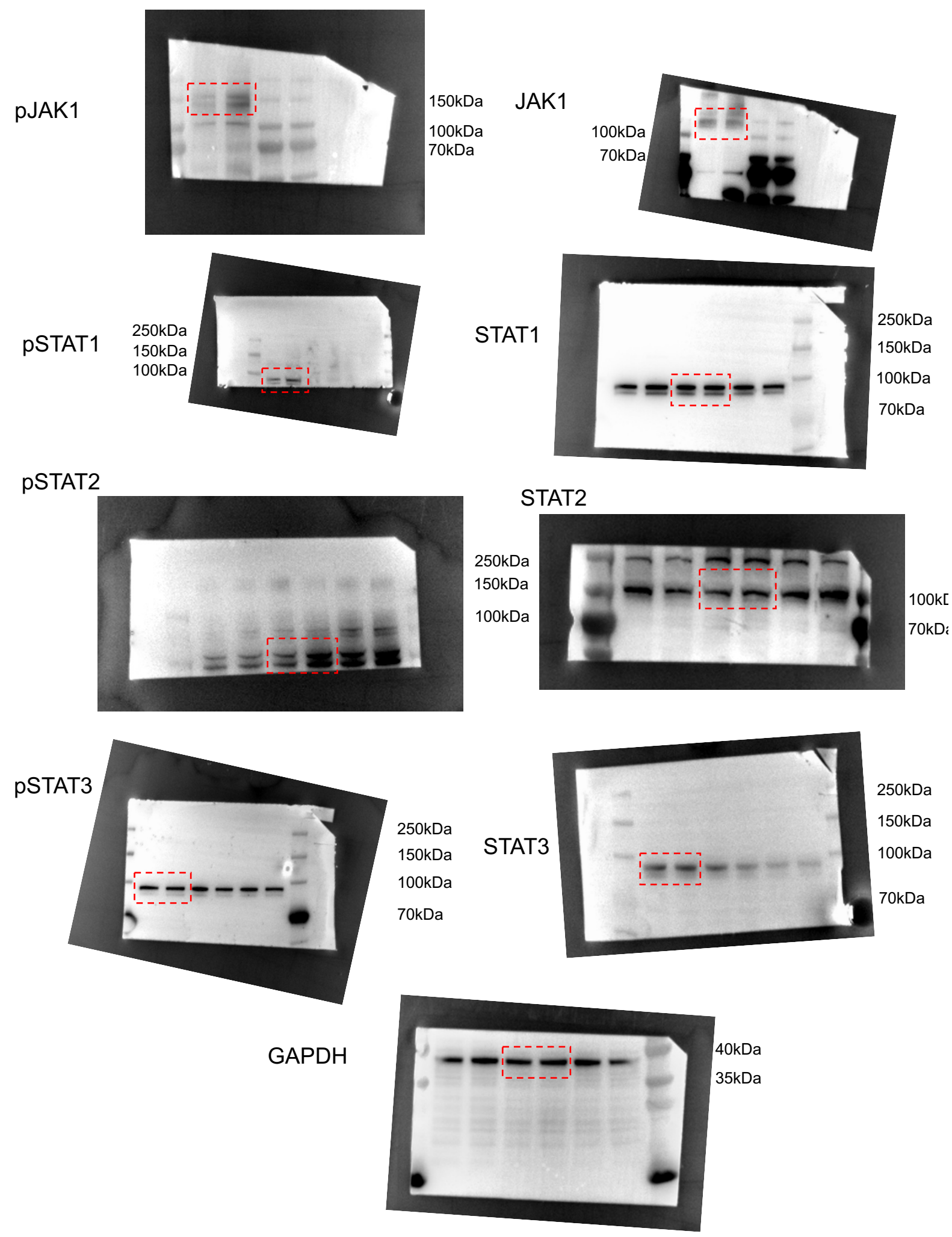

Supplementary Figure 9f

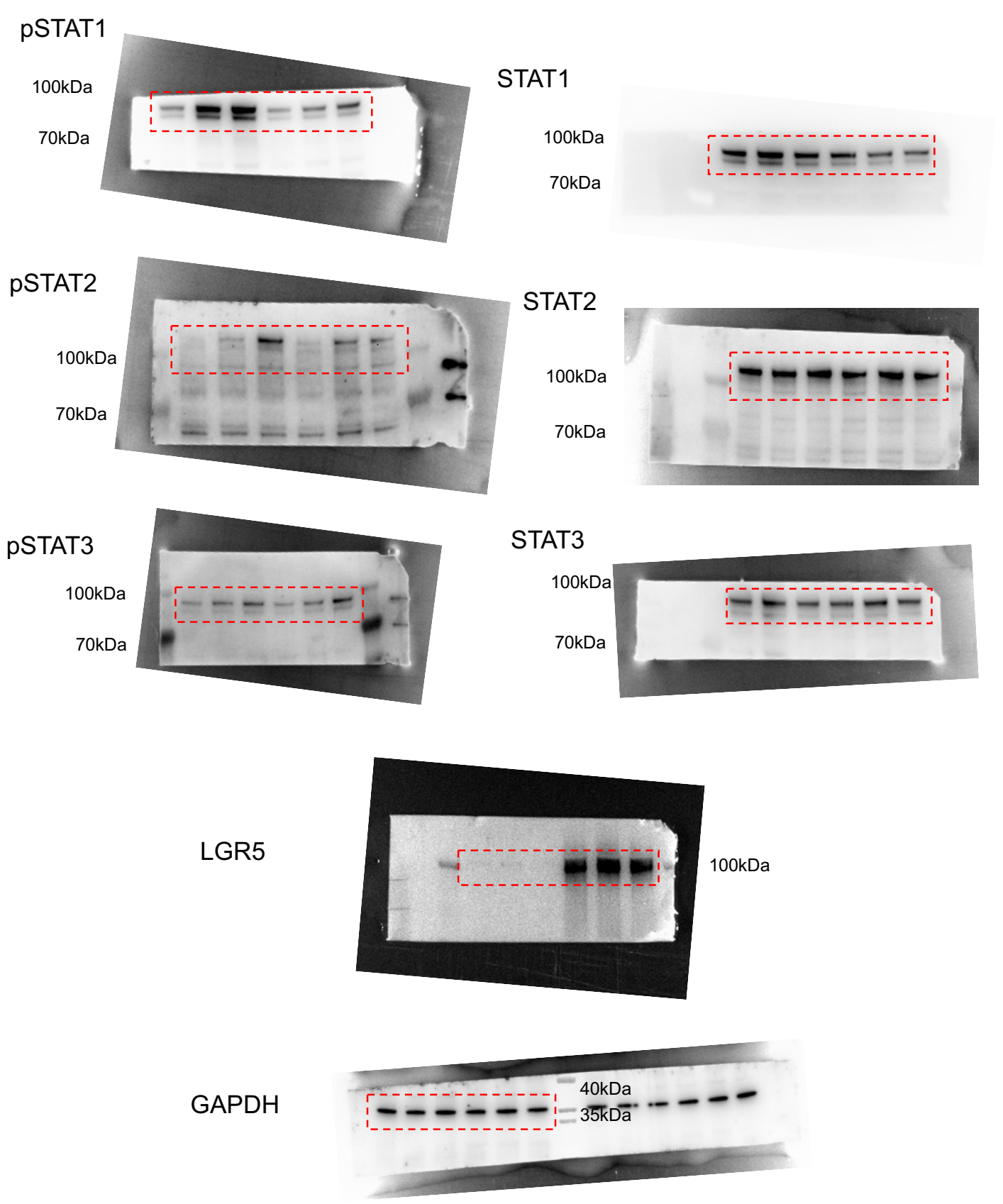

Supplementary Figure 9g

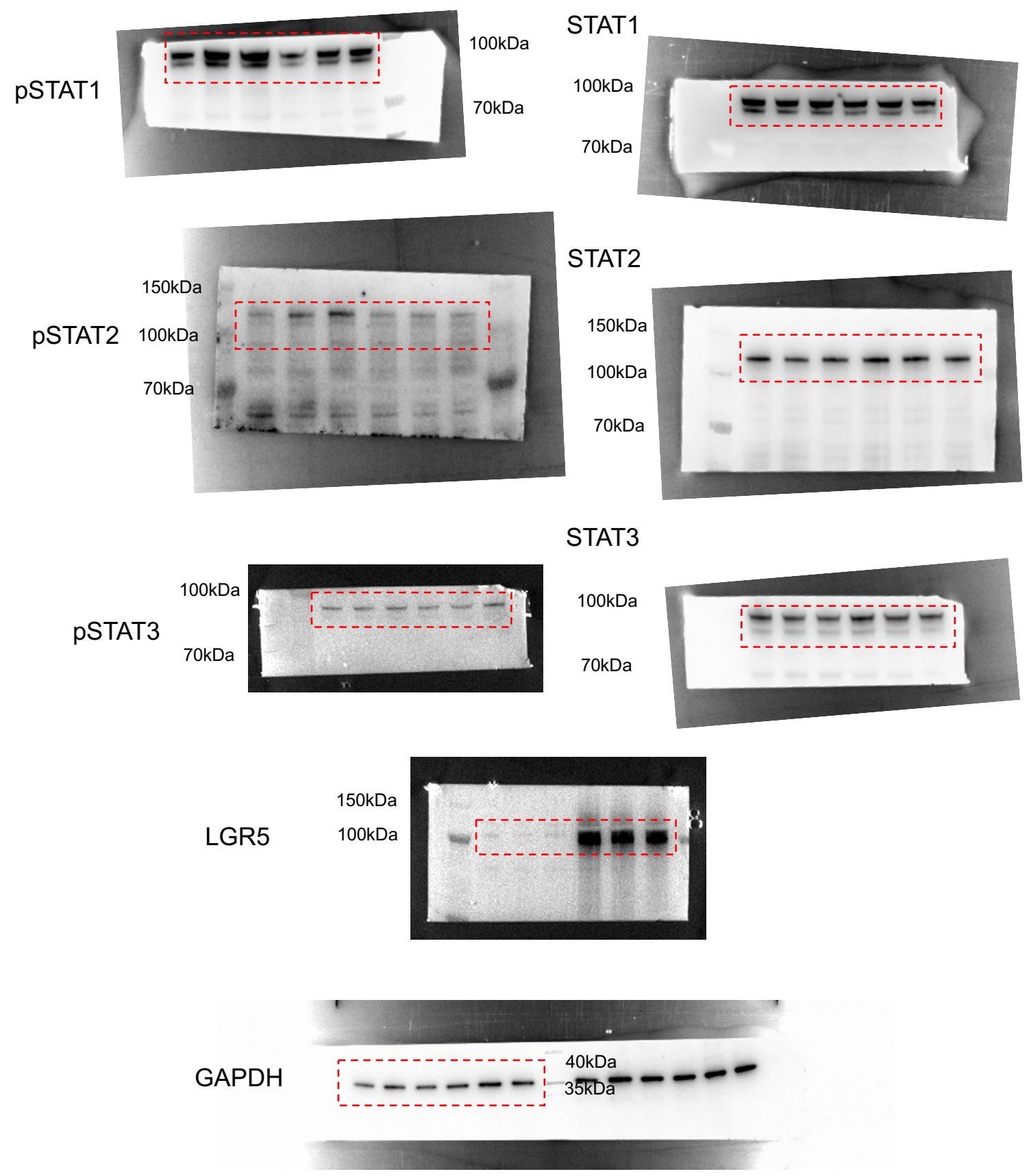

Supplementary Figure 9h

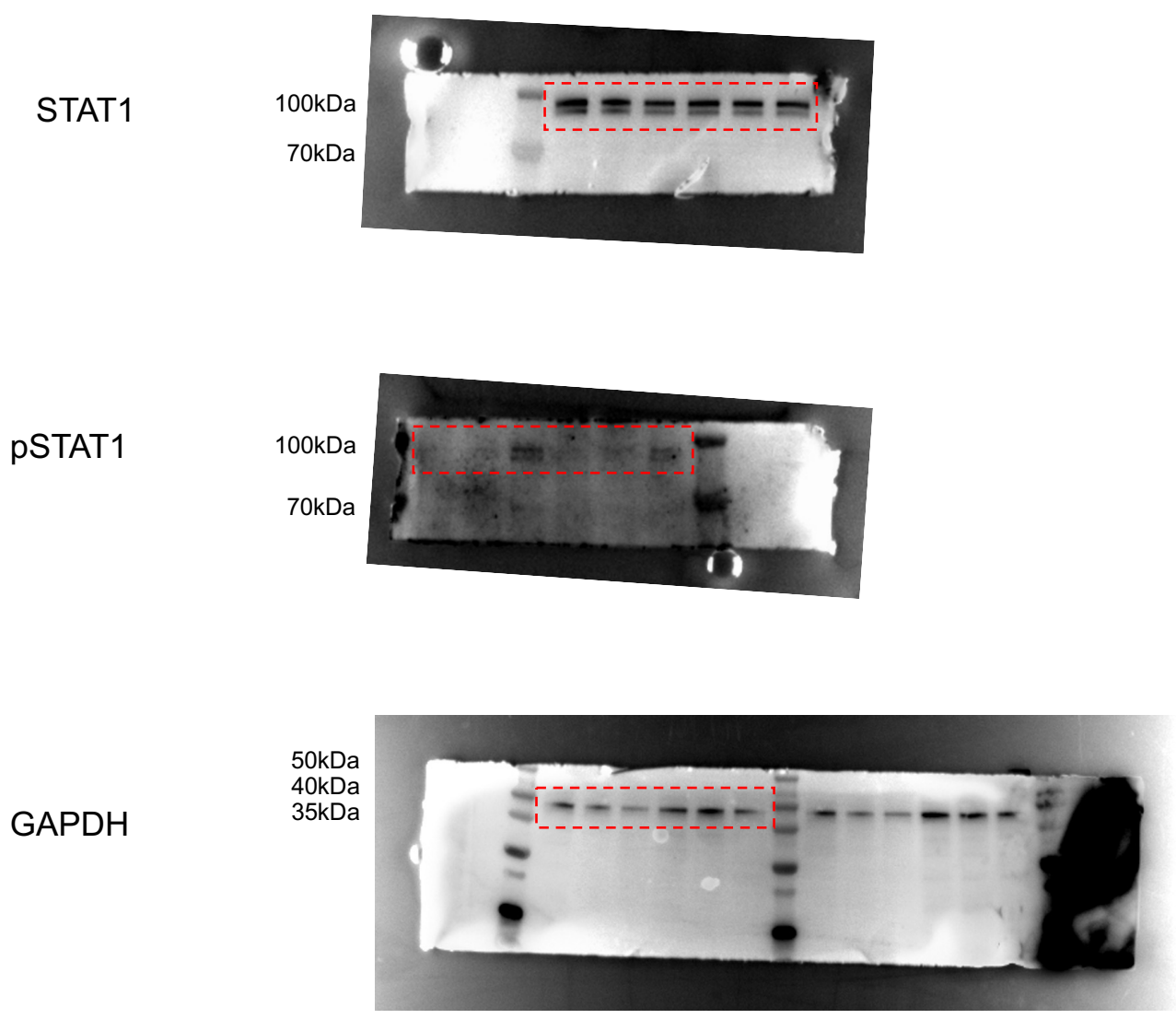

Supplementary Figure 9I

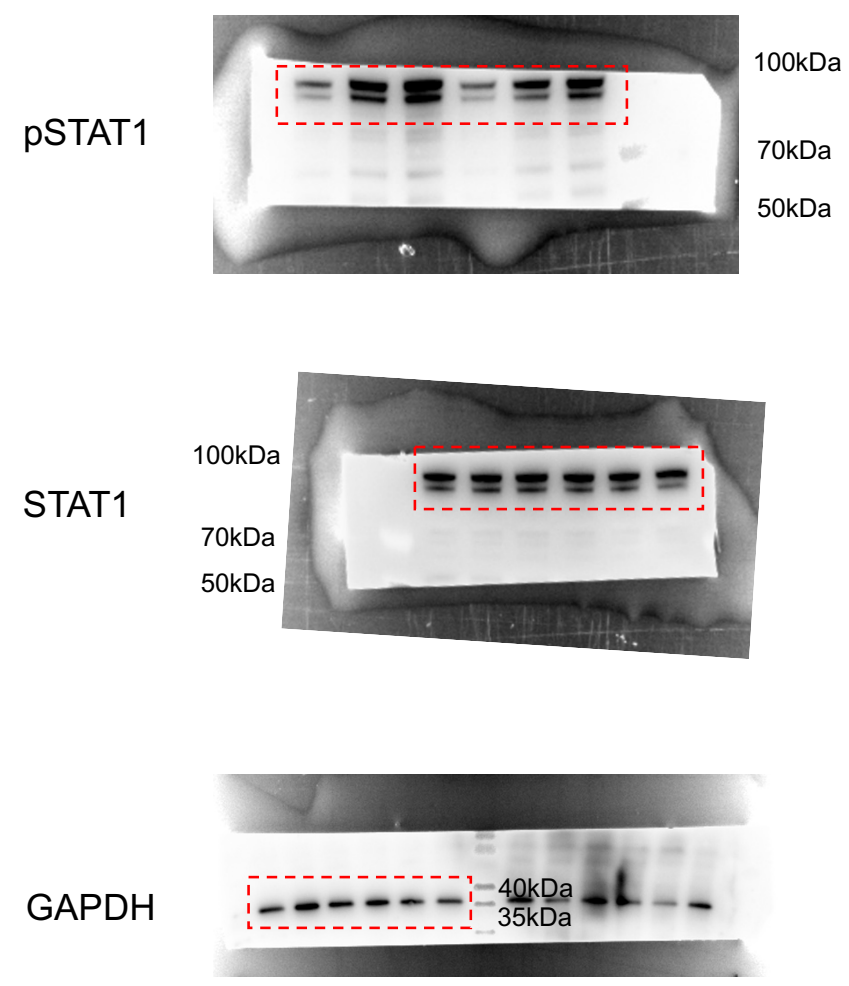

Supplement: Supplementary file 1 [file 41467_2026_77113_MOESM1_ESM.pdf]
