# Supplementary material for: LGR5-positive fibroblasts orchestrate skin mechanoadaptation through JAK1 modulation
Source: Nat Commun. 2026 Sep 11;17:10733. doi: 10.1038/s41467-026-77113-y (PMC13658777; doi:10.1038/s41467-026-77113-y)
Supplement: Supplementary file 2 [file 41467_2026_77113_MOESM2_ESM.pdf]

nature portfolio

Peer Review File

LGR5+ fibroblasts orchestrate skin mechanoadaptation through JAK1 modulation

Corresponding Author: Professor Caiyue Liu

This file contains all reviewer reports in order by version, followed by all author rebuttals in order by version.

Version 0:

Reviewer comments:

Reviewer #1

(Remarks to the Author)
This manuscript by Qiaoyu Fu and colleagues presents an ambitious and highly integrative study identifying a mechanically induced dermal fibroblast population marked by LGR5 that regulates adaptive tissue remodeling during mechanical stress. Using complementary mouse models of skin expansion, pressure loading, wound healing, pregnancy-associated stretch, and human mechanically stressed skin samples, the authors define LGR5+ mechanically induced regulatory fibroblasts (MIRFs) as a conserved mechanoresponsive stromal population with anti-fibrotic and anti-inflammatory functions.
The study combines a broad range of approaches including scRNA-seq reanalysis, lineage tracing, conditional genetic ablation and overexpression models, biomechanical measurements, enhancer analysis, CUT&RUN, pharmacologic perturbation, and translational rescue experiments with clinically relevant JAK inhibitors. Particularly impressive is the integration of mechanobiology with stromal signaling mechanisms and tissue-level functional outcomes. The identification of a YAP/AP-1-dependent enhancer program driving LGR5 expression, coupled with evidence for non-canonical suppression of JAK-STAT signaling through LGR5-JAK1 interaction, provides a compelling mechanistic framework linking mechanical stress to adaptive tissue remodeling.
The manuscript is conceptually strong, technically sophisticated, and potentially of broad interest to investigators working in mechanobiology, fibrosis, stromal biology, regenerative medicine, and tissue repair. The translational relevance is also a major strength, particularly the demonstration that topical JAK inhibition phenocopies several beneficial aspects of the LGR5 program during skin mechanoadaptation.
Overall, this is an elegant, multidisciplinary, and conceptually important study that substantially advances our understanding of stromal mechanoadaptation. The combination of in vivo genetics, mechanobiology, human relevance, and translational therapeutic insight makes the manuscript particularly compelling. The requested revisions are relatively minor and primarily aimed at clarifying interpretation and refining presentation rather than requiring substantial new experimentation.
Suggestions for improvement
1. Clarify the terminology surrounding “MIRFs”: The authors present convincing evidence that LGR5 marks a mechanically induced fibroblast state with important adaptive functions. However, it would help the reader if the manuscript more explicitly discussed whether MIRFs represent: a stable fibroblast subtype, a transient mechanically induced state, or a spectrum within existing fibroblast populations. A brief clarification in the Discussion would likely be sufficient and would avoid overinterpretation of fibroblast identity/state relationships.
2. Slightly temper the mechanistic language surrounding the LGR5–JAK1 interaction.The proposed non-canonical interaction between LGR5 and JAK1 is highly interesting and supported by multiple complementary approaches. However, because much of the interaction work relies on overexpression systems and co-immunoprecipitation, the authors may wish to modestly soften statements implying that the precise biochemical mechanism is fully resolved.
For example, wording such as: “LGR5 directly binds and suppresses JAK1” could potentially be softened to “LGR5 associates with and negatively regulates JAK1 signaling.” This would better align the mechanistic interpretation with the current level of evidence while preserving the major conceptual advance.
3. Expand discussion of context dependence in YAP-LGR5 signaling.The finding that YAP signaling induces a protective/adaptive LGR5 program is particularly interesting given the established association of YAP with fibrosis and tissue stiffening. The Discussion would benefit from a few additional sentences addressing: how adaptive versus maladaptive mechanotransduction states may diverge; whether chronic fibrosis may eventually overwhelm this protective circuit; and whether LGR5 induction might represent an early compensatory response. This would add nuance to an already compelling mechanistic model.

Reviewer #2

(Remarks to the Author)

In this study by Fu et al the authors dissected the role of Lgr5 positive dermal fibroblast in tissue repair with an incredible level of detail. By combining multiple omics techniques, transgenic models, protein modelling and clever combinations of well controlled in vitro experiments the authors are not only elucidating the function and regulation of Lgr5 controlling fibroblast behaviour but also uncover a new detailed mechanism of mechanical stress adaption response involving YAP and JAK/STAT signalling. The authors have very thoroughly excluded other related signalling pathways including Wnt signalling and other inducer. Intriguingly their final data point to the possibility that topical JAK inhibition mimics LGR5 function and is able to promote mechanical adaptation and reduce skin fibrosis. Overall this an elegant and well elaborated innovative study which reveals an exciting new function of Lgr5 in fibroblast responding to mechanical stress. This work is significant to the field and I have only some minor comments to add:

Minor comments
1. Labelling of graphs could be clearer when showing different treatment conditions especially when using different grey colours. See figure 2n, o and p. Perhaps you could try to label them all like in Fig 2r which is much clearer.
2. Edu positive cells very difficult to see in Figure 3i and 5i. It would be good to perhaps switch the colour allocation here.
3. In Figure 4K could you please clarify the quantification that was used? Is it fold change to Control ExpD8? While it is clear that changes in total immune cells are related to total dermal cells, it is not obvious if the changes in immune cell subtypes relates to immune cells or total dermal cells. As displayed currently it feels a bit misleading and perhaps it would be better to show each cell type in a separate graph.
4. Figure 5D it is unclear why a violine plot was selected for the gross appearance scoring. Also in Figure 5 h the aSMA signal is very difficult to see.
5. In Figure 5g the representative images of the gel contraction do not seem to reflect the quantification (especially between CTL/ Lgr5OE EPD20)
6. Figure 6e, here as well the pSTAT1 signal is very difficult to see. Please improve.
7. In Figure 7g, please show skin images with a horizontal orientation of the epidermis
8. Similarly it is not clear why for the semi quantitative appearance scoring a violine plot was chosen in Figure 7j.
9. Figure organization in the extended data could be improved, e.g. in Extended Figure 1 the panels feel distributed a bit randomly.
10. In Extended figure 3C, could you please clarify at what time point the skin was isolated. If this is in adult mice the detected dermal Dlk1 signal is a bit surprising as this fibroblast marker becomes quickly down regulated postnatally (see Driskell et al., Nature 2013).
11. Extended Figure 6 in b and d the graph labelling is not always clear. Also in Extended Figure 6m close up window is misaligned.
12. In Extended Figure 6n, the Lgr5OE representative H&E image is not showing a wound bed and is rather a section at the wound edge. There should be a visible scar tissue in both conditions. Also it is unclear if the Masson staining are showing wounded bed tissue or not. These images are clearly outside the wound bed. Any adult wound of that size should produce a scar that is lacking hair follicles.
13. Extended Figure 10c and g, figure orientation as mentioned above.

Version 1:

Reviewer comments:

Reviewer #1

(Remarks to the Author)
The authors have addressed my comments satisfactorily.

Reviewer #2

(Remarks to the Author)
Many thanks for addressing all my comments and congratulations again to this excellent study.

Open Access This Peer Review File is licensed under a Creative Commons Attribution 4.0 International License, which permits use, sharing, adaptation, distribution and reproduction in any medium or format, as long as you give appropriate credit to the original author(s) and the source, provide a link to the Creative Commons license, and indicate if changes were made.
In cases where reviewers are anonymous, credit should be given to 'Anonymous Referee' and the source.
The images or other third party material in this Peer Review File are included in the article's Creative Commons license, unless indicated otherwise in a credit line to the material. If material is not included in the article's Creative Commons license and your intended use is not permitted by statutory regulation or exceeds the permitted use, you will need to obtain permission directly from the copyright holder.
To view a copy of this license, visit https://creativecommons.org/licenses/by/4.0/

Reviewer 1

*This manuscript by Qiaoyu Fu and colleagues presents an ambitious and highly integrative study identifying a mechanically induced dermal fibroblast population marked by LGR5 that regulates adaptive tissue remodeling during mechanical stress. Using complementary mouse models of skin expansion, pressure loading, wound healing, pregnancy-associated stretch, and human mechanically stressed skin samples, the authors define LGR5+ mechanically induced regulatory fibroblasts (MIRFs) as a conserved mechanoresponsive stromal population with anti-fibrotic and anti-inflammatory functions. The study combines a broad range of approaches including scRNA-seq reanalysis, lineage tracing, conditional genetic ablation and overexpression models, biomechanical measurements, enhancer analysis, CUT&RUN, pharmacologic perturbation, and translational rescue experiments with clinically relevant JAK inhibitors. Particularly impressive is the integration of mechanobiology with stromal signaling mechanisms and tissue-level functional outcomes. The identification of a YAP/AP-1-dependent enhancer program driving LGR5 expression, coupled with evidence for non-canonical suppression of JAK-STAT signaling through LGR5-JAK1 interaction, provides a compelling mechanistic framework linking mechanical stress to adaptive tissue remodeling. The manuscript is conceptually strong, technically sophisticated, and potentially of broad interest to investigators working in mechanobiology, fibrosis, stromal biology, regenerative medicine, and tissue repair. The translational relevance is also a major strength, particularly the demonstration that topical JAK inhibition phenocopies several beneficial aspects of the LGR5 program during skin mechanoadaptation.
Overall, this is an elegant, multidisciplinary, and conceptually important study that substantially advances our understanding of stromal mechanoadaptation. The combination of in vivo genetics, mechanobiology, human relevance, and translational therapeutic insight makes the manuscript particularly compelling. The requested revisions are relatively minor and primarily aimed at clarifying interpretation and refining presentation rather than requiring substantial new experimentation.*

Suggestions for improvement
Comment 1:
Clarify the terminology surrounding “MIRFs”: The authors present convincing evidence that LGR5 marks a mechanically induced fibroblast state with important adaptive functions. However, it would help the reader if the manuscript more explicitly discussed whether MIRFs represent: a stable fibroblast subtype, a transient mechanically induced state, or a spectrum within existing fibroblast populations. A brief clarification in the Discussion would likely be sufficient and would avoid overinterpretation of fibroblast identity/state relationships.

Response 1:

We thank the reviewer for this thoughtful comment, which raises an important distinction between a stable fibroblast subtype and a transient mechanically induced state. We agree that the current evidence more cautiously supports defining LGR5 as a marker of a mechanically induced state rather than a fixed subpopulation. To avoid overinterpretation and align with the reviewer's suggestion, we have made the following changes:

1. Revision in "Introduction": "*Here, we identify LGR5, best known as an epithelial stem cell marker, as the defining feature of a conserved* ***mechanically induced state*** *of dermal fibroblasts that function as precisely such a brake.*"

2. Revision in "Results, section 1": "*Thus, LGR5 consistently marks a* ***mechanically induced state*** *of fibroblast in mouse and human skin.*"

3. Revision in "Discussion": "***LGR5⁺ MIRFs define a mechanically induced state that, once established, exhibits relative stability, even after load removal in certain cases.*** *Their persistence in mechanically compromised skin thus appears paradoxical.*" This clarifies that the state is mechanically induced yet can show relative stability after load removal, without claiming it as a permanent subtype.

These revisions explicitly address the reviewer's concern and refine the terminology throughout the manuscript. We believe this clarification appropriately reflects the spectrum nature of this fibroblast state.

Comment 2: Slightly temper the mechanistic language surrounding the LGR5–JAK1 interaction.The proposed non-canonical interaction between LGR5 and JAK1 is highly interesting and supported by multiple complementary approaches. However, because much of the interaction work relies on overexpression systems and co-immunoprecipitation, the authors may wish to modestly soften statements implying that the precise biochemical mechanism is fully resolved. For example, wording such as: "LGR5 directly binds and suppresses JAK1" could potentially be softened to "LGR5 associates with and negatively regulates JAK1 signaling." This would better align the mechanistic interpretation with the current level of evidence while preserving the major conceptual advance.

Response 2:

We thank the reviewer for this constructive suggestion. We agree that the current biochemical evidence, which largely relies on overexpression systems and co-immunoprecipitation, warrants a more cautious interpretation of the LGR5–JAK1 interaction. To better align our mechanistic language with the level of evidence, we have made the following revisions throughout the manuscript: In Result 7 and Figure 6, we have softened our description by replacing some "binding" with "association" or "interaction" and removed the term "direct" where appropriate. The relevant expressions now include: "*LGR5 inhibits fibroblast*

JAK-STAT activity via ***non-canonical JAK1 interaction***", "*We confirmed this* ***physical LGR5-JAK1 association*** *by co-immunoprecipitation*", "*Together, these findings define a non-canonical mechanism whereby* ***LGR5 interacts with JAK1 to negatively regulate JAK-STAT signaling***", etc. These revisions more accurately reflect the current level of evidence while preserving the major conceptual advance of our findings.

Comment 3: Expand discussion of context dependence in YAP-LGR5 signaling.The finding that YAP signaling induces a protective/adaptive LGR5 program is particularly interesting given the established association of YAP with fibrosis and tissue stiffening. The Discussion would benefit from a few additional sentences addressing: how adaptive versus maladaptive mechanotransduction states may diverge; whether chronic fibrosis may eventually overwhelm this protective circuit; and whether LGR5 induction might represent an early compensatory response. This would add nuance to an already compelling mechanistic model.

Response 3:

We thank the reviewer for this insightful suggestion. In the revised Discussion, we have added the following sentences to explicitly address the context dependence: "***We propose that the balance between adaptive and maladaptive mechanotransduction is dictated by stress intensity: moderate loads engage LGR5 as a protective brake, whereas excessive or chronic stress overwhelms this circuit, leading to pathological fibrosis and even macroscopic skin damage. Thus, LGR5 induction may represent a compensatory response with finite protective capacity that can be exhausted under extreme loading.***"

We believe this addition enhances the conceptual depth of our manuscript and addresses the reviewer's concern regarding context-dependent interpretability.

Reviewer 2

In this study by Fu et al the authors dissected the role of Lgr5 positive dermal fibroblast in tissue repair with an incredible level of detail. By combining multiple omics techniques, transgenic models, protein modelling and clever combinations of well controlled in vitro experiments the authors are not only elucidating the function and regulation of Lgr5 controlling fibroblast behaviour but also uncover a new detailed mechanism of mechanical stress adaption response involving YAP and JAK/STAT signalling. The authors have very thoroughly excluded other related signalling pathways including Wnt signalling and other inducer. Intriguingly their final data point to the possibility that topical JAK inhibition mimics LGR5 function and is able to promote mechanical adaptation and reduce skin fibrosis. Overall this an elegant and well elaborated innovative study which reveals an exciting new function of Lgr5 in fibroblast responding to mechanical stress. This work is significant to the field and I have only some minor comments to add:

Minor comments

Comment 1: Labelling of graphs could be clearer when showing different treatment conditions especially when using different grey colours. See figure 2n, o and p. Perhaps you could try to label them all like in Fig 2r which is much clearer.

Response 1:

We thank the reviewer for this suggestion. We have revised Figure 2n, o, p in two ways: (1) the fluorescence panel legends are sorted and re-grouped; and (2) the original grey-scale color coding has been replaced with more vivid, contrasting colors to clearly match statistical graphs with fluorescence images.

Comment 2: Edu positive cells very difficult to see in Figure 3i and 5i. It would be good to perhaps switch the colour allocation here.

Response 2:

We thank the reviewer for this comment. The poor visibility of EdU-positive cells in Fig. 4i and 5i (We note that EdU staining appears in Fig. 4i and 5i (rather than Fig. 3i, as the reviewer cited)) was due to resolution reduction to meet file size limits of initial manuscript submission. In our original high-resolution figures, EdU^+ cells are labeled in magenta, which clearly distinguishes them from blue DAPI-stained nuclei of EdU^- cells. We have now uploaded the original, full-resolution versions of all figures with the revised manuscript and have slightly adjusted the contrast to appropriately enhance the magenta signal. These improvements ensure that individual EdU-positive nuclei can be clearly identified.

Comment 3: In Figure 4K could you please clarify the quantification that was used? Is it fold change to Control ExpD8? While it is clear that changes in total immune cells are related to total dermal cells, it is not obvious if the changes in immune cell subtypes relates to immune cells or total dermal cells. As displayed currently it feels a bit misleading and perhaps it would be better to show each cell type in a separate graph.

Response 3:

We thank the reviewer for this careful observation. In the original version of Fig. 4k, the quantification was indeed presented as $\log_2$ fold change relative to the CTRL group at Day8 post expansion (EXPD8), with values normalized to total dermal cells. We acknowledge that this normalization strategy may have caused confusion.

To address this concern, we have revised Fig. 4k as follows: we now present the raw data directly, with the y-axis indicating the percentage of each indicated cell type within total dermal cells. This allows readers to directly compare the absolute abundance of each immune cell subset across conditions, eliminating any ambiguity. We opted to keep the combined format for compactness, but with the revised raw-data representation that we believe is more transparent and interpretable.

We have updated the figure legend accordingly to clarify the unit of measurement. We hope this revision addresses the reviewer's concern.

Comment 4: Figure 5D it is unclear why a violine plot was selected for the gross appearance scoring. Also in Figure 5 h the aSMA signal is very difficult to see.

Response 4:

We thank the reviewer for this comment. We previously selected violin plots for Fig. 5d to visualize the distribution of semi-quantitative data. To improve clarity, we have now replaced the violin plots with scatter plots showing individual data points (with median and interquartile range indicated). This change makes the data presentation more intuitive. The figure legend has been updated accordingly.

For Fig. 5h, as described in Comment 2, the difficulty in visualization was partly due to the resolution reduction required to meet file size limits. We have uploaded the original, high-resolution versions of figures with the revised manuscript. In addition, we recolored the αSMA signal to magenta, adjusted brightness and contrast uniformly across all conditions to enhance signal clarity. These improvements ensure that αSMA-positive myofibroblasts can be clearly identified. We apologize for the inconvenience and thank the reviewer for pointing this out.

Comment 5: In Figure 5g the representative images of the gel contraction do

not seem to reflect the quantification (especially between CTL/ Lgr5OE EPD20).

Response 5:

We thank the reviewer for this careful observation. We have now replaced the representative images for Fig. 5g with new images that better represent the mean gel contraction values shown in the quantification graph.

Comment 6: Figure 6e, here as well the pSTAT1 signal is very difficult to see. Please improve.

Response 6:

We apologize for the poor visibility of pSTAT1 signal in Fig. 6e. As with EdU and αSMA, we have now: (1) uploaded the original, high-resolution versions of figures with the revised manuscript; (2) recolored pSTAT1 with magenta; (3) adjusted brightness and contrast uniformly across both conditions. We believe that the revised Fig. 6e now clearly shows the pSTAT1 signal.

Comment 7: In Figure 7g, please show skin images with a horizontal orientation of the epidermis.

Response 7:

We thank the reviewer for this suggestion. The original orientation was chosen to fit the layout. Following the reviewer's advice, we have now reoriented the skin images in Figure 7g to show the epidermis horizontally (epidermis at the top, dermis below), which is the conventional orientation for skin histology.

Comment 8: Similarly it is not clear why for the semi quantitative appearance scoring a violine plot was chosen in Figure 7j.

Response 8:

We thank the reviewer for this comment. We previously selected violin plots for Fig. 7j to visualize the distribution of semi-quantitative scores. To improve clarity, we have now replaced the violin plots with scatter plots showing individual data points (with median and interquartile range indicated). This change makes the data presentation more intuitive. The figure legend has been updated accordingly.

Comment 9: Figure organization in the extended data could be improved, e.g. in Extended Figure 1 the panels feel distributed a bit randomly.

Response 9:

We thank the reviewer for this constructive feedback. We have reorganized the Extended Data figures to improve readability, including regrouping the

panels in Extended Data Fig. 1, 9 and 10.

Comment 10: In Extended figure 3C, could you please clarify at what time point the skin was isolated. If this is in adult mice the detected dermal Dlk1 signal is a bit surprising as this fibroblast marker becomes quickly down regulated postnatally (see Driskell et al., Nature 2013).

Response 10:

We thank the reviewer for this careful observation. We apologize for the insufficient labeling in the original figure. To clarify: the samples shown in Extended Data Fig. 3c were derived from adult mouse skin undergoing active mechanical expansion, rather than from unmanipulated resting skin. In this context, mechanical expansion and wound healing share similar mechano-activated microenvironments, both of which lead to the reactivation and expansion of $Dlk1^+$ reticular fibroblasts in the adult dermis (Driskell et al., 2013; Mascharak et al., 2021). Consistent with this, we have previously shown that fibroblasts in this expansion model closely resemble those in wound healing (Sun et al., 2025). Therefore, the widespread Dlk1 signal we observed is fully consistent with the literature and reflects active mechanoresponsive fibroblasts.

We have now modified the figure legend to avoid any confusion: "*c, Immunofluorescence staining for tdT (red) with Sca1 (grey) and Dlk1 (green) (left panels) or with Dpp4 (middle panels) in expanded skin from Lgr5-tracing mice (n = 4).*" We thank the reviewer for helping us improve the clarity of our data presentation.

References:

1. Driskell et al. Distinct fibroblast lineages determine dermal architecture in skin development and repair. Nature. 2013; 504 (277–281). doi:10.1038/nature12783.

2. Mascharak et al. Preventing Engrailed-1 activation in fibroblasts yields wound regeneration without scarring. Science. 2021; 372(6540). doi:10.1126/science.aba2374.

3. Sun et al. Mechanical Stretch-Induced Interlayer Coordination between MMP2 and COL17A1 Exacerbates Regenerative Exhaustion in Skin. Adv Sci. 2025; 12(41): e11474. doi: 10.1002/advs.202511474.

Comment 11: Extended Figure 6 in b and d the graph labelling is not always clear. Also in Extended Figure 6m close up window is misaligned.

Response 11:

We thank the reviewer for pointing out these issues. For Extended Data Fig. 6b and 6d, the original graphs used color coding that corresponded to the legend in panel c, which we acknowledge may not have been sufficiently clear. To address this, we have now modified these panels to display complete,

self-contained legends directly within or immediately adjacent to each graph, eliminating any ambiguity.

For Extended Data Fig. 6m, we apologize for the misalignment of the close-up window which occurred during figure assembly. We have now corrected the positioning of the white dashed box to ensure accurate representation of the region of interest.

Comment 12: In Extended Figure 6n, the Lgr5OE representative H&E image is not showing a wound bed and is rather a section at the wound edge. There should be a visible scar tissue in both conditions. Also it is unclear if the Masson staining are showing wounded bed tissue or not. These images are clearly outside the wound bed. Any adult wound of that size should produce a scar that is lacking hair follicles.

Response 12:

We sincerely thank the reviewer for the careful examination of our histological data. This comment is highly valuable, as it prompted us to revisit our histological sampling strategy and to more rigorously articulate the biological interpretation of the regenerative phenotypes observed in *Lgr5*^{cOE} wounds. We apologize for any ambiguity in our original figure presentation and offer the following clarifications. We also note that the panels discussed by the reviewer correspond to Extended Data Fig. 7 (rather than Extended Data Fig. 6). The following points address the reviewer's concerns.

First, our histological sampling covered the entire wound area. We provided low-magnification overviews of the full wound and used higher-magnification images from the wound center to visualize cellular details. The H&E and Masson's trichrome staining were performed on the same batch of samples, with images shown from different individuals to demonstrate reproducibility. These histological observations are fully consistent with the macroscopic views in Extended Data Fig. 7m, where the *Lgr5*^{cOE} wound bed shows markedly darker pigmentation indicative of hair neogenesis.

More importantly, this comment led us to recognize a compelling biological insight that we had not fully emphasized previously. The reviewer correctly notes that adult mouse wounds of this size typically heal with a fibrotic scar lacking hair follicles—and indeed, this is exactly what we observed in the control group. However, in *Lgr5*^{cOE} wounds, we consistently observed pigmented, hair-shaft-like structures emerging in the wound center, which are structures that are morphologically consistent with wound-induced hair follicle neogenesis (WIHN). Importantly, WIHN is not an artifact or an off-target effect; it is a well-documented regenerative phenomenon that occurs preferentially in large wound centers where a softer mechanical microenvironment exists (Harn et al., 2021; Harn et al., 2022; Oak et al., 2025; Xue et al., 2022). Our biomechanical data (Fig. 5f) directly demonstrate that *Lgr5*^{cOE} dermis exhibits reduced tissue stiffness during regeneration, providing a mechanistic basis for this WIHN-like

phenotype. Thus, rather than being an inconsistency, the presence of these structures in *Lgr5*^{cOE} wounds represents an important biological finding that supports our central conclusion: LGR5 promotes a softer, more permissive mechanical environment that favors regenerative healing over fibrosis.

To improve clarity, we have now included a brief explanation in the Results section: "*During dorsal wound healing, Lgr5*^{cOE} *accelerated late-stage healing and improved regenerative remodeling, mirroring wound-induced hair follicle neogenesis (WIHN) that favors a softened environment (Extended Data Fig. 7j-n), while tail skin healing—independent of mechanical tension—remained unaffected (Extended Data Fig. 7j-l).*" We thank the reviewer for this opportunity to improve the presentation of our data.

References:

1. Xue et al. Wound-Induced Hair Neogenesis Model. J Invest Dermatol. 2022 Oct;142(10):2565-2569. doi: 10.1016/j.jid.2022.07.013.
2. Harn et al. Symmetry breaking of tissue mechanics in wound induced hair follicle regeneration of laboratory and spiny mice. Nat Commun. 2021 May 10;12(1):2595. doi: 10.1038/s41467-021-22822-9.
3. Harn et al. Topological Distribution of Wound Stiffness Modulates Wound-Induced Hair Follicle Neogenesis. Pharmaceutics. 2022 Sep 12; 14(9): 1926. doi: 10.3390/pharmaceutics14091926.
4. Oak et al. Wnt signaling modulates mechanotransduction in the epidermis to drive hair follicle regeneration. Sci Adv. 2025 Feb 21;11(8):eadq0638. doi: 10.1126/sciadv.adq0638.

Comment 13: Extended Figure 10c and g, figure orientation as mentioned above.

Response 13:

We thank the reviewer for this suggestion. As with Figure 7g, we have now reoriented the skin images in Extended Data Fig. 10c and 10g to show the epidermis horizontally (epidermis at the top, dermis below).
