# Supplementary material for: LGR5-positive fibroblasts orchestrate skin mechanoadaptation through JAK1 modulation
Source: Nat Commun. 2026 Sep 11;17:10733. doi: 10.1038/s41467-026-77113-y (PMC13658777; doi:10.1038/s41467-026-77113-y)
Supplement: Supplementary file 3 [file 41467_2026_77113_MOESM3_ESM.pdf]

Description of Additional Supplementary Files

File Name: Supplementary Data 1
Description: IP–MS identified potential LGR5-interacting proteins in HFF

File Name: Supplementary Data 2
Description: A list of primer sequences for qRT-PCR assays

File Name: Supplementary Data 3
Description: A list of primer sequences for genotyping

File Name: Supplementary Data 4
Description: Publicly available datasets used in this study
